# Mechanically Robust, Self‐Healable, and Cryotolerant GelMA Hydrogel for Wearable Tactile Sensors with Enhanced Durability, Lifetime, and Environmental Stability

**DOI:** 10.1002/advs.77587

**Published:** 2026-09-16

**Authors:** Zhikang Li, Gengyu Han, Boqing Jia, Jijian Lu, Changxu Zhang, Bin Wang, Jiaxiang Wang, Kang Zhao, Muhammad Afzal Khan Qureshi, Lan Yu, Min Li, Guoxi Luo, Tong Wang, Qijing Lin, Weishi Li, Libo Zhao, Yumeng Xue

**Affiliations:** ^1^ School of Instrument Science and Technology Xi'an Jiaotong University Xi'an China; ^2^ State Key Laboratory for Manufacturing Systems Engineering State Industry‐Education Integration Center for Medical Innovations International Joint Laboratory for Micro/Nano Manufacturing and Measurement Technologies Shaanxi Innovation Center for Special Sensing and Testing Technology in Extreme Environments Shaanxi Provincial University Engineering Research Center for Micro/Nano Acoustic Devices and Intelligent Systems Xi'an Jiaotong University Xi'an China; ^3^ Department of Orthopaedics Peking University Third Hospital Beijing China; ^4^ Engineering Research Center of Bone and Joint Precision Medicine Peking University Third Hospital Beijing China; ^5^ Beijing Key Laboratory of Spinal Disease Research Peking University Third Hospital Beijing China; ^6^ School of Mechanical Engineering Xi'an Jiaotong University Xi'an China; ^7^ Shandong Laboratory of Advanced Materials and Green Manufacturing at Yantai Yantai China; ^8^ State Key Laboratory of Solidification Processing School of Materials Science and Engineering Northwestern Polytechnical University and Shaanxi Joint Laboratory of Graphene Xi'an China

**Keywords:** anti‐freezing, gelatin methacryloyl (GelMA) hydrogel, healthcare monitoring, mechanically robust, self‐healing, wearable ionotronic pressure sensor

## Abstract

Gelatin methacryloyl (GelMA) hydrogels are promising for wearable tactile sensors due to their biocompatibility, tissue‐mimic softness and conductivity, yet restricted by mechanical property, structure damage, dehydration, and freezing during usage. Herein, a mechanically robust, self‐healable, moisturizing, cryotolerant and adhesive GelMA hydrogel is developed by incorporating polyvinyl alcohol, acrylic acid, sodium tetraborate, and glycerol into GelMA matrix via a photoinitiated polymerization process. This unique approach overcomes the chemical‐mechanical conflictions among different molecules, and contributes to remarkable stretchability (≥220%, 5.5‐fold higher), elasticity (≈91.9% recovery), autonomous self‐healing within wide temperature range (from ‐40°C to 25°C), adhesion (≥23.5 kPa), moisture retention (≥81% after 10 d) and anti‐freezing (‐55.4°C) properties, significantly outperforming previous GelMA. Based on this, an omni‐healable ionotronic pressure sensor is constructed, featuring high sensitivity within broad pressure range (0–25 kPa), rapid recovery time, long‐term stability (≈120 h), excellent durability and performance self‐repairing (≈100% sensing range recovery) under subzero temperature. Sensitively and consistently monitoring of various physiological signals under harsh conditions, such as multiple structure damages, long period (≥5 d) and subzero temperatures (≤‐38.2°C) highlights its prominent durability, lifetime and environmental‐stability, demonstrating great promise toward practical application.

## Graphical abstract

A mechanically robust, self‐healable, moisturizing, cryotolerant and adhesive GelMA hydrogel is developed by incorporating polyvinyl alcohol, acrylic acid, sodium tetraborate, and glycerol into GelMA matrix via a photoinitiated polymerization process. The hydrogel‐derived omni‐healable ionotronic pressure sensor can sensitively and consistently monitor various physiological signals under harsh conditions, demonstrating great promise toward practical application.

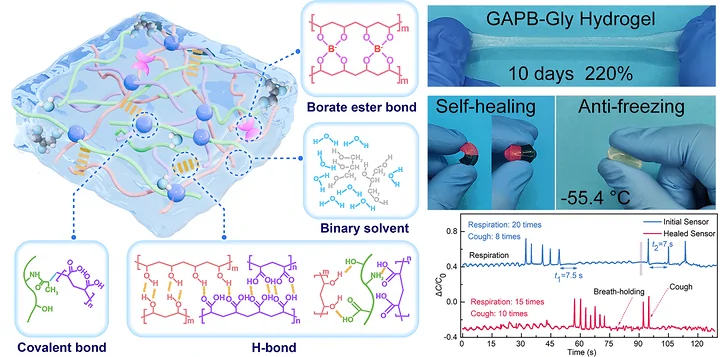

## 1 Introduction

Flexible wearable tactile sensors have witnessed booming advancement in recent years because they enable real‐time, low‐cost, and long‐term monitoring of human physiological signals, which are highly demanded in personalized healthcare and disease diagnosis of an increasingly aging population [1, 2, 3]. Among various materials used for wearable tactile sensors (e.g., textiles, polydimethylsiloxane (PDMS), and polyurethane (PU)), hydrogels show immense promise in developing next‐generation devices owing to their tissue‐mimic Young's modulus, tunable biological activities, and intrinsic ionic conductivity [4, 5, 6, 7]. These unique features endow wearable tactile sensors with ideal mechanical‐matching, biocompatibility, and efficient electrical communication at skin‐device interfaces. In this context, a diversity of hydrogel‐based tactile sensors, capable of detecting pressure, strain, and humidity, have been successfully demonstrated in wearable medical monitoring [8, 9, 10]. Despite these achievements, hydrogels still suffer severe challenges before practical application. First, most of synthetic hydrogels suffer inferior stretchability and elasticity because of their inhomogeneous network and deficiency in energy dissipation mechanism, which degrade the sensing performance [11, 12, 13]. Second, hydrogels are susceptible to external mechanical damages (such as scratch, wear, tear, and puncture) owing to their low Young's modulus, leading to limited reliability and longevity [14, 15]. Third, hydrogel‐based electronics generally lose their function under harsh environments (such as winter, high‐latitude regions and dry ambient condition) due to low‐temperature brittleness and water evaporation [16, 17].

Among different hydrogels, gelatin methacryloyl (GelMA), derived directly from the mammalian skin, has attracted extensive research because of their superior biochemical and mechanical properties. Unlike conventional synthetic hydrogels, GelMA inherently retains natural cell‐adhesive motifs (e.g., RGD sequences) that endow it with exceptional biocompatibility for long‐term and non‐irritating attachment on skin. Crucially, its tissue‐mimicking Young's modulus (1–20 kPa) and high tailorability enable it closely matches that of human epidermis, guaranteeing seamless, conformal contact with skin and minimizing interfacial signal artifacts during continuous physiological monitoring [18, 19]. Furthermore, GelMA contains abundant reactive functional groups that can be readily modified, allowing for developing soft electronics with multiple functions. However, as mentioned above, GelMA hydrogel still suffers from unmet performances in mechanical stretchability, elasticity, fracture resistance, cryotolerant and moisturizing abilities, which severely impeded its application in wearable sensors [20, 21]. To improve these properties, a large number of research has been carried out in the past few years. For instance, Gao et al. introduced poly(N‐acryloyl‐2‐glycine) into GelMA, leveraged its dual hydrogen bonds (H‐bonds) to strengthen the weak GelMA network and achieved a high tensile strength of 1.1 MPa, compressive strength of 12.4 MPa, and Young's modulus of 320 kPa [22]. Some researchers tried to endow GelMA with self‐healing property to resist its mechanical damage during usage and thereby expand lifetime. For example, Hafezi et al. developed a self‐healing GelMA hydrogel by integrating oxidized alginate and nano‐clay into the GelMA network. The introduced reversible Schiff base reactions between amino and aldehyde groups enabled dynamic covalent bonds re‐construction at damaged sites, while nano‐clay contributed additional reversible H‐bonds. This synergistic mechanism facilitated efficient self‐repair capability, demonstrating a self‐healing efficiency (SHE) of 74% in tensile modulus without external stimuli [23]. Wang et al. utilized cyclodextrin and adamantine‐derived host–guest supramolecules as multifunctional crosslinkers to copolymerize with GelMA, and embedded non‐covalent host–guest interactions into a covalently bonded network. As a result, both the mechanical property and self‐healing capability were significantly enhanced, exhibiting a fracture stress of ≈200 kPa after healing [24]. In addition, researchers also focused on improving the moisturizing and anti‐freezing properties of GelMA hydrogels. Wang et al. incorporated LiCl into inverse opal GelMA structures to transform free water into bound states, and achieved stable optical output at ‐20°C for 48 h [25]. In all, previous work has made significant progress in enhancing the mechanical strength, resilience, anti‐freezing, and anti‐drying properties of GelMA hydrogels [26, 27, 28, 29, 30]. However, integrating all these diverse traits into a single GelMA matrix remains a formidable challenge, primarily due to intrinsic molecular‐level trade‐offs among crosslinking chemistry, polymer chain mobility, intermolecular interactions and network architecture [26]. For instance, a fundamental paradox exists between mechanical robustness and self‐healing capacity. Increasing the density of covalent crosslinks enhances stiffness and strength, but inevitably restricts polymer chain mobility, thereby suppressing the dynamic rearrangement required for self‐healing [31]. Conversely, networks relying on reversible interactions exhibit improved healing efficiency but generally lack sufficient energy dissipation, resulting in compromised toughness and stretchability [24]. Second, improving environmental stability typically compromises structural integrity. Although hygroscopic or cryoprotective additives can stabilize water under extreme conditions, yet they perturb H‐bonds networks and weaken structural integrity [32]. Moreover, achieving strong and durable interfacial adhesion typically requires a high density of exposed functional groups, however, which may interfere with the internal cohesive network and reduce mechanical stability. Therefore, strategies that can simultaneously endow GelMA hydrogels with aforementioned multiple properties are urgently needed for their practical application in wearable sensors.

In this study, a GelMA‐based hydrogel with self‐healing, stretchability, and adhesiveness, anti‐drying, and cryotolerant properties was developed by precisely incorporating polyvinyl alcohol (PVA), acrylic acid (AA), sodium tetraborate (borax), and glycerol into the GelMA matrix. A unique strategy, which integrated covalent bonds, abundant multiple H‐bonds, and AA‐strengthened dynamic borate ester bonds (BEBs), along with glycerol/H_2_O binary solvent, was proposed to overcome the intrinsic trade‐offs among aforementioned multiple properties. Specifically, the AA (H^+^)‐strengthened BEBs and H‐bonds functioned as sacrificial bonds for energy dissipation under deformation, and combined with strong covalent bonds to synergistically enhance the mechanical elasticity, strength, and stretchability. Meanwhile, the reversible H‐bonds and tailored dynamic BEBs enabled the hydrogel to autonomously restore its integrity, while glycerol‐mediated interactions imparted moisture retention and antifreezing capabilities. Moreover, polymerized AA (PAA) and PVA‐induced dense congregation of anionic carboxylate and hydroxyl groups enabled robust interfacial adhesion with diverse substrates through forming H‐bonds or metal coordination interactions. Compared to pure GelMA hydrogel, the resultant GelMA/AA/PVA/borax‐glycerol (GAPB‐Gly) hydrogel achieved remarkable stretchability (≥220%, 5.5‐fold higher), high elasticity (≈91.9% in elastic recovery rate), considerable SHE within a wide temperature range (‐40°C to 25°C), strong and repeatable adhesion (≥23.5 kPa, 10 cycles), excellent moisture retention (≥81% after 10 days) and anti‐freezing (‐55.4°C) capability, and superior biological compatibility. Its comprehensive performance significantly surpassed the state‐of‐the‐art multifunctional hydrogels, including GelMA/PVA/NAGA/borax/NaCl in our previous work [26] and the PAA/PVA/borax/ethylene glycerol developed by Huang et al. [27] (Table S1 for detailed comparison). Encouraged by these prominent properties, a GAPB‐Gly based omni‐healable ionotronic capacitive pressure sensor was developed, demonstrating rapid response/recovery times, and outstanding durability, long‐term stability, excellent cryo‐resistance and performance recovery capability after damage.

## 2 Results and Discussion

### 2.1 Preparation, Mechanism, and Property Tunability of Multifunctional GAPB‐Gly Hydrogel

**FIGURE 1:**
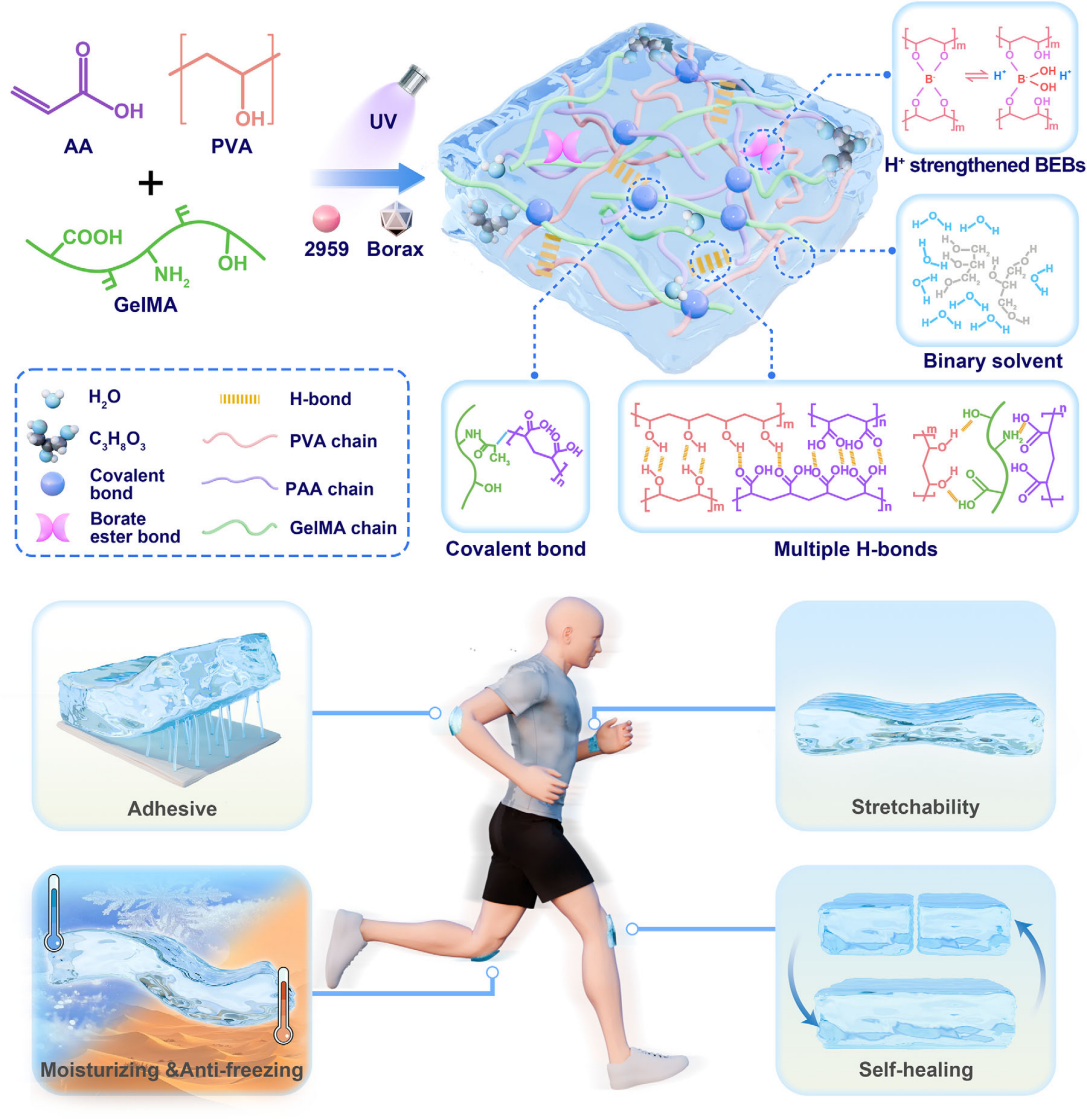
Synthesis process and mechanisms of multifunctional GAPB‐Gly hydrogel. a) Synthesis schematic and underlying interaction mechanisms. b) Multifunctional properties of the GAPB‐Gly hydrogel.

As depicted in Figure 1a, the GAPB‐Gly hydrogel was synthesized by introducing AA, borax, PVA, and glycerol/H_2_O binary solvent into GelMA solution and conducting one‐step free radical photopolymerization under UV irradiation. Figure S1 shows its quick transformation from the mixed solution to the final gel state. The ingenious integration of these components endowed the resultant gel with superior comprehensive properties, including self‐healing, stretchability, self‐adhesion, moisturizing, and anti‐freezing capability (Figure 1b). Particularly, the hydroxyl groups (‐OH) in the PVA generated a “di‐diol” complexation with borax ions, forming dynamic BEBs. Meanwhile, the PAA with rich carboxyl groups (‐COOH) provided a mildly acidic microenvironment, which promoted the protonation‐induced transformation of tetrahedral boronic esters into planar triangular configurations, and significantly enhanced the dynamic reversibility of BEBs, thus the elasticity and stretchability [33]. The interactions among GelMA, PVA, and PAA chains introduced abundant multiple H‐bonds, which acted as sacrificial bonds to dissipate energy during deformation, and further enhance the stretchability. Meanwhile, the GelMA and PAA chains contributed to construction of covalent bonds. As a result, the synergistic effect of the BEBs, multiple H‐bonds and covalent bonds imparted the resultant gel with excellent mechanical elasticity, strength, and stretchability. The reversible nature of multiple H‐bonds and BEBs enabled the hydrogel to autonomously self‐heal after mechanical damage. Furthermore, the introduced glycerol could form a large number of H‐bonds with water molecules and resulted in a significant reduction of free water in the matrix, which effectively hindered the water molecules evaporation and enhanced the moisturizing capacity. Besides, the reduction of free water in the gel matrix and the increase of the H‐bonds density could also lower the freezing point and improve the resistance to a low‐temperature environment. Additionally, the PAA and PVA‐induced dense congregation of anionic carboxylate and hydroxyl groups, endowed the hydrogel with robust interfacial adhesion capability through forming H‐bonds or metal coordination interactions with diverse substrates (Figure S2). Therefore, the proposed unique design strategy implemented a fine balance of different types of properties through precise molecular‐level interaction programming, conferring the GelMA hydrogel with successful integration of multifunctionality, and meeting the complex requirements of wearable tactile sensors in practical usage.

The successful synthesis of GAPB‐Gly hydrogel was verified by chemical structure characterization using nuclear magnetic resonance (NMR), Fourier transform infrared spectroscopy (FTIR), and X‐ray photoelectron spectroscopy (XPS). As shown in Figure S3, the appearance of characteristic peaks of methacrylic anhydride (5.43 and 5.67 ppm) and the distinct decrease in the methylene proton signal of lysine side chains (3.01 ppm) confirmed the successful grafting of methacryloyl (MA) onto the gelatin chains [21]. Based on the decrease in signal intensity of the lysine methylene protons at *δ* = 3.01 ppm, the degree of substitution of MA was calculated to be 83% [23]. The relatively high MA grafting ratio ensured effective covalent crosslinking of GelMA, enabling formation of a densely crosslinked network, thereby achieving high mechanical performance [25]. FTIR analysis revealed a gradual shift of O–H stretching peak from 3336 cm^−1^ to 3316 cm^−1^ (GPB), 3295 cm^−1^ (GAPB) and 3305 cm^−1^ (GAPB‐Gly) following the introduction of PVA‐borax, AA and glycerol, indicating enhanced H‐bonding between the different components (Figure S4) [26, 34, 35]. XPS analysis further confirmed the compositional evolution and new bond formation (Figure S5). In the high‐resolution C 1s spectrum (Figure S6a), incorporation of PVA increased the relative content of C–C bonds (284.5 eV), while introduction of AA increased the proportion of oxygen‐containing functional groups (e.g., –COOH), leading to a relative change in the C–C peak intensity [24]. A similar trend was observed in the O 1s XPS spectrum of different samples (Figure S6b). Notably, the emergence of a new peak at 192 eV in the B 1s XPS spectra of GPB indicated the formation of BEBs, which disappeared upon introduction of excess AA (60% v/v), highlighting the important role of AA in regulating the reversibility and strength of dynamic BEBs (Figure S6c) [36]. This is due to the abundant carboxyl groups on PAA chains exhibited high ionizability, releasing H^+^ in aqueous environments, which lowered the pH, affected the dissociation and reformation dynamics of BEBs. Collectively, these results demonstrated that the GAPB‐Gly hydrogel possesses a hybrid crosslinked structure composed of stable covalent frameworks and reversible dynamic bonds.

**FIGURE 2:**
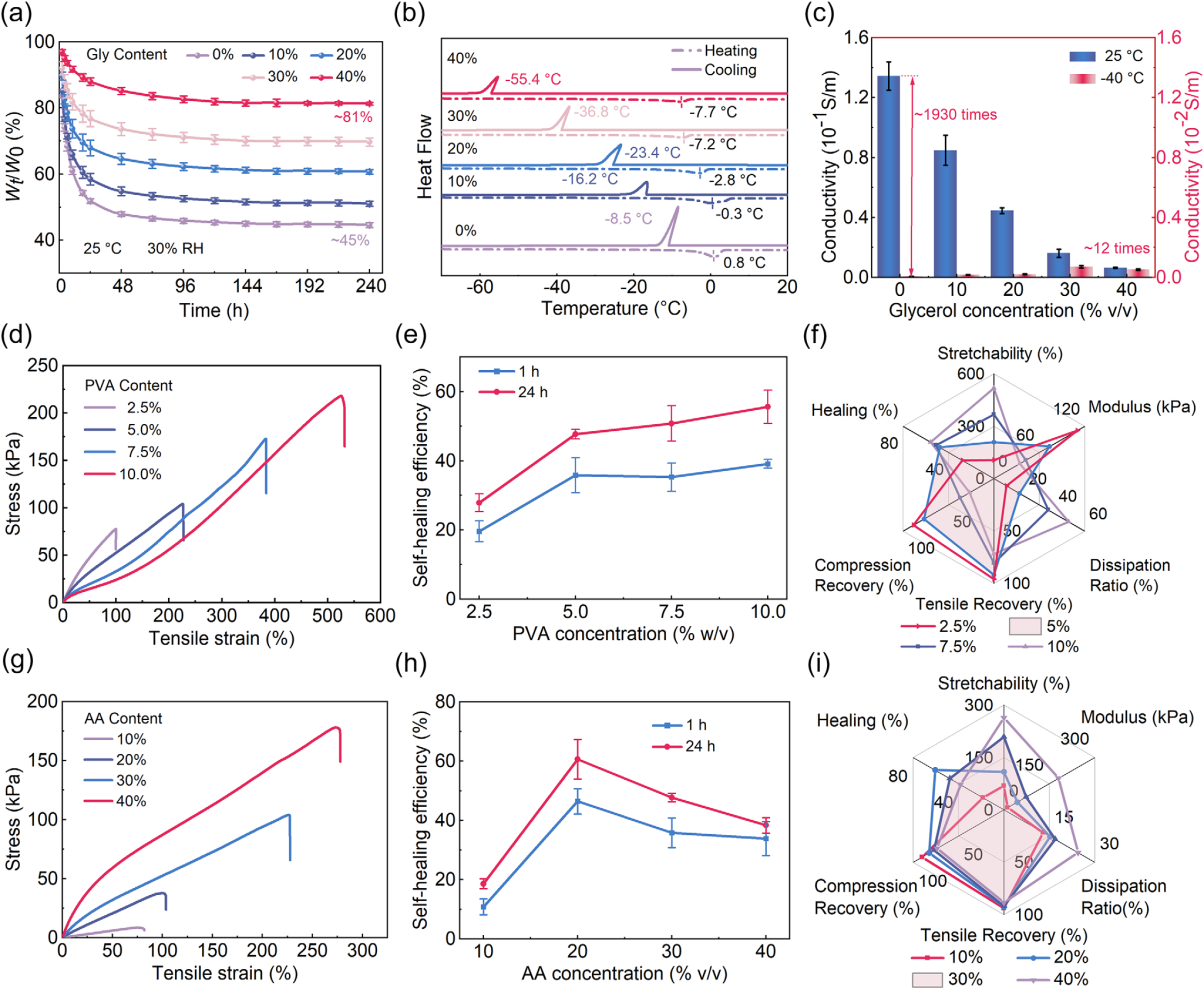
Optimization of component concentrations of GAPB‐Gly hydrogel. a) Water retaining ratio variations under different glycerol concentrations. b) DSC tests under different glycerol concentrations. c) Conductivity of GAPB‐Gly hydrogel with different glycerol concentrations. d) Tensile stress–strain curves and e) SHE of GAPB‐Gly hydrogel with different PVA concentrations. g) Tensile stress–strain curves, and h) SHEs under different AA concentrations. Comparison of the above properties of the hydrogel under different f) PVA and i) AA concentrations. Error bars were derived from five replicate measurements.

The multifunctionality of the GAPB‐Gly hydrogel could be finely tuned by optimizing the component concentrations of the introduced PVA, AA, glycerol, and borax. For this, the concentrations of GelMA and 2959 were fixed at 5% w/v and 1% w/v, respectively, to ensure sufficient mechanical strength while avoiding the risks of excessive rigidity or brittleness associated with higher concentrations [37]. In this content, we firstly investigated the effect of glycerol on the moisturizing and anti‐freezing properties by varying it from 0% to 40% v/v, while fixing the AA and PVA concentrations at 30% v/v and 5% w/v, respectively. Figure S7a shows the optical images of the GAPB hydrogel samples with glycerol concentrations of 0%, 10%, 20%, 30%, and 40% v/v after storage for 0, 2, 24, 72, and 240 h under 25°C and 30% relative humidity (RH). The sample without glycerol exhibited significant volume shrinkage after 24 h, and became dry and hardened after 240 h (10 d). In contrast, the volume shrinkage progressively decreased with the increasing glycerol concentration, and became negligible at 40% v/v glycerol even after 10 d. Figure 2a displays the mass variation of the 5 hydrogel samples with exposure time. Compared to the hydrogel without glycerol, the moisturizing rates (*w*_t/_ *w*_0_) of the GAPB‐Gly hydrogels showed remarkable increase, reaching as high as 98% after 24 h and 81% after 240 h at 40% v/v glycerol. These results well validated the effectiveness of the glycerol in hindering the water evaporation [38]. Figure S7b displays the morphological changes of the hydrogel samples during the thawing process at room temperature after freezing at ‐40°C for 24 h. The glycerol‐free hydrogel was completely frozen, and needed more than 10 min to restore its shape and flexibility. However, the ones with 10% and 20% v/v glycerol thawed after 2 and 6 min, respectively, demonstrating the enhanced freezing resistance. Especially, the hydrogel with 30% and 40% v/v glycerol maintained almost the same shape and softness as their original ones before freezing, indicating superior frost resistance. Differential scanning calorimetry (DSC, DSC200, Hitachi High‐Tech Analytical Science Co., Japan) analysis further revealed that the freezing point of the hydrogel dropped from ‐16.2°C to ‐55.4°C when the glycerol concentration increased from 10% to 40% v/v (Figure 2b). Figure 2c presents the tested conductivity of the 5 samples under 25°C and ‐40°C. The conductivity of the hydrogel without glycerol suffered an immense decrease (e.g., from 0.13 to 7.54×10^−5^ S m^−1^) when the temperature dropped from 25°C to ‐40°C. This was primarily due to the severely reduced ion migration caused by the crystallization of water molecules in the hydrogel under subzero temperatures. In comparison, the conductivity of the hydrogel with 40% v/v glycerol increased up to 5.70×10^−4^ S m^−1^ under ‐40°C, which was ≈7.6 times higher than that of the glycerol‐free one. This result highlighted the glycerol's critical role in preserving ion mobility under subzero temperature. In view of these, the 40% v/v glycerol was utilized for the GAPB‐Gly hydrogel to obtain optimal environmental stability and conductivity properties.

Further, the influences of the PVA, AA and borax on the mechanical and self‐healing properties of the GAPB‐Gly hydrogel were experimentally investigated by conducting strain–stress curve testing (Figure 2d‐i). Figure 2d shows the fracture strain and stress variations of the GAPB‐Gly hydrogels under different PVA concentrations, with a fixed AA concentration of 30% v/v. The borax content was set to one‐tenth of the PVA content to ensure stable mechanical strength of the hydrogel [16]. As shown in Figure 2d, the fracture strain increased nearly by 5‐fold (from 105.6 ± 11.1% to 518.2 ± 14.8%), and the tensile strength approximately tripled (from 69.8 ± 5.9 to 224.2 ± 5.8 kPa) as the PVA concentration was elevated from 2.5% to 10.0%. In contrast, the modulus decreased from 110.1 ± 7.0 to 34.3 ± 4.6 kPa within the PVA concentration variation range (Figure S8a). This was because that a higher PVA concentration could increase the density of H‐bonds and BEBs, which reformed the entanglement of GelMA chains and covalent network, and provided more sacrificial bonds for energy dissipation under external force, resulting in improved stretchability and tensile strength. The reduced tensile modulus stemmed from the relative lower increase rate of the fracture stress compared to the fracture strain. Cyclic tensile tests at 100% tensile strain revealed that the hysteresis increased with the PVA concentration (Figure S8b,c). The corresponding energy dissipation increased substantially from 8.3 ± 0.9% to 49.5 ± 3.2%, and the tensile elastic recovery ratio decreased from 96.0 ± 0.5% to 71.9 ± 1.9% as the PVA concentration increased from 2.5% to 10.0%. The decline in tensile elastic property could be attributed to the increase of the BEBs with the PVA concentration increasing, which reduced the chain segment flexibility, ultimately leading to diminished elastic properties. Figure S9 further elucidated the effect of PVA on compressive elastic property of the GAPB‐Gly hydrogel. As shown in Figure S9a, the compressive modulus gradually decreased as the PVA content increased. Specifically, when the PVA concentration rose from 2.5% w/v to 10.0% w/v, the compressive modulus declined from 248.0 ± 22.2 to 71.9 ± 15.6 kPa, exhibiting a trend consistent with tensile performance. Figure S9b,c displays the cyclic compression tests of GAPB‐Gly hydrogel under 50% strain. The energy dissipation substantially increased from 20.8 ± 3.0% to 74.6 ± 4.6%, and the compressive elastic recovery ratio reduced from 88.8 ± 1.7% to 26.8 ± 2.6% as the PVA concentration increased from 2.5% to 10.0%. These results indicated that adjusting the PVA concentration could tune the stretchability, tensile strength and Young's modulus of the GAPB‐Gly hydrogel within a large range. Further, we evaluated the influence of PVA on the SHE by testing the tensile stress–strain curves of the GAPB‐Gly hydrogels before and after repair. As shown in Figure S10b, after 24 h healing, the hydrogels with 2.5%, 5.0%, 7.5%, and 10% w/v PVA achieved maximum fracture strains of 32.1%, 102.9%, 205.3%, and 313.1%, respectively, demonstrating corresponding SHEs of 30.5%, 49.1%, 55.9%, and 60.4% (Figure 2e). The SHEs of the hydrogels were significantly improved compared to those after 1 h of healing (Figure S10a). The enhanced SHE stemmed from that the increased PVA concentrations contributed to form more reversible H‐bonds and borate ester crosslinks at the damaged interfaces. Meanwhile, the increased dynamic crosslinks could reduce the participation of polymer chains in covalent crosslinking, potentially lowering the overall density of the permanent network [39]. Thus, while dynamic reversibility was enhanced, the covalent crosslinking efficiency might be reduced, resulting in a softer yet more reconfigurable hydrogel. To make a trade‐off between the mechanical and self‐healing properties, 5% w/v PVA was selected for the GAPB‐Gly hydrogels (Figure 2f).

Consequently, the influences of AA on the mechanical and self‐healable properties of GAPB‐Gly hydrogels were examined, which determined the density of H‐bonds and reversibility of BEBs in the hydrogel network. we first explored the relationship of AA concentration with the GAPB‐Gly mechanical property by fixing PVA at 5% w/v. As shown in Figures 2g and S11a, with raising AA from 10% to 40% v/v, quadrupled fracture strain (from 68.7 ± 12.4% to 263.5 ± 8.9%) and boosted tensile modulus (from 8.9 to 183.5 kPa) were obtained. The corresponding energy dissipation rate moderately increased from 15.3 ± 2.2% to 24.5 ± 2.5%, and the elastic recovery rate decreased slightly from 91.9 ± 0.8% to 88.6 ± 1.5% (Figure S11b,c). The effective energy dissipation and excellent elastic recoverability presented at 40% v/v AA, could be attributed to that the enhanced H‐bond interactions enabled the hydrogel to dissipate more energy during deformation and thus improved its stretchability. Meanwhile, the increased polymer chain entanglement and crosslinking density could contribute to a more compact internal network, leading to significant improvements in tensile modulus. The dual role of AA also contributed to the enhancement of the energy dissipation and compressive modulus of the proposed GAPB‐Gly hydrogel in the 100% compressive tests (Figure S12). Figures 2h and S10c, d show the tensile curves of the GAPB‐Gly hydrogels under different AA concentrations after healing for 1 and 24 h. It could be observed that the SHEs increased with the extended healing time. The maximum fracture strains of the hydrogels with 10%, 20%, 30%, and 40% v/v AA were 15.4%, 67.9%, 102.9%, and 107.8%, and the corresponding SHEs in tensile strain were 19.7%, 67.2%, 49.1%, and 40.9% after 24 h healing, respectively. The SHE increased to its maximum value as the AA concentration was up to 20% v/v, and declined after that. The underlying reason lay in the multifaceted role of AA, i.e., regulating the network dynamics through initiating covalent crosslinking, forming dynamic H‐bonds and modulating reversibility of BEBs through influencing the microenvironmental pH. Specifically, within an optimal range, increased AA content could introduce more carboxyl groups to form dynamic H‐bonds while preserving polymer mobility, facilitating autonomous self‐healing. Moreover, AA‐induced slight pH decrease could shift BEBs toward a more reversible coordination state, promoting effective reformation of BEBs after damage. However, excessive AA can cause over‐polymerization and reduced chain mobility, significantly diminishing the hydrogel's self‐healing capacity. More importantly, over‐acidification caused by excessive AA could destabilize BEBs, preventing their reformation after mechanical damage. Therefore, precisely tuning the AA concentration was essential in maximizing the mechanical and self‐healing properties of the hydrogel. Thus, 30% v/v AA was chosen to balance the elasticity, stretchability, and self‐healing of the GAPB‐Gly hydrogel (Figure 2i). With these optimizations, the formulation GA_30_P_5_B‐Gly_40_ (40% v/v glycerol, 5% w/v PVA, 30% v/v AA) could be an elegant balance of cryogenic resilience, mechanical robustness, stretchability, and autonomous self‐repair, making it as a promising platform for healthcare technologies in extreme environments.

### 2.2 Mechanical Performance of Optimized GAPB‐Gly Hydrogel

**FIGURE 3:**
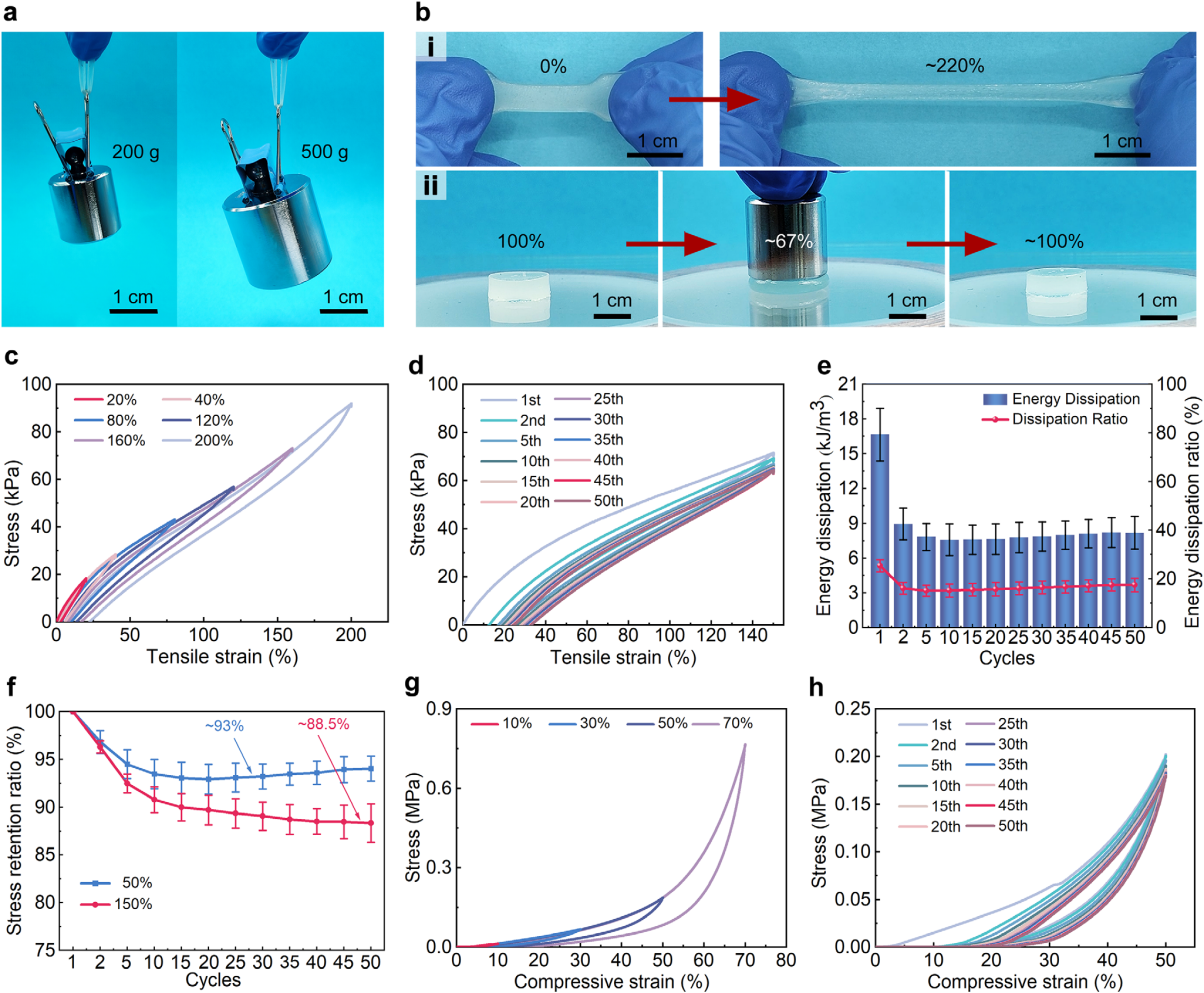
Mechanical properties of the optimized GAPB‐Gly hydrogel. Optical photos of a) weight‐bearing and b) stretching–compressing of the hydrogel. c) Tensile loading–unloading curves. d) Tensile stress–strain curves under consecutive cycles. e) Energy dissipation and dissipation ratio of the hydrogel. f) Variation of tensile stress retention ratio with loading‐unloading cycles. g) Compressive strain–stress curves under different strains. h) Compressive stress–strain curves under consecutive loading cycles. Error bars are derived from five replicate measurements.

Figure 3 presents the mechanical properties of the optimal GAPB‐Gly hydrogel. Owing to the introduced BEBs and covalent bonds, the dumbbell‐shaped hydrogel sample could withstand a maximum weight of 500 g (Figure 3a) and ≈220% tensile strain (Figure 3b‐i), which was 5.5 times higher than those of the pure GelMA hydrogel [40]. Such exceptional stretchability preserves structural integrity and electrical continuity under large deformations, ensuring stable and sensitive strain sensing performance [10]. Besides, the compression testing revealed that the hydrogel could completely recover its original shape after compressing to 67% (Figure 3b ‐ii), demonstrating excellent mechanical elasticity. Additionally, scanning electron microscope (SEM) characterization was conducted to examine the surface morphology of the hydrogel in both its initial state and under 100% tensile strain (Figure S13). In the unstretched state, the surface of GAPB‐Gly hydrogel exhibited uniformly oriented fiber‐like structures, while under 100% strain, a crosslinked mesh‐like architecture appeared in both longitudinal and transverse directions, indicating a homogeneous network structure within the hydrogel. Figure 3c–h further presents the quantitatively characterized mechanical properties of the optimal ones. Figure 3c shows the stress–strain curves obtained through cyclic loading–unloading at strains from 20% to 200%. The hysteresis loop area expanded slightly and dissipated energy increased from 0.6 to 16.2 kJ m^−3^, indicating that the H‐bonds and BEBs could effectively dissipate the energy generated during loading and unloading processes [41]. For all the tests, the elastic recovery rate remained above 85%. Further, fatigue resistance was tested with 50 continuous loading‐unloading cycles at 50% and 150% strains. The dissipated energy decreased from 1.96 ± 0.11 to 1.17 ± 0.04 kJ m^−3^ after 50 cycles under 50% strain, showing minimal variation (Figure S14). Similarly, at 150% strain, the energy dissipation in the first cycle was 16.66 ± 2.27 kJ m^−3^, which suffered a distinct decrease in the second circle and then maintained near‐overlapping hysteresis curves for all the residual loading–unloading cycles (Figure 3d). Consistent with this trend, Figure 3e illustrates that the energy dissipation ratio stabilized below 20% after 50 consecutive cycles. These results indicated that although the initial cleavage of covalent crosslinks induced energy dissipation, yet the balance of bond breakage and re‐esterification of BEBs system after the initial irreversible bond consumption led to stable hysteresis. Notably, the proposed GAPB‐Gly hydrogel showed a 88.5% stress retention at 150% strain after 50 repeated tensile–relaxation cycles, demonstrating superior fatigue resistance (Figure 3f) [42, 43].

Figure 3g further presents the loading‐unloading curves under compression strains of 10% to 70%. The mechanical performance of the GAPB‐Gly hydrogel was nearly overlapping paths. This remarkable performance originated from the reversible deformation of the densely crosslinked polymeric network, whose intricate chain architecture enabled stimulus‐responsive energy dissipation and structural restoration. A cyclic compression testing revealed distinct energy dissipation in the first loading‐unloading cycle, after which the hydrogel maintained the energy dissipation ratio at almost a constant value of 26% from the second to the 50th cycle. Additionally, it retained a high stress retention ratio of 89.5% after 50 cyclic loading–unloading (Figure 3h and Figure S15), significantly surpassing than that of pure GelMA gel [42]. This behavior arose from the multiscale crosslinked topology formed by the interpenetration of PVA, PAA, and GelMA, in which the dynamic physical and ionic interactions effectively suppressed crack propagation and limited excessive deformation of the GelMA matrix. The optimized system achieved a tensile modulus of 34.3 kPa, a compressive modulus of 71.9 kPa, a stretchability of 220%, and a remarkable tensile strength of 183.5 kPa, fulfilling its potential for applications in flexible tactile electronics.

### 2.3 Self‐Healing, Moisturizing, Cryotolerant, and Biocompatibility Properties

**FIGURE 4:**
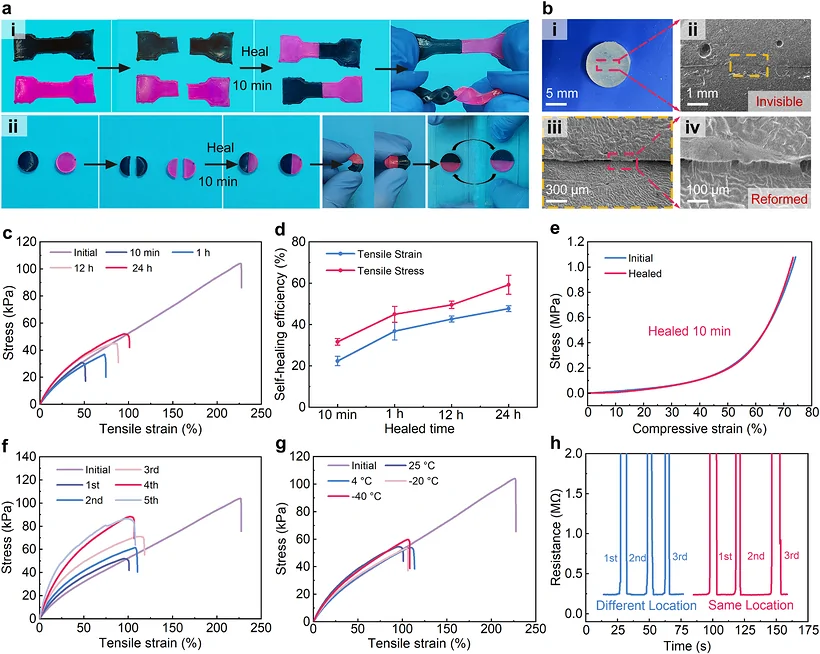
Self‐healing properties of the GAPB‐Gly hydrogel. a) Optical photographs of the healing process of the hydrogel. b) Optical photograph and SEM images of the GAPB‐Gly hydrogel healed surface. c) Tensile stress–strain curves of the hydrogel at varying healing times. d) Tensile strain and stress SHE of the hydrogel at varying healing times. Error bars are derived from five replicate measurements. e) Compression stress–strain curves of the initial and healing hydrogel. f) Tensile stress–strain curves of the hydrogel for five cutting/healing cycles. g) Tensile stress–strain curves of the hydrogel at varying healing temperatures. h) Resistance change of the hydrogel after three times cutting/healing.

As mentioned above, the combination of the introduced multiple H‐bonds and BEBs imparted the GAPB‐Gly hydrogel with crosslinking reconstruction capability after mechanical damage. To evaluate its self‐healing properties, two dumbbell‐ and two cylinder‐shaped samples with different colors were prepared and vertically bisected. Then the two pieces were brought together and kept in contact for 10 min at room temperature (Figure 4a). As shown in Figure 4a‐i, the two colored dumbbell‐shaped specimens seamlessly connected together without visible interfacial gaps, and the reconnected dumbbell could withstand a certain range of stretching and twisting. Similarly, the reformed cylinder hydrogel endured the bending in two mutually perpendicular directions without fracture at the reconnected surfaces (Figure 4a‐ii). These results demonstrated network re‐crosslinking at the cut surfaces of the two samples. SEM images after 24 h of healing exhibited nearly indistinguishable healing interfaces, further validating the reconstruction of chemical bonding and thus the polymer networks (Figure 4b).

Subsequently, a quantitative study on the effect of the healing time on tensile SHE was conducted at 25°C and 30% RH (Figure 4c,d). GAPB‐Gly hydrogel exhibited a tensile stress of 31.6 ± 1.6 kPa and a corresponding SHE of 31.2 ± 2.1% in fracture stress after 10 min of healing, which increased to 51.8 ± 2.7 kPa and 60.8 ± 5.5% after a 24 h healing, respectively. The approximately twofold fracture stress improvement in SHE was due to increased healing time, which contributed to sufficient molecular diffusion and reformation of more H‐bonds and BEBs at the fractured interface. Moreover, compressive properties of both the original and self‐healed hydrogel were tested after 10 min of healing (Figure 4e). The compressive stress–strain curve of the healed sample closely aligned with that of the original one, demonstrating rapid recovery capability. To address the potential failure threats in wearable electronics due to damage under various environments, self‐healing hydrogels should have self‐repairing capability under multiple damage and extreme environment conditions. Therefore, we further investigated the tensile property of the GAPB‐Gly hydrogel after multiple fracture‐healing cycles at the same location. In each test, the fractured hydrogel was kept under 25°C and 30% RH for 24 h. As shown in Figure 4f, the healed hydrogel sample suffered a distinct drop in the strain range compared to the original one, however, it still kept more than 100% fracture strain even after the fifth cutting‐healing cycles. Furthermore, the elastic modulus of the healed samples showed a slight increase after repeated healing cycles, likely due to continuous cross‐linking and reorganization of the polymer network. Further, to verify its feasibility of working in a cold environment, the self‐healing performance of the GAPB‐Gly hydrogel was assessed under different temperatures (Figure 4g). For this, five hydrogel samples of the same size were bisected and held together for 24 h at 25°C, 4°C, ‐20°C, and ‐40°C to evaluate their self‐healing performance across typical, physiological, and extreme subzero conditions. As shown in Figure 4g, the stress–strain curves of the healed hydrogels under different temperature conditions almost overlapped, and all samples maintained the tensile strains more than 100%. This remarkable SHE under subzero temperatures could be attributed to the binary glycerol‐water solvent which effectively lowered the freezing point of the hydrogel, and maintained the polymer chain mobility and reformation capability of crosslinks. Furthermore, the self‐healing capability of the dumbbell shaped hydrogel conductivity was evaluated: one was cut multiple times at the same location, while the other was cut at different positions. As shown in Figure 4h, for both cases, the resistances exhibited sharp increases once the samples were cut, and decreased rapidly to its original value in 5 seconds after the cut pieces were put into contact. Moreover, the resistance remained almost unchanged after three cutting‐healing cycles for both samples. This was because the conductive pathways primarily relied on dynamic interactions such as Van der Waals forces, H‐bonds, and BEBs within the polymer matrix, allowing rapid network reformation after rupture. Such dynamic reversibility enabled nearly complete reconstruction of conductive pathways with minimal resistance change over multiple break/heal cycles. These results validated the robust self‐healing property of our developed GAPB‐Gly hydrogel in mechanical and electrical properties, even across different temperatures.

**FIGURE 5:**
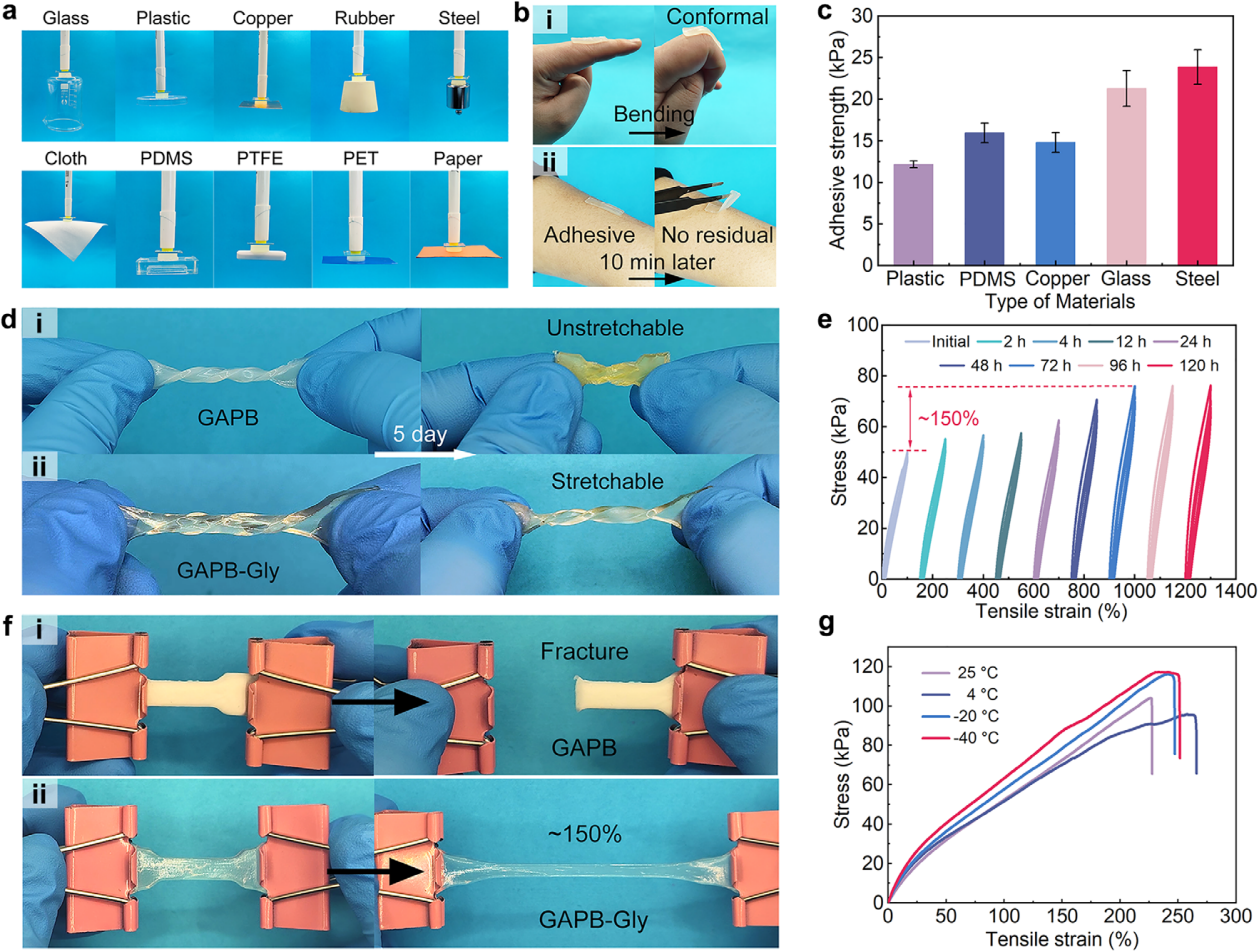
Adhesion, moisturizing, and anti‐freezing properties of the optimized GAPB‐Gly hydrogel. a) Adhesion performances to different substrates. b) Adhesions to finger and skin. c) Adhesion strength on various substrates. Error bars are derived from five replicate measurements. d) Stretchability comparison of the i) GAPB hydrogel and ii) GAPB‐Gly hydrogel before and after being stored at 25°C, 30% RH for 5 d. e) Tensile stress–strain curves of the GAPB‐Gly hydrogel over 5 d at 25°C, 30% RH. f) Stretchability of i) GAPB hydrogel and ii) GAPB‐Gly hydrogels frozen at ‐20°C for 24 h. g) Tensile stress–strain curves of GAPB‐Gly hydrogel frozen for 24 h under different temperatures.

Hydrogel adhesion is essential for stable, long‐term wearable monitoring. First, it enables secure attachment to skin or tissue, allowing accurate and reliable physiological signal acquisition. Second, it enhances structural robustness in multilayer flexible sensors by preventing delamination, slippage, and misalignment during bending and twisting. GAPB‐Gly hydrogel with diverse functional groups made it possible to form robust interfacial bonding, thereby ensuring stable interfacial adhesion. To confirm this, we assessed the adhesion property of the optimized GAPB‐Gly hydrogel by attaching it to a broad range of substrates—including glass, plastic, PDMS, copper, steel, cloth, polyethylene terephthalate (PET), paper, rubber, and polytetrafluoroethylene (PTFE) (Figure 5a). The GAPB‐Gly thin film could firmly adhere to all these materials substrates during lifting. Specifically, it could withstand the 150 g glass beaker and 100 g steel. The excellent adhesive performance of the GAPB‐Gly hydrogel across diverse substrates can be attributed to its multiple functional groups, including –NH_2_ from GelMA, –OH from PVA and glycerol, and –COOH from both AA and GelMA. These dynamic moieties enabled strong interfacial adhesion via H‐bonding and metal coordination. This multifunctional molecular “toolkit” overcame material‐specific limitations and achieved tough adhesion across a diversity of material interfaces (Figure S2). Furthermore, the adhesion performance of the GAPB‐Gly hydrogel was evaluated by attaching a thin GAPB‐Gly strip to human skin (Figure 5b). No relative sliding was observed during the repetitive finger bending process, and no residue was found when peeling off from the human arm after 10 min of attachment. When attached to skin surface, weak interactions such as H‐bonding, Van der Waals forces, and electrostatic attractions rapidly formed, and implemented firm adhesion. Under external peeling force, the irreversible bonding and weak intermolecular forces at the interface could dissociate readily. As a result, the GAPB‐Gly sample could be completely removed from the skin, demonstrating excellent reversibility and user comfort.

The adhesion performance was further quantitatively characterized by conducting axial tensile adhesion tests (Figure S16). Figure 5c shows that the tested maximum adhesion strengths of the GAPB‐Gly hydrogel to the surfaces of plastic, PDMS, copper, glass, and steel, which were 12.1 ± 0.4, 15.9 ± 1.2, 14.7 ± 1.2, 21.3 ± 2.1, and 23.9 ± 2.0 kPa, respectively. These adhesion strengths fall within or even exceeded that of commercial medical tapes (≈20 kPa) [44]. Among all the tested substrates, the hydrogel exhibited the strongest adhesion to steel, which was primarily due to the formation of dynamic metal–ligand coordination complexes between its functional groups (e.g., carboxyl groups) and iron ions on the steel surface. These interactions involved multiple coordination sites and relatively high bond energies, enabling strong yet reversible binding. Importantly, cyclic attachment testing revealed that the GAPB‐Gly hydrogel maintained almost constant adhesion strength with these materials even after 10 attach‐peel cycles (Figure S17a). In particular, the adhesion strength to steel was kept as high as 23.5 kPa after the cyclic attachment. For wearable tactile sensors, copper foil is commonly used as electrodes or wires. In a 10‐cycle adhesion test to copper foil (Figure S17b), the adhesion/displacement curves almost overlapped with each other. This reproducible adhesion performance was because the fact that the hydrogel–substrate interactions were primarily governed by reversible non‐covalent forces, such as H‐bonding, electrostatic interactions, and metal–ligand coordination. These interactions rapidly dissociate during detachment without damaging the hydrogel network or deactivating surface functional groups. Thus, the hydrogel retained active binding sites after each detachment, allowing efficient reformation of interfacial interactions and consistent adhesion over multiple attach–detach cycles. These results confirmed the exceptional adhesion performance of the GAPB‐Gly hydrogel to a broad range of material substrates, meeting the requirements of skin‐wearable sensor and the interlayer bonding in wearable tactile sensor fabrication.

To evaluate its moisturizing performance, we examined both macroscopic morphological changes and mechanical properties of the GAPB‐Gly hydrogel before and after being exposed to the environment with a temperature of 25°C and 30% RH for 5 d. As shown in Figure S18, the GAPB‐Gly hydrogel stabilized at 87% of its original volume after 2 d, while the GAPB hydrogel stabilized at only 58% of its original volume after the same period. This phenomenon was attributed to the low volatility and strong hydrophilicity of glycerol, which could form stable H‐bonds with water molecules, significantly inhibiting the rate of water evaporation. In contrast, the GAPB hydrogel without glycerol were more prone to dehydration under ambient conditions, leading to the dramatic shrinkage of the network structure. This structural shrinkage severely compromised the material's mechanical integrity, as evidenced by tensile testing of standard dumbbell‐shaped specimens (Figure 5d). Specifically, GAPB‐Gly hydrogel retained 150% elongation after 5 d, whereas the GAPB hydrogel became brittle and lost its deformability due to progressive moisture loss. Compression tests on GAPB‐Gly and GAPB samples (Movie S1) showed similar results. The GAPB hydrogel lost compressibility, whereas the GAPB‐Gly hydrogel maintained remarkable elasticity under repeated compression. Moreover, changes in the mechanical performance of the GAPB‐Gly hydrogel during storage were quantitatively evaluated using standard dumbbell‐shaped samples subjected to 10 cycles of 100% strain. Figure 5e shows the tensile strength increased to 1.5 times after 3 d and subsequently remained stable after 5 d. This enhancement in tensile strength could be due to the enhanced H‐bonding due to partial water evaporation and the dual‐function role of glycerol.

To evaluate the anti‐freezing capability of GAPB‐Gly hydrogel, dumbbell‐shaped samples were stored at ‐20°C for 24 h. The glycerol‐free sample fully solidified at ‐20°C, becoming incapable of tensile deformation and losing transparency (Figure 5f‐i). Conversely, the GAPB‐Gly hydrogel showed no notable morphological changes and maintained its stretchability (Figure 5f‐ii). Furthermore, the hydrogels were stored at 4°C, ‐20°C, and ‐40°C for 24 h, and their stress–strain curves showed that all these samples almost maintained consistent mechanical properties under different conditions (Figure 5g). Notably, even after 24 h of freezing at ‐40°C, the hydrogel retained its exceptional stretchability, with tensile strain surpassing 220%. This low‐temperature resilience stemmed from the H‐bonds formed with free water molecules and hydrophilic groups in the polymer chains, which depressing the freezing point of the internal aqueous phase. This interaction effectively inhibited ice crystal formation, preserved the amorphous and flexible structure of the polymer network and thus enabled the GAPB‐Gly hydrogel to retain excellent mechanical compliance even under subzero conditions (−40°C).

Further, we conducted a systematic investigation on the potential chemical leakage, cytocompatibility and skin compatibility of the GAPB‐Gly hydrogel since biocompatibility is a prerequisite for wearable physiological monitoring. To evaluate the leakage potential of unreacted components, GAPB‐Gly hydrogel was immersed in deionized water and incubated at 25°C, with its supernatant periodically extracted for analysis. The pH of the supernatant was 5.9 after 24 h due to the initial release of trace unreacted AA monomers, and gradually neutralized to 6.8 after 5 d, which fall well within the natural tolerance range of human skin (Figure S19) [45]. Figure S20 reveals that the AA concentration in the hydrogel extract plummeted by 96.9% (compared with GAPB‐Gly precursor solution) to only 1.31 µg mL^−1^, which was a trace level order of magnitude below established cytotoxicity thresholds. This result validated that the majority of AA monomers were covalently locked into the polymer network during photoinitiated polymerization [46, 47]. In addition, the release of the borax crosslinker was investigated by analyzing the content of boron via inductively coupled plasma optical emission spectrometry (ICP ‐ OES). According to results in Figure S21 and Table S2, the GAPB‐Gly hydrogel showed a maximum of only 0.98 ± 0.06 mg L^−1^ of boron over the entire 24 h period. These results collectively confirmed that both AA and borax were effectively cross‐linked, with only negligible amounts of unreacted species released. Furthermore, the viability of fibroblasts (L929 cells) was analyzed after co‐culturing with different concentrations (1, 5, and 10 mg mL^−1^) of GAPB‐Gly hydrogel extracts for 3 d. As shown in Figure S22, the cell viabilities remained above 95% throughout the culture period, indicating the high cytocompatibility of the GAPB‐Gly hydrogel. Furthermore, we attached the GAPB‐Gly hydrogel to a volunteer arm for 24 h to evaluate skin irritation during long‐term wear. As it shown in Figure S23, no hydrogel residue was observed on the skin surface after removal, and no allergic reaction occurred. The skin naturally returned to its original state within 15 min, further demonstrating the non‐irritating nature of the hydrogel. These results demonstrated that the GAPB‐Gly hydrogel exhibited favorable biocompatibility and biosafety for wearable applications.

As demonstrated above, the GAPB‐Gly hydrogel exhibited excellent multifunctionality, i.e., 220% stretchability, 91.9% in elastic recovery rate, 81% water retaining ability, which significantly exceeded those of pure GelMA hydrogel [26, 40, 42]. Table S1 presents a detailed comparison of the main properties of the GAPB‐Gly hydrogel with currently advanced multifunctional hydrogels. It could be observed that the self‐healing ability, moisturizing, and anti‐freezing properties were comparable to those of the hydrogels in previous research work, while its comprehensive performances significantly surpassed them. These advantageous properties validated the effectiveness of the proposed molecular design strategy for the hydrogel. The superior multifunctionality in mechanical, self‐healing, moisturizing, and adhesion enabled the GAPB‐Gly hydrogel to be a promising bio‐gel for practical application in the field of soft bio‐patch, wearable electronics, and sensors.

### 2.4 GAPB‐Gly‐Based Omni‐Healable Ionotronic (OHI) Pressure Sensors

**FIGURE 6:**
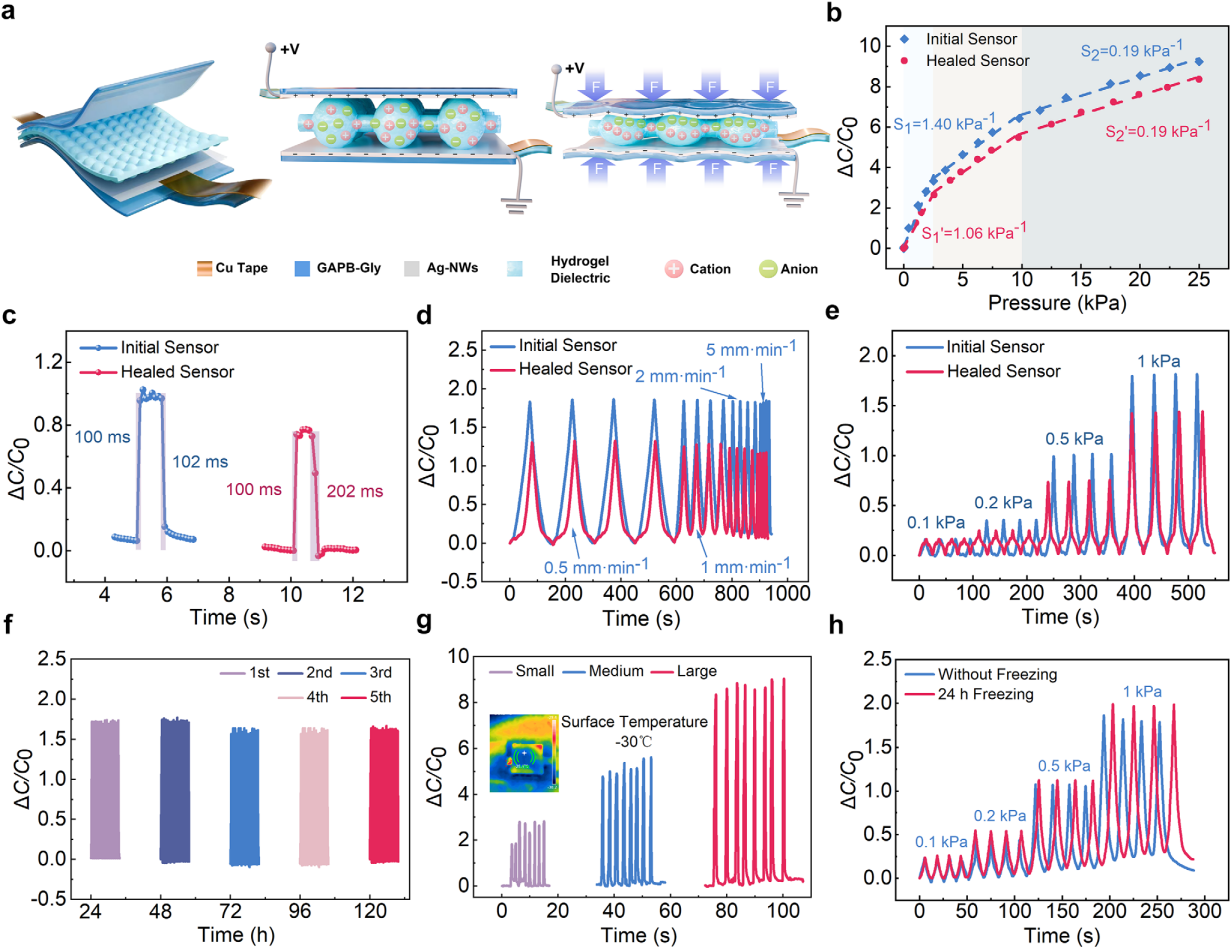
Basic performances of the OHI capacitive pressure sensor. a) Structural design and working principle. Key performance metrics of the initial and healed sensors including b) sensitivity, c) response and recovery times. Dynamic responses under different d) testing speeds and e) pressures. f) Response under 1 kPa pressure over 5 d. g) Response of the OHI pressure sensor under small (0.5 kPa), medium (3 kPa), and large (5 kPa) force amplitude under subzero temperature. h) Relative capacitance variation of the OHI sensor under different pressures before and after freezing.

Encouraged by superior multifunctionality of the proposed GAPB‐Gly hydrogel, an omni‐healable ionotronic (OHI) capacitive pressure sensor was devised, fabricated, and tested to validate its performance in practical applications. As depicted in Figure 6a, the OHI pressure sensor was composed of an intermediate GAPB‐Gly bi‐hemispherical dielectric layer sandwiched between two silver nanowires (Ag NWs) coated GAPB‐Gly thin film electrodes. Specifically, 200 mg of 10 wt% AgNWs solution was coated on GAPB‐Gly thin film surface to enhance the electrical conductivity. SEM images of the fabricated electrode showed that highly uniform and interconnected AgNWs conductive networks was successfully constructed on the film surface (Figure S27). NaCl was incorporated in the GAPB‐Gly dielectric layer to achieve a high concentration of positive and negative ion pairs. As such, electric double layers (EDL) were formed between the dielectric layer and electrode. Figure 6a and Note S1 illustrate the working mechanism of the OHI ionic pressure sensor. Upon applied pressure, the increased contact area induced Coulombic forces to separate ions within the dielectric layer, driving them toward oppositely charged electrodes. This nanoscale attraction generated a high capacitance through the EDL effect, which could significantly enhance the sensitivity of the OHI [48]. Meanwhile, the design of the two‐sided hemispherical microstructures was proposed to increase the compression of the GAPB‐Gly dielectric layer and thus the capacitance variation, further enhancing the pressure sensitivity. To implement the device fabrication, a complete solution‐processable fabrication technique was invented, which utilized the adhesion property to realize reliable bonding between the dielectric layer and electrode (Figures S24 and S25). Figure S26 depicts a digital picture of the final OHI pressure sensor. Figure S28 shows the optical and SEM images of the fabricated dielectric layer, which exhibited a uniform surface microstructure and excellent structure flexibility.

Before realistic application, the basic sensor performances were first characterized using the universal tensile tester. Figure 6b shows pressure sensitivity of the OHI pressure sensor reached 1.4 kPa^−^^1^ within 0–2.5 kPa, and dropped to 0.19 kPa^−1^ in a larger range of 10–25 kPa, which was due to the reduced compressibility of the dielectric layer under higher pressure [49]. To test its sensitivity after damage, the OHI pressure sensor were bisected and reconnected for self‐healing for 24 h. The healed OHI pressure sensor achieved a pressure sensitivity of 76% of that of the initial sensor in the range of 0–2.5 kPa, and nearly fully restored to its original level within 10–25 kPa. The excellent performance of the healed OHI pressure sensors was due to the mechanical and conductive restoring ability of both GNPB‐Gly hydrogel dielectric layer and AgNW‐coated electrode (Figure S29). The slight decrease in the pressure sensitivity may be due to misalignment at the fracture interface and the microscopic gaps between the electrode and dielectric layers after healing [50]. A rapid loading‐unloading process was conducted to evaluate the response speed. Figure 6c shows the response and recovery times of the original OHI pressure sensor were 100 ms and 102 ms, respectively, while the healed one suffered a longer recovery time (202 ms). This delay likely arose from localized crosslinking density gradients formed during healing, where the dynamic H‐bond network increased ion migration tortuosity and slowed the elastic recovery of the dielectric layer. To accurately determine the limit of detection (LOD) of OHI sensor, a glass slide weighting 140 mg was placed directly on its surface (≈3.5 Pa). As shown in Figure S30, both the initial and healed sensor exhibited a distinct and rapid response under 3.5 Pa, indicating that the OHI pressure sensor was capable of monitoring subtle physiological signals such as pulse and respiration. To evaluate its dynamic response performance, the pressure sensor was tested under loading/unloading speeds varying from 0.5 to 5 mm min^−1^ and pressure changing from 0.1 to 1 kPa, respectively. As shown in Figure 6d, both the initial and self‐healed sensors displayed rapid capacitance variation as the loading/unloading speeds varied from a low speed of 0.5 mm min^−1^ to a fast value of 5 mm min^−1^, demonstrating excellent dynamic performance. Meanwhile, both sensors also exhibited almost the same capacitance peaks and baseline under cyclic loading of pressure from 0.1 to 1 kPa, demonstrated excellent performance stability (Figure 6e). The slight attenuation in the capacitance changes of the healed sensor was primarily due to the incomplete recovery of the fractured crosslinking and thus the mechanical properties. To examine its mechanical durability, the sensor was cyclically compressed under 1 kPa at a loading speed of 2 mm min^−1^. As shown in Figure S31, the initial sensor maintained stable performance over 3000 cycles, while the self‐healed sensor remained reliable after 1200 cycles, with 5 times higher than previous GelMA‐based self‐healing hydrogel sensor [26]. Both the initial and healed sensors exhibited stable relative capacitance variations during cyclic loading, with only modest baseline drift and small changes in peak amplitude. The excellent structure robustness and durability could be attributed to the adhesion property of the GAPB‐Gly hydrogel. As shown in Figure S32, the maximum peeling force between the GAPB‐Gly dielectric layer and the electrode reached 1.30 N (Figure S32b). Notably, Figure S32c shows that the adhesion strength retained 83.7% of its initial one after 18 repeated peeling cycles (> 27.2 kPa at 15 cycles), and the peeling force remained more than ≈1.27 N over 5 d (Figure S32d). This robust adhesion is critical for maintaining sensor's robustness and durability.

Furthermore, the developed OHI sensor was experimentally tested over an extended period and at subzero temperature to evaluate its long‐term performance stability and working capability under harsh environment. Figure 6f presents the measured relatively capacitance variation of the OHI pressure sensor within a long‐time of 5 d. The capacitance response was tested under 20 loading‐unloading cycles every 24 h under 25°C and 30% RH ambient conditions. It could be observed that both the capacitance variation amplitudes and baseline nearly unchanged across tests, and the capacitive response presented negligible changes over 5 d (i.e., 120 h). Figures S33 and S34 further present the performance metrics variation during the long‐term usage, including the hydrogel mass loss, sensitivity retention, response/recovery time, baseline drift, and the signal‐to‐noise ratio (SNR). As shown in Figure S33, the OHI sensor retained 99.13% of its initial mass over 5 d. The high mass retention could be ascribed to the robust anti‐drying ability of the GAPB‐Gly hydrogel. Figure S34 shows that the sensor possessed a negligible sensitivity attenuation of ≈ 8.33% (from 0.204 to 0.187 kPa^−1^ in the range of 10–25 kPa), while maintaining consistently rapid response/recovery times of approximately 100 ms and a baseline drift below the order of 10^−4^ throughout the 5‐day period. Notably, the SNR measured on day 5 reached 35.34 dB, comparable to the average SNR reported by the Empatica E4 wristband [51]. This result further confirmed the sensor's capability for high‐fidelity and long‐term monitoring applications.

Figure 6g shows the experimentally tested performances of the OHI pressure sensors under ‐30°C, which was first stored at ‐40°C for 24 h and subsequently recovered at 25°C for 1 min. The sensor was able to output distinct and stable capacitance variation under cyclic finger touching, no matter under small force (≈0.5 kPa) or large force amplitude (≈5 kPa). Figure 6h further compares the sensing performances of the OHI pressure sensor with 24 h freezing and without freezing. The frozen sensors exhibited stable capacitance variation under cyclic pressure, and significant increase as the pressure varied from a tiny amplitude of 0.1 kPa to a large one of 1 kPa. Importantly, the capacitance variations of the frozen sensor under all the tested pressures are highly consistent with those of the sensors without freezing. To construct a realistic low temperature‐testing environment, we put the OHI pressure sensor inside a cold chamber and utilized infrared thermography to confirm its actual surface temperature (Figure S35a). As shown in Figure S35c, the device maintained a highly stable baseline and generated distinct capacitance response peaks under repeated stimuli of a standard wooden block (≈4 kPa) under ‐38.2°C. These results demonstrated that our developed OHI pressure sensor was able to operate under severe sub‐zero conditions. This superior working capability under subzero temperature could be attributed to the excellent anti‐freezing property of the GAPB‐Gly hydrogel. Table S3 summarizes the main performances of our OHI pressure sensor, and compared them with those of previously reported hydrogel sensors. It could be observed that the comprehensive performance of the OHI pressure significantly surpassed those of previous ones, including the anti‐freezing, self‐healing, long‐term stability, cyclic durability, and sensitivity and detection range [52]. This sharp enhancement in functionality and performance significantly expanded the applications for hydrogel‐based tactile sensors in harsh environments.

### 2.5 Wearable Health Monitoring

**FIGURE 7:**
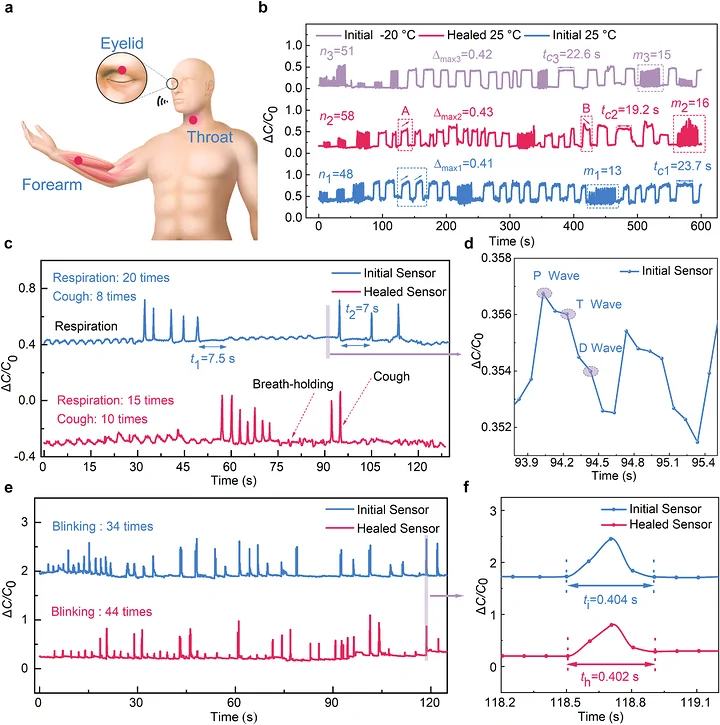
Application of the OHI pressure sensor in various physiological signals monitoring. a) Schematic diagram of physiological signal measurement. b) Signals from the initial, self‐healed, and frozen sensors when attached to the subject's forearm. c) Respiratory detection and d) carotid pulse signal detection when attached to the throat. e) Blinking signals detection, where f) presented the blinking interval data.

To verify its application in various physiological signals monitoring after mechanical damage and healing, and under low temperature, both original and healed OHI pressure sensor was attached to three typical parts of human body, i.e., the forearm, throat, and eyelid, to evaluate their sensing capability in tiny pressure, rapid signal acquisition and under subzero temperature (Figure 7a). Firstly, the pristine, self‐healed, and cryogenically treated OHI pressure sensors were attached to a volunteer forearm to evaluate the ability to monitor the muscle movement signals (Figure S36b). Figure S37 presents the response of the three sensors under different gripping intensities. All the sensors showed rapid increase in the output capacitance once the volunteer clenched his fist, and stable capacitance variation under the multiple repeat grips. Importantly, the self‐healed and ‐20°C treated OHI pressure sensors showed almost consistent capacitance variation with that of the initial one. Figure 7b further displays the experimental results of the three sensors in continuous 10 min monitoring of the different muscle movements, which involved the clench, maximum consecutive clenches, and maximum clenched‐state duration. Both the self‐healed and cryogenically treated OHI pressure sensors could effectively monitor aforementioned gripping activities, and output stable capacitance under more than 10 min of maximum clenched‐state duration. Importantly, the capacitance variations (0.43 and 0.42) of the self‐healed and frozen OHI pressure sensors under the maximum clenches were almost the same as that (0.41) of the initial one. These results demonstrated that our OHI pressure sensors possessed excellent performance even after damage or under cold environment.

Further, we also tested its sensing performances under other realistic wearable scenarios to validate the practical application ability, including various physiological signals monitoring, exercise‐related usage, and multi‐day usage on body or long‐term monitoring. Firstly, the OHI pressure sensor was attached to the volunteer's throat to monitor critical respiratory parameters, including the breathing, coughing, and carotid pulse signals (Figure S36c). Figure 7c shows the results of the respiratory and coughing measured by the pristine and self‐healed sensors. Both sensors could detect the gentle pressure variation caused by the breathing in a resting state, as well as the large ones by coughing during the 2 min of continuous measurement. Both capacitance variations of the healed sensors under breathing and coughing were well consistent with that of the pristine sensor. Additionally, the tiny pressure variation caused by carotid pulse could also be detected, which clearly exhibit the key features, such as the primary wave (P wave), tidal wave (T wave), and dicrotic wave (D wave), as shown in Figure 7d. These results demonstrated the wide range of pressure monitoring capability of the healed OHI sensor, i.e. from tiny pulse vibration, gentle breathing to significant coughing. Further, the OHI pressure was affixed to the eyelid of the volunteer subject to examine its capability in monitoring a spectrum of ocular motion monitoring scenarios (Figure S36d). As shown in Figure S38, the sensor exhibited rapid responses and accurately identified the signal peaks corresponding to different blink amplitudes. Figure 7e,f shows measured results of various eye blinking activities during a 2 min continuously monitoring. Both the pristine and self‐healed OHI sensors could effectively recognize each blinking, exhibiting almost the blink lasting time, i.e. 0.404 and 0.402 s, respectively. These results indicated that the healed sensors almost possess the same dynamic and pressure sensitivity as those of the pristine one.

Figure S39 presents the usage of our OHI sensor under sweating or exercise condition, which was assessed by conducting a 20 min continuous gripping with a force of 3–10 kg using a dynamometer. As shown in Figure S39, the sensor consistently captured distinct response peaks without noticeable signal degradation throughout the 5–20 min exercise period. Notably, it maintained a highly recognizable waveform for a 5 kg grip even on noticeably sweaty skin at the 20th min, in which its peak capacitance variation (3.32%) almost matched the initial dry‐skin baseline (3.18%). These results validated its performance robustness under sweating or exercise‐related usage condition.

Figure S40 presents the results of multiday usage capability on human body, which was tested by attaching our OHI sensor to a volunteer's throat to continuously monitor respiratory and coughing signals over a 5‐day period. As shown in Figure S40, the sensor could well monitor the respiratory and coughing even after 5 d, and showed almost the same capacitance variation as those tested at the first day. These results effectively validate the long‐term monitoring capability of our pressure sensors, which significantly surpassed those hydrogel pressure sensors reported previously [53]. Aforementioned results demonstrated the excellent performance robustness and multi‐functionalities of our developed OHI pressure sensor, which encourage its application in realistic wearable scenarios.

## 3 Conclusions

In summary, we have successfully developed a stretchable, self‐healable, and environmental‐stable GelMA hydrogel by incorporating PVA, AA, borax, and glycerol into GelMA. The excellent multifunctionality stemmed from the ingenious integration of multiple H‐bonds, covalent bonds, mildly acidic microenvironment‐tuned BEBs, and glycerol/H_2_O binary solvents. The multiple H‐bonds, strong covalent bonds, and finely tuned BEBs contributed to synergistic improvement of the mechanical elasticity, strength, and stretchability. Meanwhile, the dynamic reversible H‐bonds and BEBs concurrently endows the hydrogel with self‐healing capability. Furthermore, the strong hydration interactions between glycerol and water molecules in the solvent ensured the hydrogel's function under both dry and freezing conditions. Based on this unique design strategy, the GAPB‐Gly hydrogel achieved an optimal balance of multiple functionalities, demonstrating excellent stretchability (≈220%), impressive elasticity (elastic recovery rate of ≈91.9%), exceptional fatigue resistance (88.5% stress retention after 50 cycles at 150% strain), high SHE (up to 61%) even at low temperatures (‐40°C), excellent moisturizing (81% hydration after 10 d) and anti‐freezing properties (‐55.4°C), along with strong and repeatable adhesion (≥23.5 kPa, 10 cycles). The developed OHI pressure sensor demonstrated high sensitivity (1.4 kPa^−^^1^ in 0–2.5 kPa), rapid response/recovery times (100/102 ms), a low LOD (3.5 Pa), and impressive durability (over 3000 cycles), long‐term performance stability (up to 5 d) and low‐temperature function capability (‐30°C). The exceptional sensing and multifunctional properties of the sensor fulfill the environmental adaptability and long‐term monitoring requirements for muscle movement, throat activity, and eye movement signals in harsh environments, such as mechanical damage, long‐term, and sub‐zero temperature. This study provides a promising approach for the rational design of multifunctional and adaptable hydrogels, and promotes their practical application under harsh environment.

## 4 Experimental Section

### 4.1 Materials

Gelatin from porcine skin (Type A, gel strength ≈300 g Bloom), methacrylic anhydride (MA, 94%), Dulbecco's phosphate buffered saline (DPBS) and Irgacure 2959 photoinitiator (98%) were procured from Sigma Aldrich. Polyvinyl alcohol (PVA, 86.5‐89.0 mol% alcoholysis degree), sodium tetraborate (borax, ≥99%), sodium chloride (NaCl, 99.5%), methyl green (AR), and rhodamine B (AR) were acquired from Aladdin. Acrylic acid (AA, 98%) was procured from Meryer. Ag NWs suspension (10 mg mL^−1^, diameter ≈20 nm, length ≥30 nm) was acquired from Shanghai Ouyi organic photoelectric materials. All chemicals and reagents were utilized without further purification.

### 4.2 Preparation and Chemical Structure Characterization of GAPB‐Gly Hydrogels

GAPB‐Gly hydrogel was synthesized using a one‐pot method by UV‐initiated crosslinking. First of all, GelMA was synthesized according to a previously established method [30]. Subsequently, a mixture of 8 mL of glycerol and 6 mL of deionized (DI) water was prepared, followed by adding 1 g PVA to the binary solvent. The mixture was heated at 90°C for 3 h until the PVA was fully dissolved. Subsequently, 100 mg of borax, 6 mL of acrylic acid, 200 mg of photoinitiator, and 1 g of GelMA were added to the PVA solution, with continuous stirring until a homogeneous mixture was obtained. After degassing using an ultrasonic cleaner (HEAT US BATH 1.9L, Thermo Fisher, USA), the solution was poured into a PDMS mold and exposed to UV irradiation in a light‐curing box (BlueWave FX1250, Dymax, USA) at an intensity of 50 mW cm^−2^ for 5 min to form the GelMA/AA/PVA/Borax‐Glycerol (GAPB‐Gly) hydrogel.

For all prepared hydrogel, the GelMA concentration was fixed at 5% w/v, while the photoinitiator was kept at 1% w/v, and the mass ratio of PVA to borax maintained at 10:1 [54]. The hydrogels were defined as GA_x_P_y_B‐Gly_z_ based on the concentrations of AA, PVA, and glycerol in the system during components optimization. X represents the concentration of AA, which was varied at 10%, 20%, 30%, and 40% v/v; Y represents the concentration of PVA, which was varied at 2.5%, 5.0%, 7.5%, and 10.0% w/v; and *Z* represents the concentration of glycerol, which was changed at 0%, 10%, 20%, 30%, and 40% v/v.

The chemical structure and composition of the prepared hydrogels were characterized by NMR (Bruker 400M, Bruker BioSpin GmbH, Germany), FTIR (Bruker ALPHA II, Bruker Optik GmbH, Germany) and XPS (EscaLab 250Xi, Thermo Fisher Scientific Inc., USA). For NMR analysis, GelMA and gelatin were dissolved in deuterium oxide solution. For FTIR and XPS measurements, the hydrogels and their individual components were freeze‐dried for 72 h prior to analysis.

### 4.3 Characterization of Mechanical Properties

All the mechanical property tests were performed by using a universal tensile testing machine (SUST CMT1202, China). The measured sample was molded into a dumbbell shape with the size of 15 mm (L) × 7 mm (W) × 2 mm (H), and all the tensile and compression tests were conducted at a speed of 50 mm min^−1^ at 25°C [55]. The elastic modulus (*E*) was defined as the fit slope of the stress–strain curves in 0–20% strain. The toughness (*Γ*) was calculated by integrating the area of tensile stress–strain curves, as described in Equation (1):

(1) $$\Gamma =\int_{\varepsilon_{0}}^{\varepsilon_{f}}d\varepsilon$$

where *ε*_0_ and *ε*_f_ refer to the initial strain and fractured strain, respectively. The energy dissipation (Δ*U_i_*) was calculated by integrating the area between the loading and unloading stress–strain curves, which was given as Equation (2):

(2)

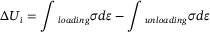

where σ refers to the stress. The energy dissipated ratio (*δ*) was calculated by Equation (3):

(3)

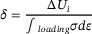




All tests were conducted at least five times for each group to reduce random errors.

### 4.4 Characterization of Self‐Healing Properties

The self‐healing properties were evaluated by cutting the hydrogel into two pieces, staining, reconnecting, and maintaining the samples at 25°C, 30% RH for 24 h, followed by qualitative and quantitative analysis. The morphology of the healed crack was characterized by using scanning electron microscopy (SEM, U‐8010, Hitachi High‐Tech Analytical Science Co., Japan) after freeze‐drying the samples for 24 h using a freeze‐dryer (SCIENTZ‐18ND/D, Ningbo Xinzhi Biotechnology Co., China).The self‐healing efficiency (SHE) of the GAPB‐Gly hydrogels was evaluated by comparing the tensile properties of the pristine and healed dumbbell‐shaped specimens under the identical parameters (including the testing apparatus, sample dimensions, and stretching speed) as detailed in the Mechanical Property Measurement section. The tensile strength at fracture was recorded for each sample. To systematically investigate the healing kinetics, hydrogels prepared with different ratios of PVA (2.5, 5, 7.5, and 10 wt%) and AA (10, 20, 30, and 40 v/v%) were tested at different healing intervals (1 h and 24 h). The SHE was quantitatively defined and calculated as the ratio of the tensile strength of the healed sample (*σ_healed_*) to that of the pristine sample (*σ_pristine_*), expressed as Equation (4):

(4) $$SHE\left(\%\right)=\sigma_{healed}/\sigma_{pristine}\times 100\%$$

### 4.5 Characterization of Adhesion, Anti‐Freezing, and Conductive Properties

The adhesion performances of the GAPB‐Gly hydrogel to various substrates were tested through the configuration shown in Figure S16. The cylindrical hydrogel samples with a diameter of 15 mm and a height of 2 mm were prepared for the test. The sample was fixed to the clamp using an adhesive tape, and then was pre‐compressed to the tested substrate for 2 min. The test was conducted by using a tensile tester (SUST CMT1202, China) under a stretching speed of 50 mm min^−1^.

The freezing points of the GAPB‐Gly hydrogels with varied glycerol contents were tested by using differential scanning calorimetry (DSC, DSC200, Hitachi High‐Tech Analytical Science Co., Japan), which was used to quantitatively assess their anti‐freezing properties. The conductivity of the hydrogels was tested using a handheld multimeter (VICTOR 17, Shenzhen Yisheng Victor Tech Co., China). Additionally, a precision LCR meter (E4980AL, Keysight, USA) was used to measure the resistance changes of the hydrogel before and after healing.

### 4.6 Quantitative Assessment of Hazardous Species Leakage

To thoroughly investigate the potential risks of hazardous substance leakage and evaluate the chemical stability of the hydrogel, chemical leaching and pH stability tests were systematically conducted. Specifically, GAPB‐Gly hydrogel (5.0 g) was immersed in DI water (50 mL) and incubated in a 25°C constant‐temperature incubator (JJF 1101, Lichen Scientific Group, China). At predetermined time intervals (0, 24, 48, 72, 96, 120 h), the sample was gently vortexed, and the supernatant was carefully extracted for measurement. The pH was recorded using a portable pH meter (PH818, Sigma Technology, China). To continuously drive the leaching process, the residual soaking liquid was entirely discarded after each measurement, replaced with 50 mL of fresh DI water, and returned to the incubator for the next cycle.

For the quantitative analysis of residual AA leakage, high‐performance liquid chromatography (HPLC) was employed to monitor the amount of AA in the supernatant. In brief, 5.0 g of polymerized GAPB‐Gly hydrogel in 50 mL DI water was incubated at 25°C with a shaking speed of 50 rpm (*n* = 5 per group). After 24 h, 1.5 mL of the supernatant was extracted, filtered through a 0.22 µm hydrophilic PTFE syringe filter (DL‐C‐SF, Beekman Biotechnology Co. Ltd., China) and transferred to a 2 mL amber vial for HPLC analysis. DI water and precursor sample (5.0 g of unpolymerized GAPB‐Gly precursor solution in 50 mL DI water) were used as blank and control.

To further quantify the potential release of borax crosslinker, ICP‐OES analysis was conducted using the same three experimental groups (*n* = 5 per group) incubated at 25°C. At specified intervals (1, 3, 6, 12, and 24 h), 5 mL of the supernatant was extracted, filtered through a 0.45 µm PES membrane into a 15 mL PP tube, and acidified to pH < 2 using 0.05 mL of guaranteed reagent grade nitric acid (65%). Similar to the pH test, the residual liquid was completely replaced with 50 mL of fresh DI water after collecting the supernatant at each time point. To prevent exceeding the calibration limit, the precursor samples were diluted 50‐fold with 5% nitric acid before analysis, whereas the blanks and the GAPB‐Gly extracts were analyzed after filtration through a 0.22 µm filter. The boron concentration was determined via an ICP‐OES system (iCAP 7000 Series, Thermo Fisher Scientific, USA).

### 4.7 Cytocompatibility Characterization

To evaluate the biocompatibility of the GAPB‐Gly hydrogels, in vitro cytocompatibility test was conducted using mouse fibroblast L929 cells (Procell Life Science & Technology Co., Ltd., China). Cell viability was quantitatively assessed via a Cell Counting Kit‐8 (CCK‐8, BS350A, Lanjie Ke Technology Co., Ltd., China) assay after co‐culturing the cells with hydrogel extracts. Briefly, L929 cells were seeded into 96‐well culture plates at a volume of 100 µL per well, and incubated in a humidified incubator (LC‐HN‐36BS, Lichen Scientific Group, China) at 37°C with 5% CO_2_ for 24 h. Subsequently, the original culture medium was discarded and replaced with 100 µL of culture medium containing GAPB‐Gly hydrogel extracts at varying concentrations (1, 5, and 10 mg mL^−1^). For comparison, the control group was treated with an equal volume of PBS (BL302A, Lanjie Ke Technology Co., Ltd., China), while a blank group containing only complete culture medium (without cells) was established to eliminate background interference. Following a 24 or 72 h of co‐incubation, cells were washed with PBS, and 10% of CCK‐8 in a serum‐free medium was added to each well, followed by 2 h of additional incubation. Finally, the absorbance of each well at wavelength of 450 nm was measured using a microplate reader (Varioskan ALF, Thermo Fisher Scientific, USA). The cell viability was calculated according to Equation (5):

(5) $$Cell\;viability\;\left(\%\right)=\left(A_{GAPB-Gly}-A_{Blank}\right)/\left(A_{PBS}-A_{Blank}\right)\times 100\%$$

where *A*_GAPB‐Gly_, *A*_PBS_, and *A*_Blank_ represent the absorbance values of the experimental groups (treated with hydrogel extracts), the control group (treated with PBS), and the blank group, respectively.

Furthermore, to evaluate the in vivo skin compatibility and practical wearability of the hydrogels, cylindrical GAPB‐Gly hydrogel samples (*D* = 15 mm, *H* = 10 mm) were prepared and directly attached to the human skin for 24 h. To ensure stable attachment, the hydrogels were securely fixed using a commercial breathable polyurethane (PU) tape (A706, Hons Medical Technology Co., Ltd, China). Simultaneously, a parallel control group was established by applying only the breathable PU tape to skin adjacent to the test site to observe any potential irritation originating from the adhesive tape itself. After 24 h, the tapes and hydrogel samples were carefully removed. The condition of the skin surface was photographically recorded immediately upon removal and 15 min post‐removal to comprehensively assess the biosafety and non‐irritating nature of the hydrogel.

### 4.8 Omni‐Healable Ionotronic (OHI) Pressure Sensor Assembly Process

The detailed fabrication process of Ag NWs/GAPB‐Gly composite electrodes and the two‐sided hemispherical dielectric layer was described in Figures S24 and S25, respectively. Specifically, conductive copper tape (3M1181, 3M, USA) was adhered to the electrode protrusions, and the sensor was assembled into an electrode‐dielectric‐electrode sandwich structure by aligning the electrodes’ conductive silver paste surface with the dielectric layer's hemispherical structure surface. The fabricated omni‐healable ionotronic (OHI) pressure sensor had an area of 2×2 cm^2^ and an overall thickness of 4 mm, with the electrodes of ≈1 mm and a dielectric layer thickness of 2 mm.

### 4.9 Performance Characterization of the OHI Pressure Sensor

The basic performance of the sensor was measured using a precision LCR meter. The testing system for the OHI pressure sensor was depicted in Figure S36. After connecting the sensor to the LCR meter, the relative capacitance changes under varying pressure were tested at 1 kHz with a 1 V AC signal. The sensor detected external pressure changes based on the electric double layer (EDL) principle, as illustrated in Figure 6a. The sensitivity was calculated as Equation (6):

(6) $$S=\frac{\Delta C/C_{0}}{\Delta P}$$

where Δ*C* represents the capacitance change, *C*_0_ is the initial capacitance value, and Δ*P* denotes the pressure variation [56]. To ensure the uniform pressure distribution on the sensor surface, a cover glass with an area of 2×2 cm^2^ and a mass of 140 mg was placed on the sensor during testing.

The interfacial bonding strength of the OHI pressure sensor was characterized with a 90° peeling test using a universal tensile testing machine (SUST CMT1202, China). The assembled sensor was fixed to a rigid substrate using double‐sided tape, while the unbonded top layer was clamped to the upper grip. The peeling process was performed at a constant speed of 50 mm min^−1^ at 25°C. The maximum adhesion force was defined as the interlayer bonding strength.

Post‐freezing recovery and in situ cryogenic operation test were conducted to comprehensively assess the sensor's performance under extreme cold conditions. For the post‐freezing recovery test, the OHI sensor was initially stored at ‐40°C for 24 h in an ultra‐low‐temperature medical freezer (DW‐86L100J, Haier Biomedical, China). Subsequently, the sensor was exposed to a room‐temperature (25°C) to thaw for 1 min. Prior to the test, the actual surface temperature of the OHI sensor was measured using an infrared thermal imager (UTi320E, Uni‐Trend Technology, China). Immediately following, dynamic output electrical signals of the sensor were recorded under both gentle finger pressing and a gradient pressure increasing from 0.1 to 1 kPa applied by the universal tensile testing machine.

For in situ cryogenic operation test, the OHI sensor was first stored at ‐40°C for 24 h in the ultra‐low‐temperature medical freezer. In order to strictly maintain this controlled low‐temperature environment, the sensor was directly connected to the LCR meter. Repeated compressive stimuli were applied using a standard wooden block (25 × 25 × 25 mm^3^, 10 g), and the real‐time capacitance variations were continuously recorded via a portable laptop (Yaoshi 16, Mechrevo, China).

The signal quality of the OHI pressure sensor on the 5th day was evaluated by analyzing the signal‐to‐noise ratio (SNR) according to Equation (7):

(7) $$\text{SNR}=20\log_{10}\left(\Delta S/\sigma \right)$$

where *ΔS* represents the effective response signal variation (i.e., the capacitance change) under external pressure, and *σ* denotes the standard deviation of the baseline background noise [57].

### 4.10 Statistical Analysis

Statistical analyses were performed using Origin 2021 (OriginLab Corporation, Northampton, MA, USA). All experiments were repeated at least five times and the experimental data were presented as the mean ± standard deviation (SD).

## Author Contributions

**Zhikang Li**: supervision, conceptualization, Writing – review and editing, funding acquisition. **Gengyu Han**: writing – review and editing. **Boqing Jia**: software, formal analysis, writing – original draft, methodology. **Jijian Lu**: conceptualization, methodology, validation, data curation, writing – original draft. **Changxu Zhang**: validation, methodology, formal analysis. **Bin Wang**: investigation. **Jiaxiang Wang**: investigation. **Kang Zhao**: investigation. **Muhammad Afzal Khan Qureshi**: investigation. **Lan Yu**: investigation, validation. **Min Li**: writing – review and editing, funding acquisition, validation. **Guoxi Luo**: writing – review and editing, funding acquisition. **Tong Wang**: investigation. **Qijing Lin**: funding acquisition, writing – review and editing, visualization. **Weishi Li**: writing – review and editing, supervision. **Libo Zhao**: supervision, funding acquisition, writing – review and editing, project administration, resources. **Yumeng Xue**:t writing – review and editing, funding acquisition, project administration, supervision, resources, investigation.

## Ethics Statement

The human subject experiments were conducted in compliance with the protocols approved by Peking University Third Hospital Medical Science Research Ethics Committee (IRB00006761‐M2024893). All subjects gave written informed consent before participation in the study. For all demonstrations on human skin, signed consent was obtained from the volunteer.

## Conflicts of Interest

The authors declare no conflicts of interest.

## Supporting information




**Supporting File 1**: advs77587‐sup‐0001‐S1.mp4.

**Supporting File 2**: advs77587‐sup‐0002‐SuppMat.pdf.

## Data Availability

The data that support the findings of this study is available from the corresponding author upon reasonable request.
