# Supplementary material for: Mechanically Robust, Self‐Healable, and Cryotolerant GelMA Hydrogel for Wearable Tactile Sensors with Enhanced Durability, Lifetime, and Environmental Stability
Source: Adv Sci (Weinh). 2026 Sep 16:e77587. Online ahead of print. doi: 10.1002/advs.77587 (PMC13583233; doi:10.1002/advs.77587)
Supplement: Supplementary file 1 [file ADVS-9999-e77587-s001.pdf]

Supporting Information

Mechanically Robust, Self-healable, and Cryotolerant GelMA Hydrogel for Wearable Tactile Sensors with Enhanced Durability, Lifetime and Environmental-stability

Zhikang Li[1,2,4], *Gengyu Han*[5,6,7,#], *Boqing Jia*[1,2,3,#], *Jijian Lu*[1,2,3], *Changxu Zhang*[1,2,3], *Bin Wang*[1,2,3], *Jiaxiang Wang*[1,2,3], *Kang Zhao*[1,2,3], *Muhammad Afzal Khan Qureshi*[1,2,3], *Lan Yu*[1,2], *Min Lin*[1,2,4], *Guoxi Luo*[1,2,4], *Tong Wang*[1,2,3], *Qijing Lin*[1,2,4] *Weishi Li*[5,6,7,*],*Libo Zhao*[1,2,4,*], *Yumeng Xue*[8,*]

[1] School of Instrument Science and Technology, Xi'an Jiaotong University, Xi'an 710049, China

[2] State Key Laboratory for Manufacturing Systems Engineering, State Industry-Education Integration Center for Medical Innovations, International Joint Laboratory for Micro/Nano Manufacturing and Measurement Technologies, Shaanxi Innovation Center for Special Sensing and Testing Technology in Extreme Environments, Shaanxi Provincial University Engineering Research Center for Micro/Nano Acoustic Devices and Intelligent Systems, Xi'an Jiaotong University, Xi'an 710049, China

[3] School of Mechanical Engineering, Xi'an Jiaotong University, Xi'an 710049, China

[4] Shandong Laboratory of Advanced Materials and Green Manufacturing at Yantai, Yantai 264000, China.

[5] Department of Orthopaedics, Peking University Third Hospital, Beijing 100191, China

[6] Engineering Research Center of Bone and Joint Precision Medicine, Peking University Third Hospital, Beijing 100191, China

[7] Beijing Key Laboratory of Spinal Disease Research, Peking University Third Hospital, Beijing 100191, China

[8] State Key Laboratory of Solidification Processing, School of Materials Science and Engineering, Northwestern Polytechnical University and Shaanxi Joint Laboratory of

Graphene, Xi'an 710072, China

[#] Zhikang Li, Gengyu Han and Boqing Jia contributed equally to this work.

Email Address: puh3liweishi@163.com

Email Address: libozhao@ xjtu.edu.cn

Email Address: ymxue@nwpu.edu.cn

Supplementary Figures

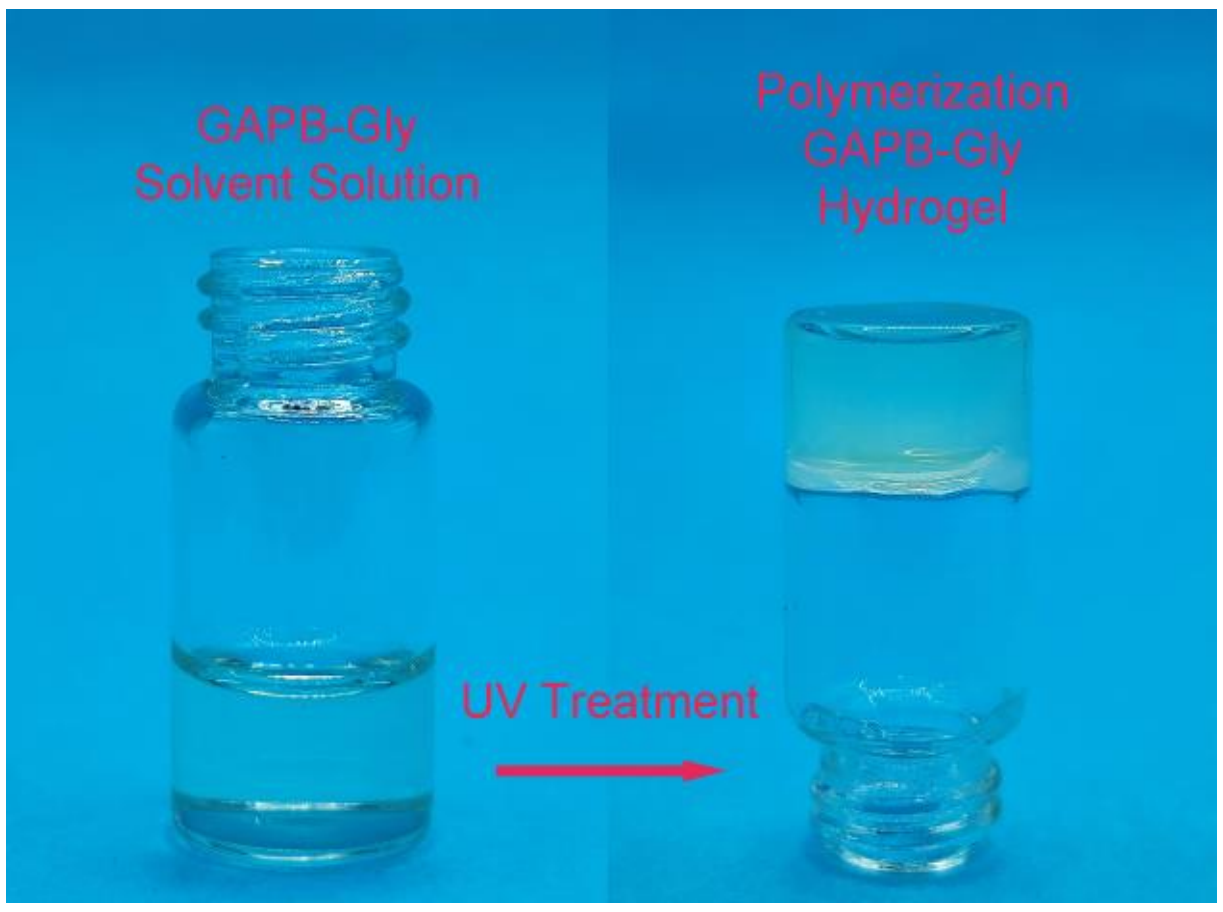

Figure S1. Optical photographs of GAPB-Gly hydrogel precursors (left) and crosslinked hydrogel (right) under visible light before and after UV-induced polymerization.

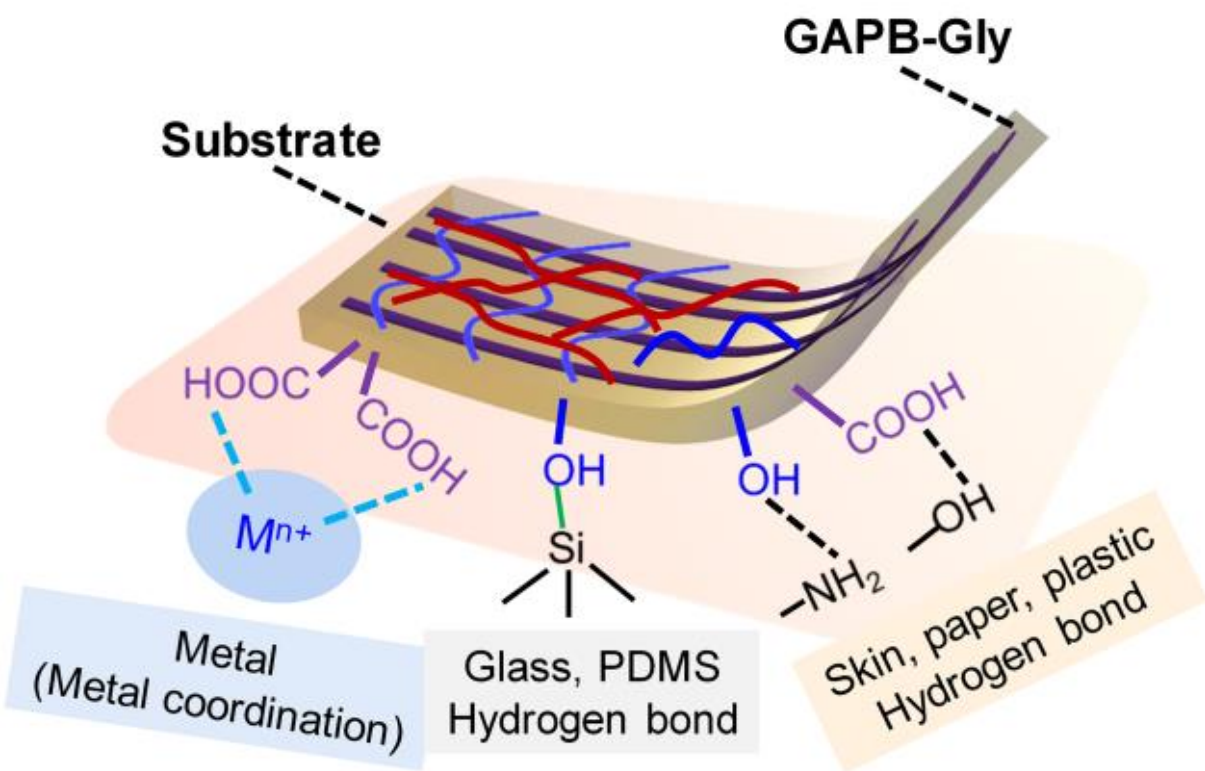

Figure S2. Schematic illustration of the self-adhesion mechanism of the GAPB-Gly hydrogel.

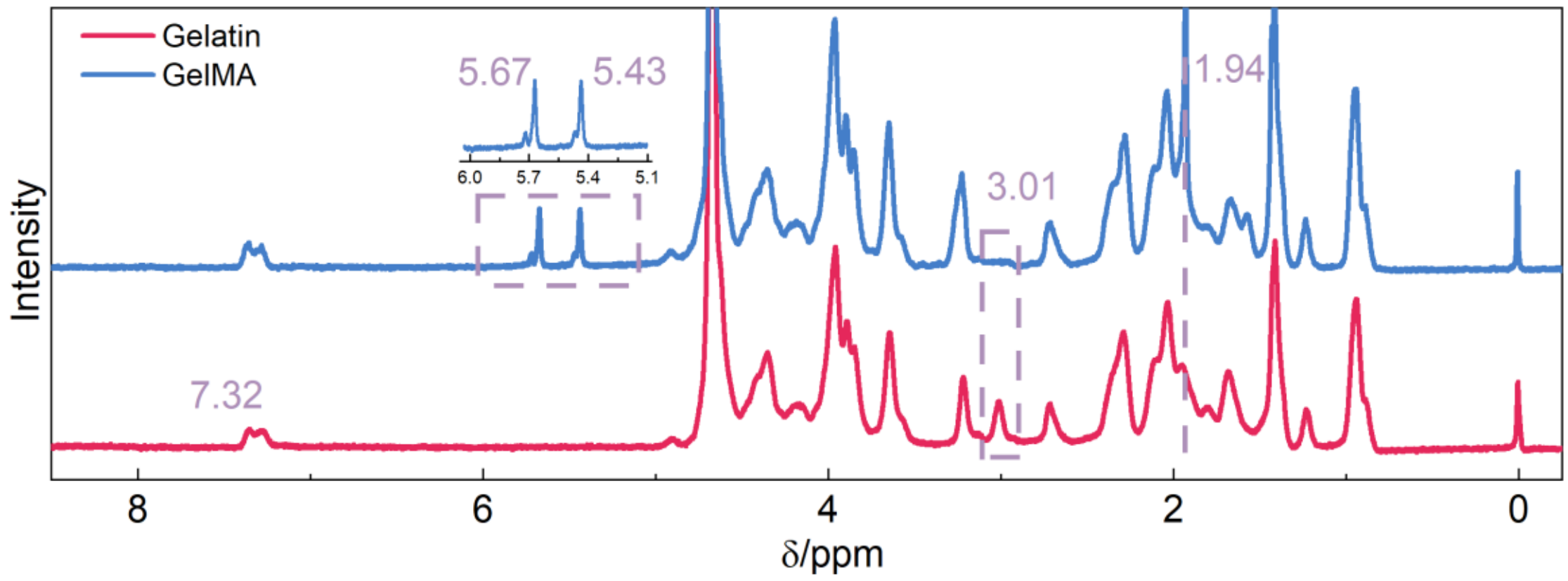

Figure S3. The ^1H NMR spectra of gelatin and GelMA. New peaks at 5.43 and 5.67 ppm corresponded to the vinyl protons of methacrylate groups introduced by methacrylic anhydride, while the decreased signal at 3.01 ppm was attributed to the methylene protons of lysine residues consumed during the methacrylation reaction.

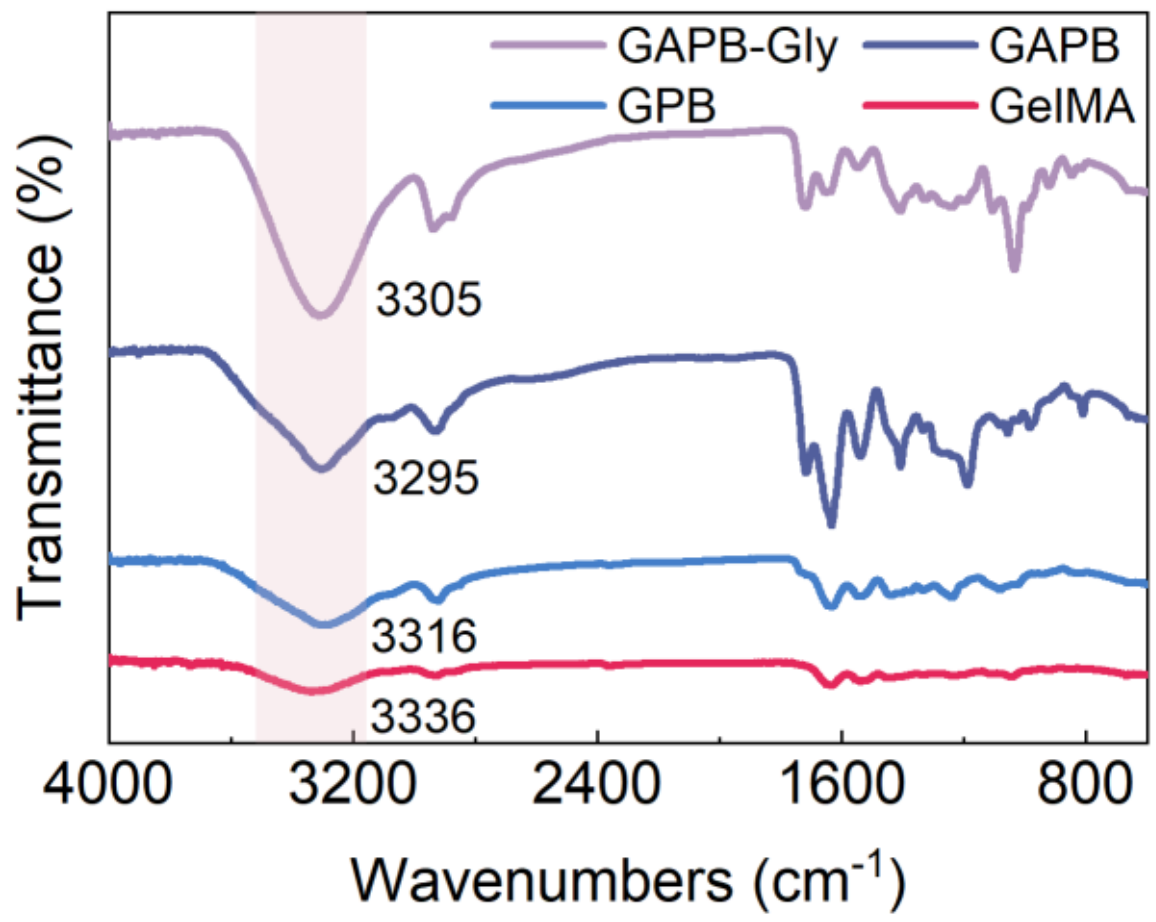

Figure S4. FTIR spectra of the GAPB-Gly hydrogel and its individual component samples. The peaks highlighted in the pink region represented hydrogen-bonding interactions among the different samples, which mainly originated from O–H and N–H stretching vibrations.

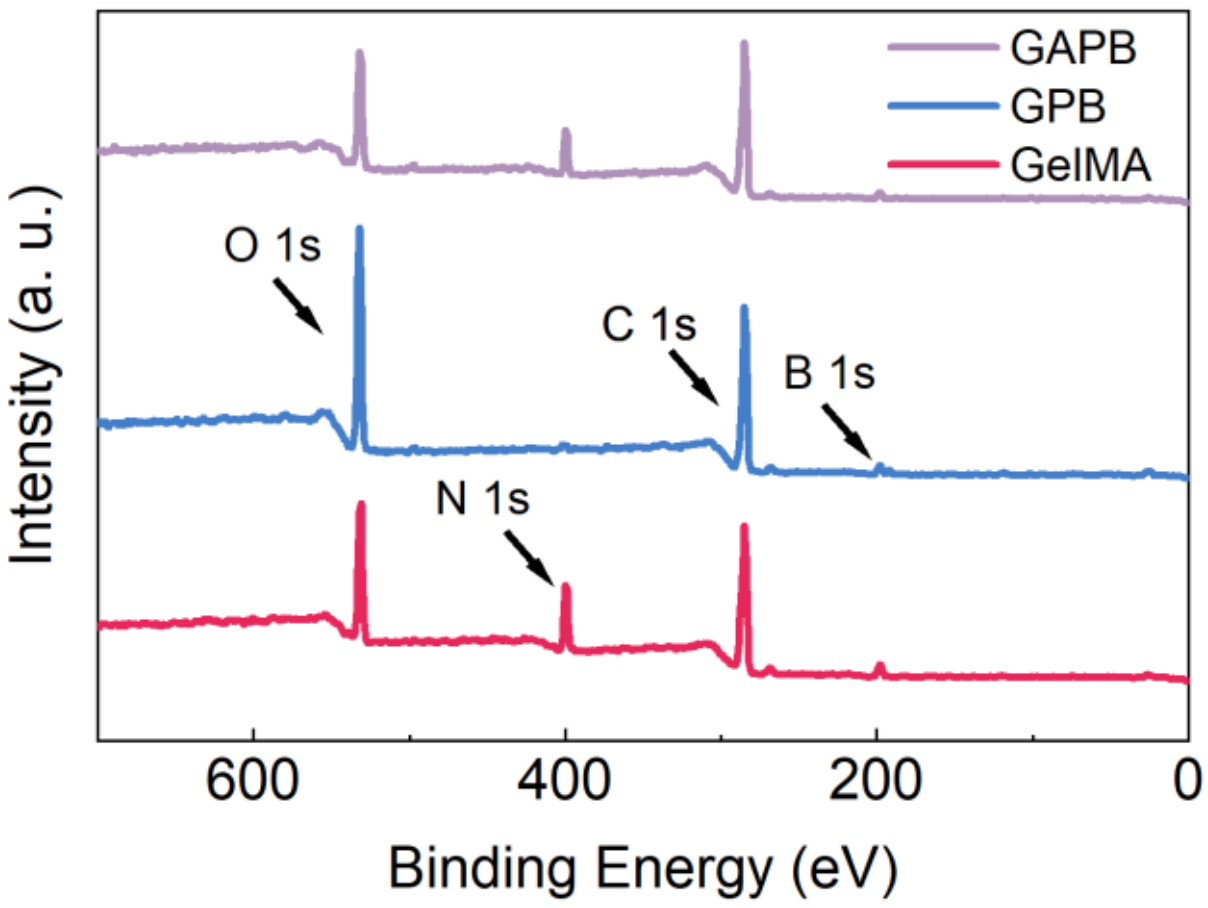

Figure S5. XPS spectra of GelMA, GPB hydrogel, and GAPB hydrogel. The spectra displayed the characteristic O 1s, N 1s, C 1s, and B 1s peaks of the samples, respectively.

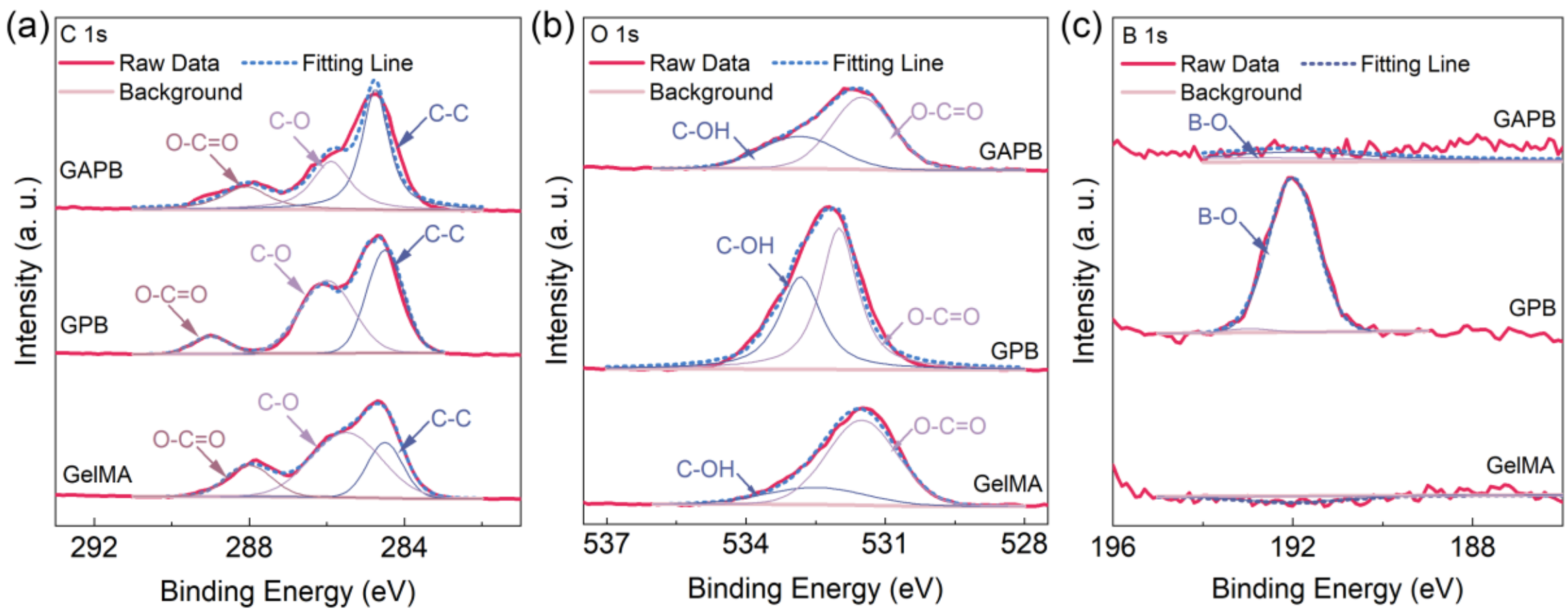

Figure S6. The high-resolution XPS of (a) C 1s, (b) O 1s, and (c) B 1s. The C 1s peak was deconvoluted into C–C, C–O, and O–C=O components; the O 1s peak was deconvoluted into C–OH and O–C=O components; and the B 1s peak was mainly attributed to B–O bonds.

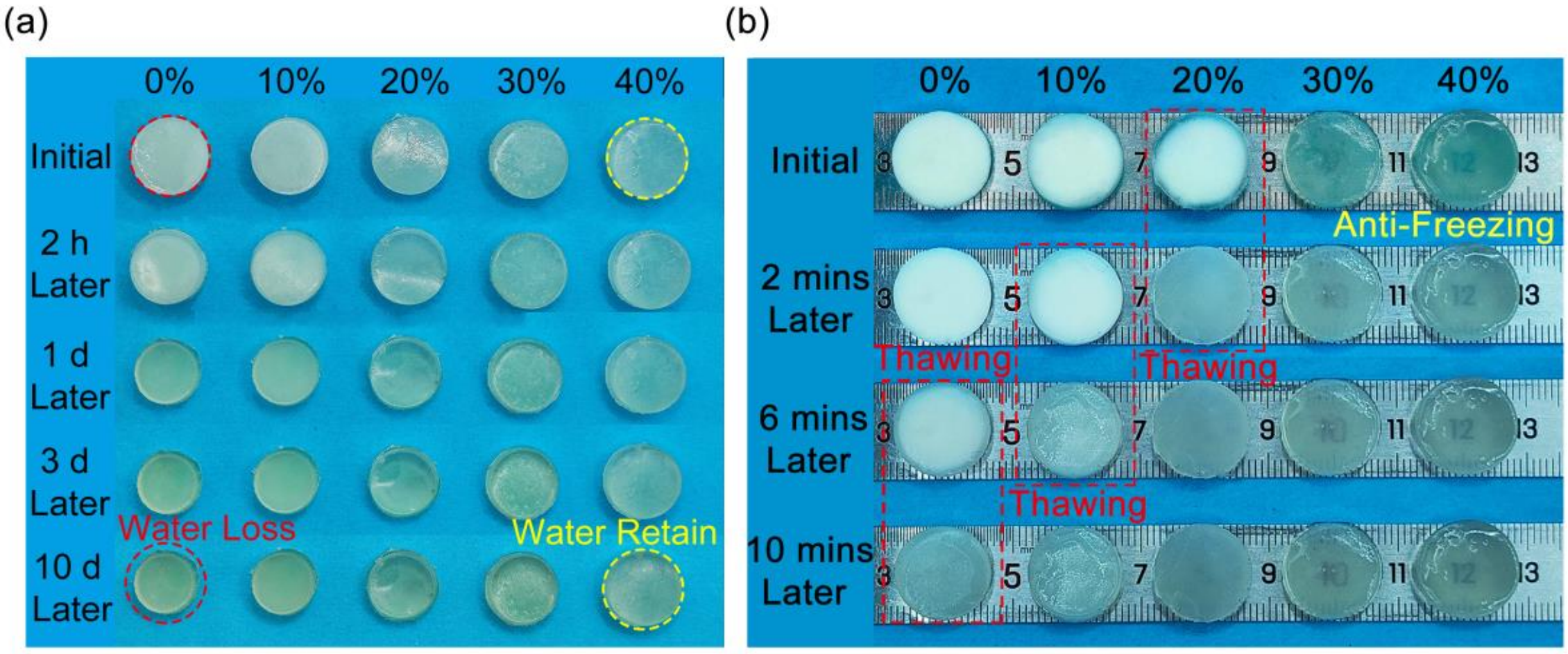

Figure S7. Moisturizing and anti-freezing performance of GAPB-Gly hydrogel with different glycerol concentrations. (a) Optical images of samples with glycerol concentrations of 0%, 10%, 20%, 30%, and 40% v/v stored at 25 ℃, 30% RH over 10 days. After 10 days, the sample with 0% v/v glycerol content exhibited significant volume reduction due to dehydration, while the sample with 40% v/v glycerol content maintained its original volume. (b) Optical images of hydrogels with glycerol concentrations of 0%, 10%, 20%, 30%, and 40% v/v after being frozen at -40 ℃ for 1 day and subsequently thawed at 25 ℃ for 10 min. Samples with glycerol concentrations below 20% v/v showed noticeable freezing and gradual thawing within 10 min, whereas the sample with 40% v/v glycerol content exhibited no significant morphological changes. All cylindrical specimens employed in this study were fabricated with standardized dimensions of 15 mm in diameter and 2 mm in height.

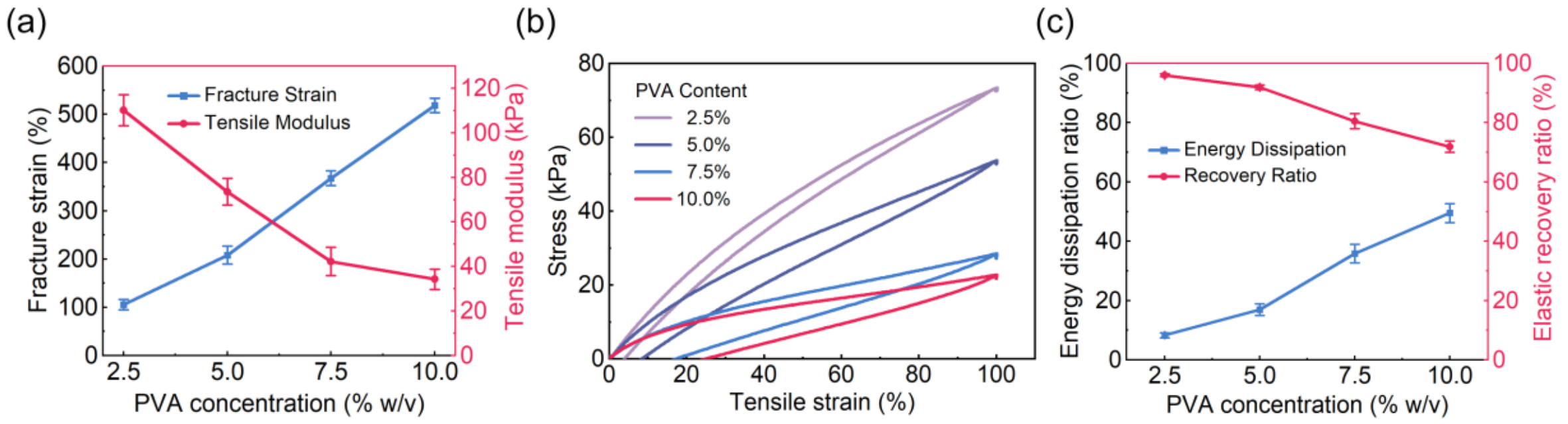

Figure S8. Influence of PVA concentration on the tensile properties of GAPB-Gly hydrogel. (a) Fracture strain and tensile modulus of hydrogels with varying PVA concentrations. (b) Loading-unloading curves for hydrogels with different PVA concentrations. (c) Energy dissipation ratio and elastic recovery ratio of hydrogels with varying PVA concentrations. Throughout the test, the AA concentration was maintained at 30%v/v.

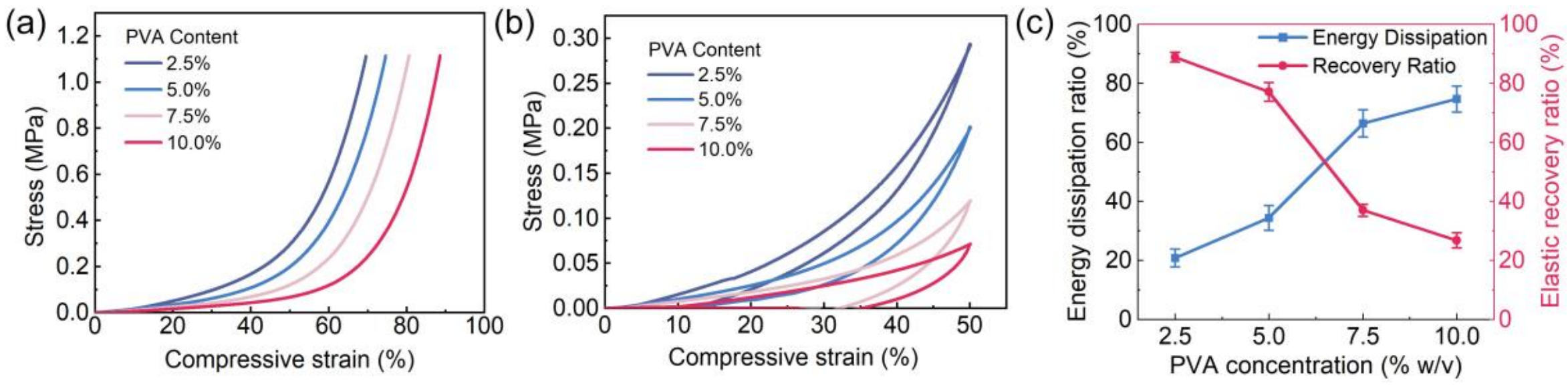

Figure S9. Influence of PVA concentration on the compressive properties of GAPB-Gly hydrogel. (a) Compressive strain and stress of hydrogels with varying PVA concentrations. (b) Loading-unloading curves for hydrogels with different PVA concentrations. (c) Energy dissipation ratio and elastic recovery ratio of hydrogels with varying PVA concentrations. Throughout the test, the AA concentration was maintained at 30% v/v.

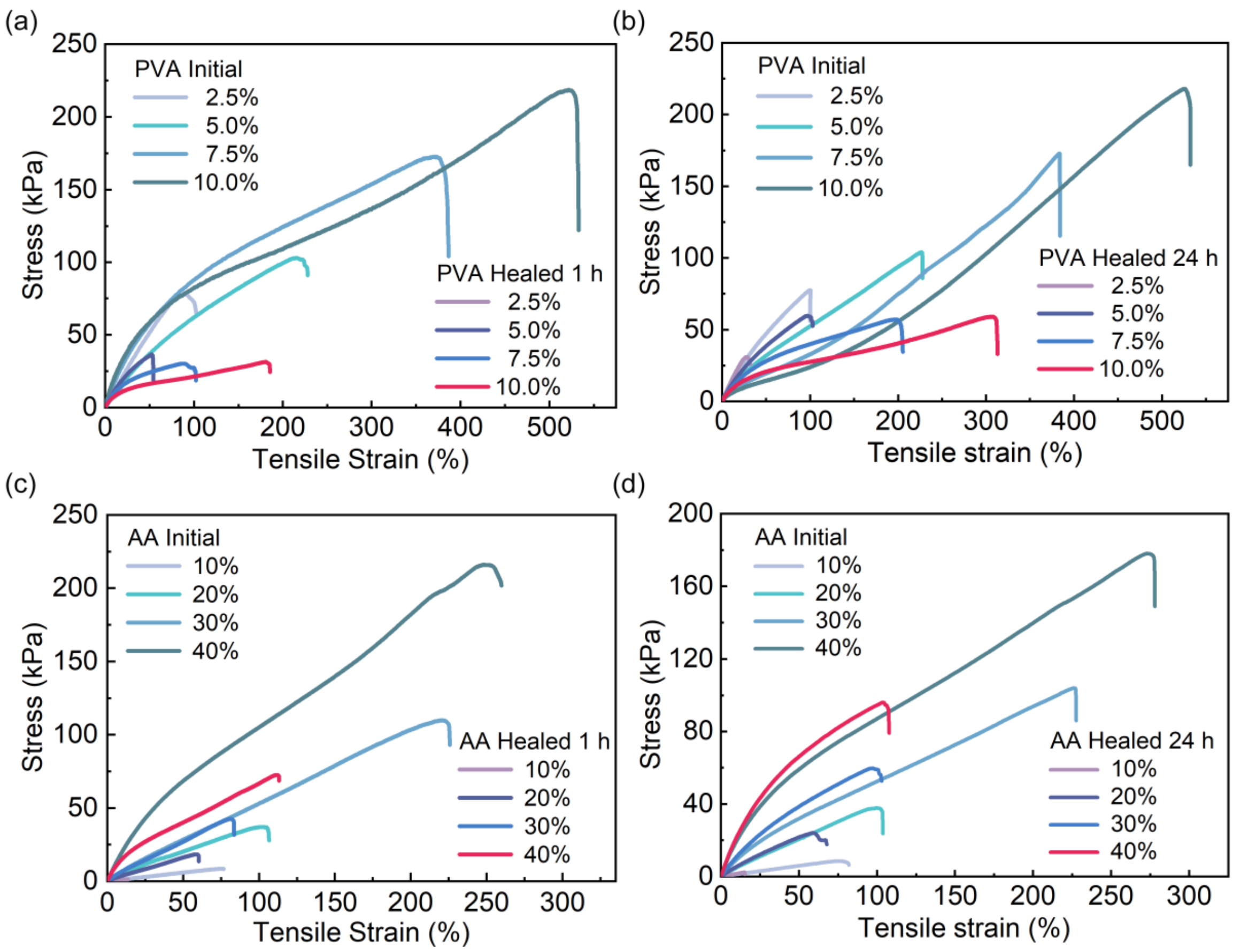

Figure S10. Effects of component concentration on the self-healing properties of GAPB-Gly hydrogel. Tensile curves of GAPB-Gly hydrogel with varying PVA concentrations of 2.5%, 5.0%, 7.5%, and 10.0% w/v before and after (a) 1 h and (b) 24 h of healing. Tensile curves of GAPB-Gly hydrogel with varying AA concentrations of 10%, 20%, 30%, and 40% v/v before and after (c) 1 h and (d) 24 h of healing. After 1 h healing, the hydrogels with 2.5%, 5.0%, 7.5%, and 10.0% w/v PVA achieved SHE of 19.6%, 35.3%, 35.8%, and 39.1%, respectively. Meanwhile, for the hydrogels with 10%, 20%, 30%, and 40% v/v AA, the SHE were 10.8%, 46.4%, 35.8%, and 33.8%, respectively.

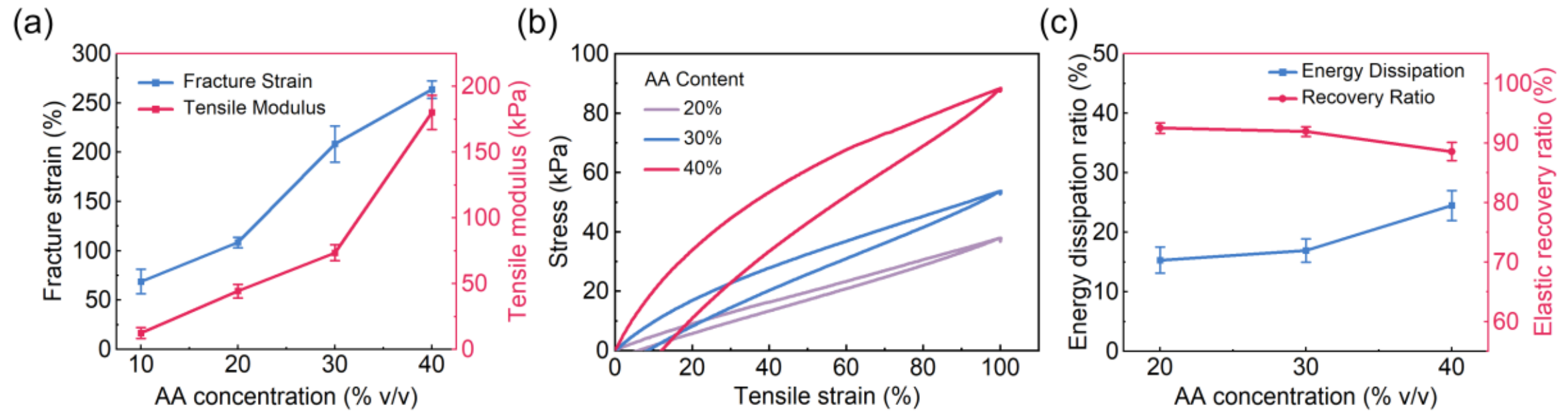

Figure S11. Influence of AA concentration on the mechanical properties of GAPB-Gly hydrogel. (a) Fracture strain and tensile modulus of hydrogels with varying AA concentrations. (b) Loading-unloading curves for hydrogels with different AA concentrations. (c) Energy dissipation ratio and elastic recovery ratio of hydrogels with varying AA concentrations. Throughout the test, the PVA concentration was maintained at 5% w/v.

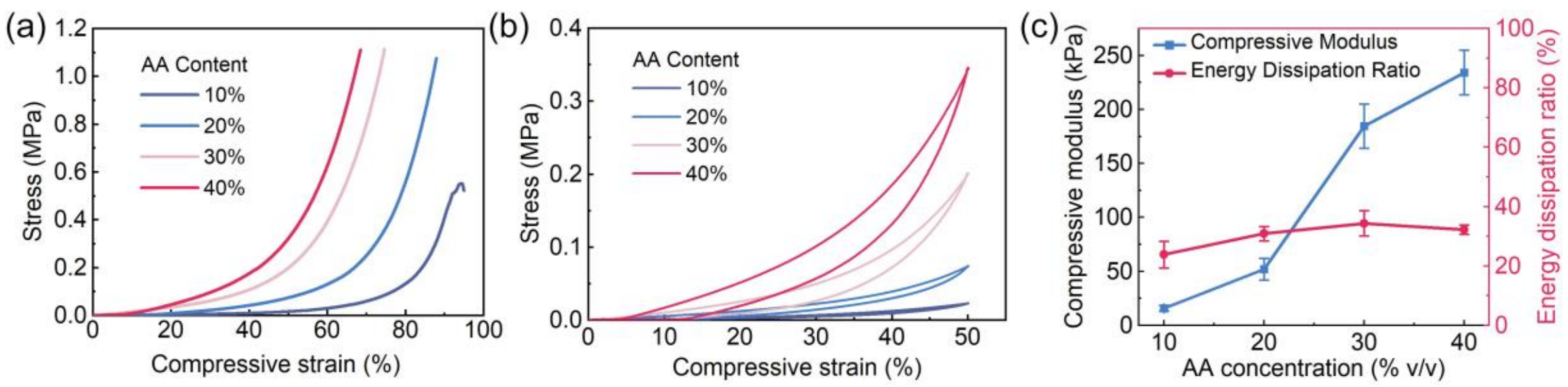

Figure S12. Influence of AA concentration on the compressive properties of GAPB-Gly hydrogel. (a) Compressive strain and modulus of hydrogels with varying AA concentrations. (b) Loading-unloading curves for hydrogels with different AA concentrations. (c) Energy dissipation ratio and compressive modulus of hydrogels with varying AA concentrations. Throughout the test, the PVA concentration was maintained at 5% w/v.

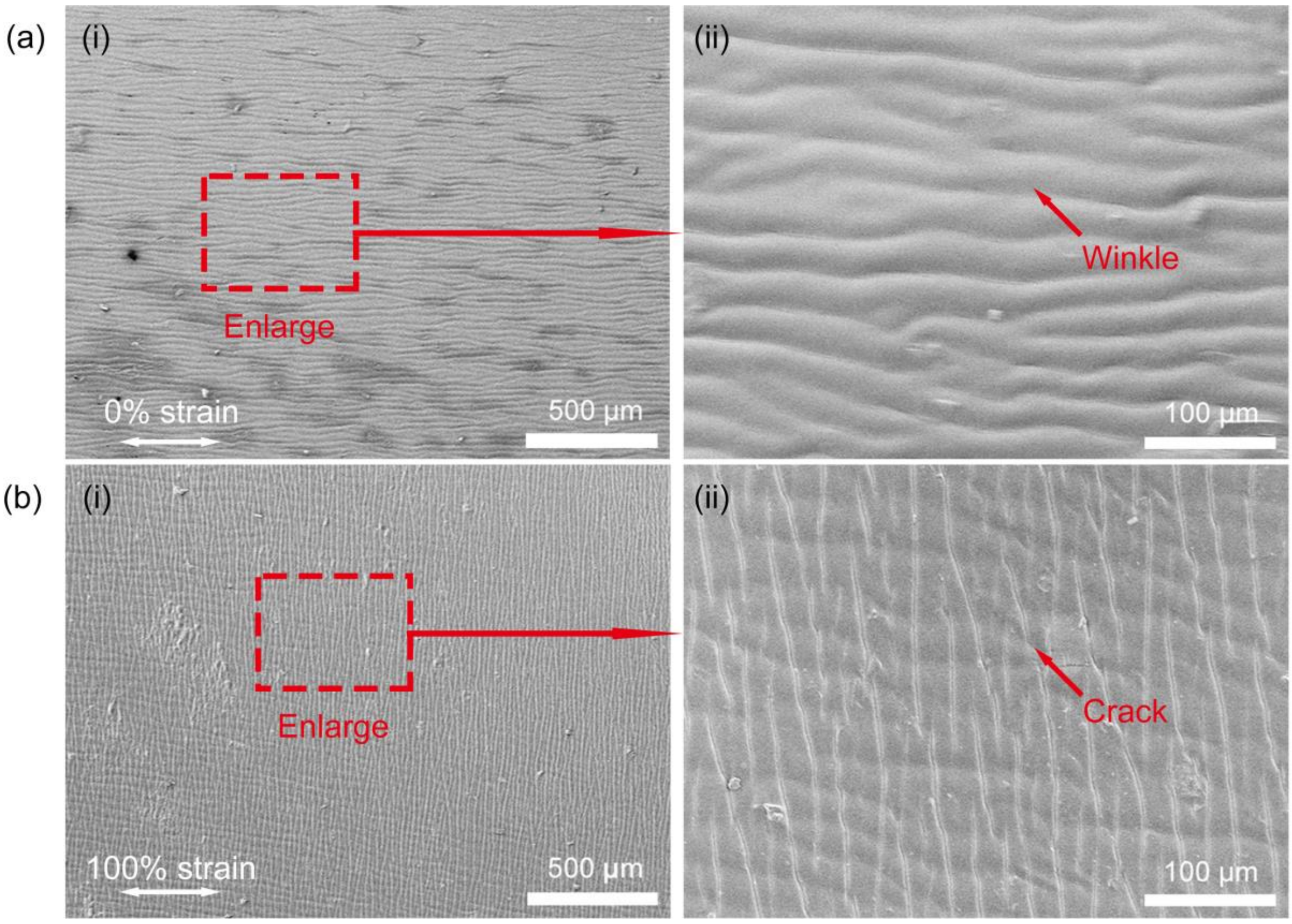

Figure S13. SEM Images of surface morphology of optimized GAPB-Gly hydrogel in the initial state and under 100% strain. (a) Surface morphology image and magnified view at 0% strain, revealing aligned fibrous structures. (b) Surface morphology image and magnified view at 100% strain, depicting a mesh-like network of intersecting fibers.

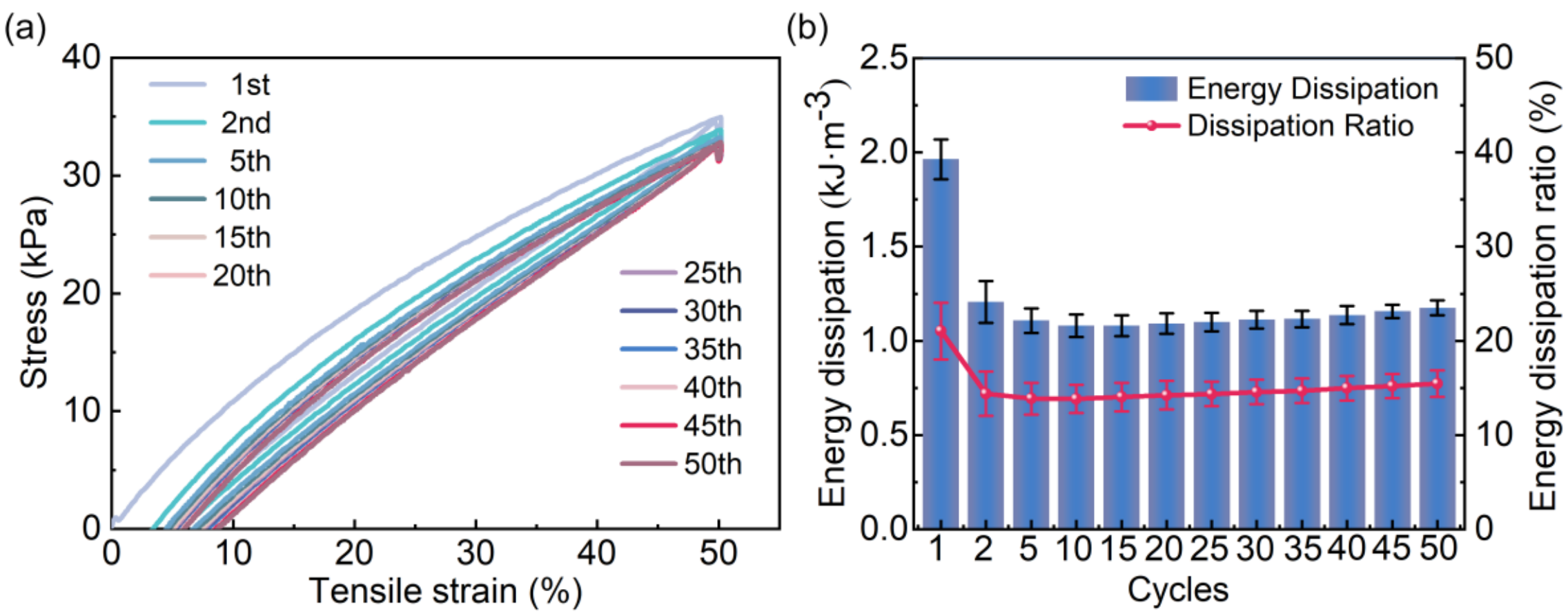

Figure S14. Mechanical performance of optimized GAPB-Gly hydrogel under 50 cycles

of 50% strain. (a) Stress-strain curves. (b) Energy dissipation and dissipation ratio curves.

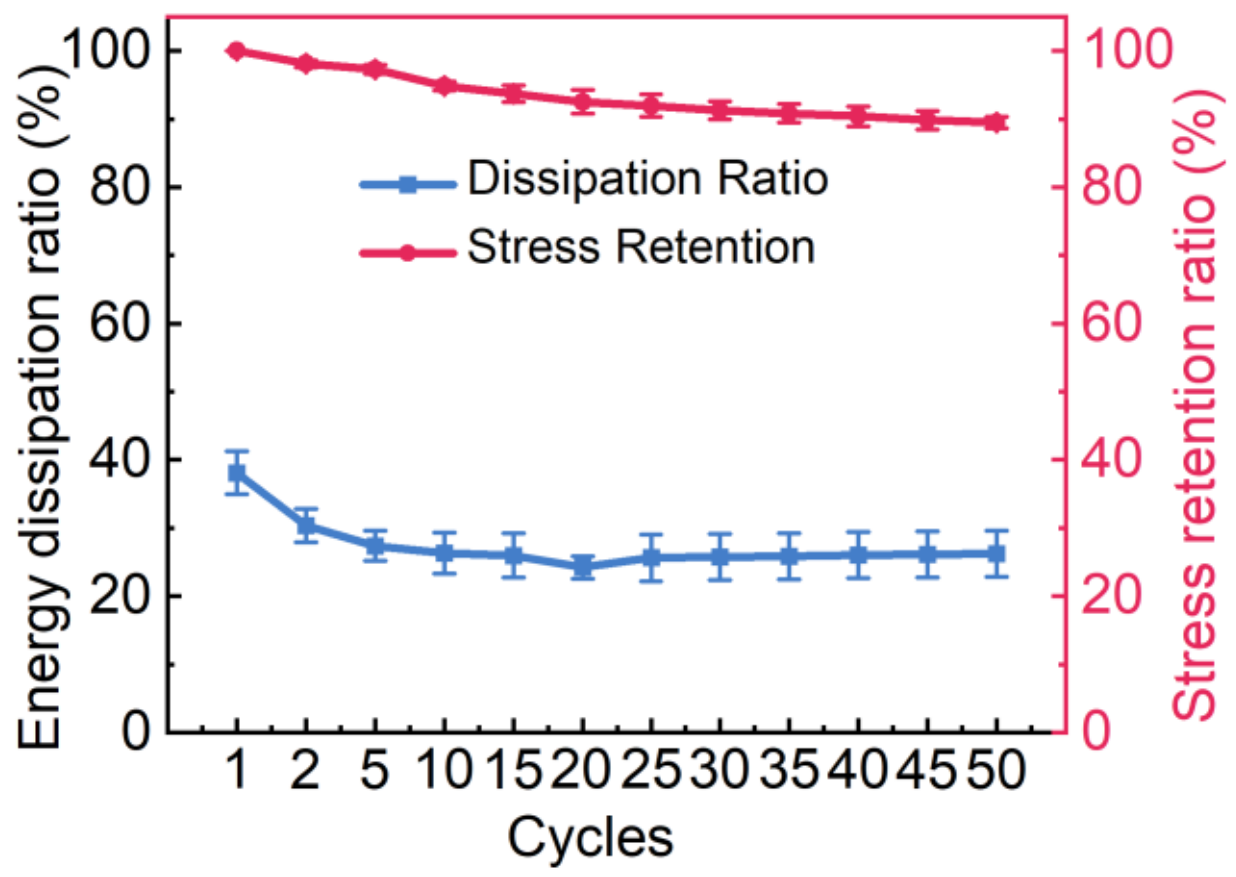

Figure S15. Energy dissipation ratio and stress retention ratio of optimized GAPB-Gly hydrogel under 50 cycles of 50% compressive strain.

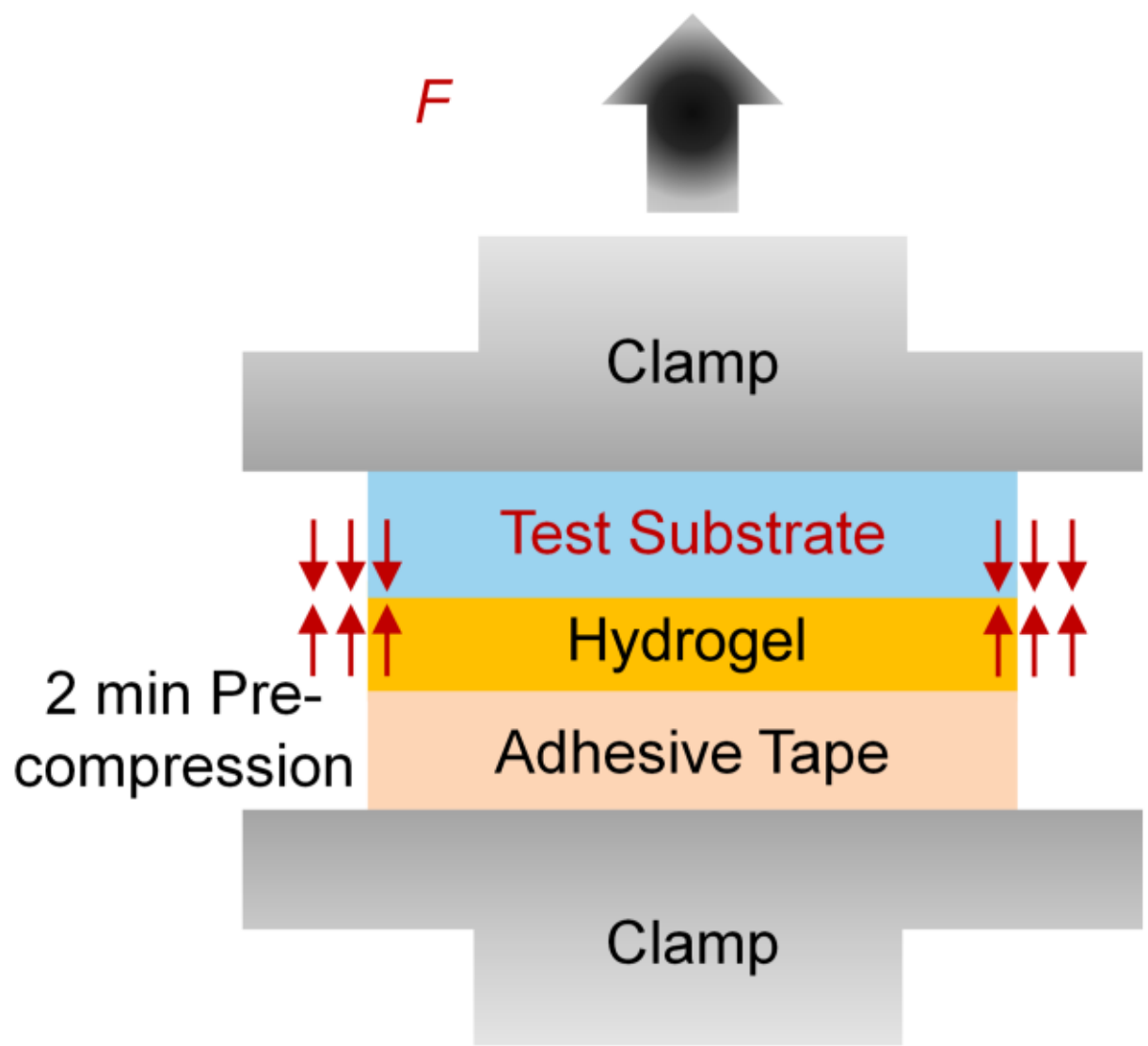

Figure S16. Schematic illustration of the axial tensile adhesion test. Following the established standard, we selected cylindrical hydrogel samples with a diameter of 15 mm and a height of 2 mm [S1]. The samples are fixed using tape, and after 2 minutes of pre-

compression, the tensile tester is set to a stretching speed of 50 mm/min for adhesion strength testing.

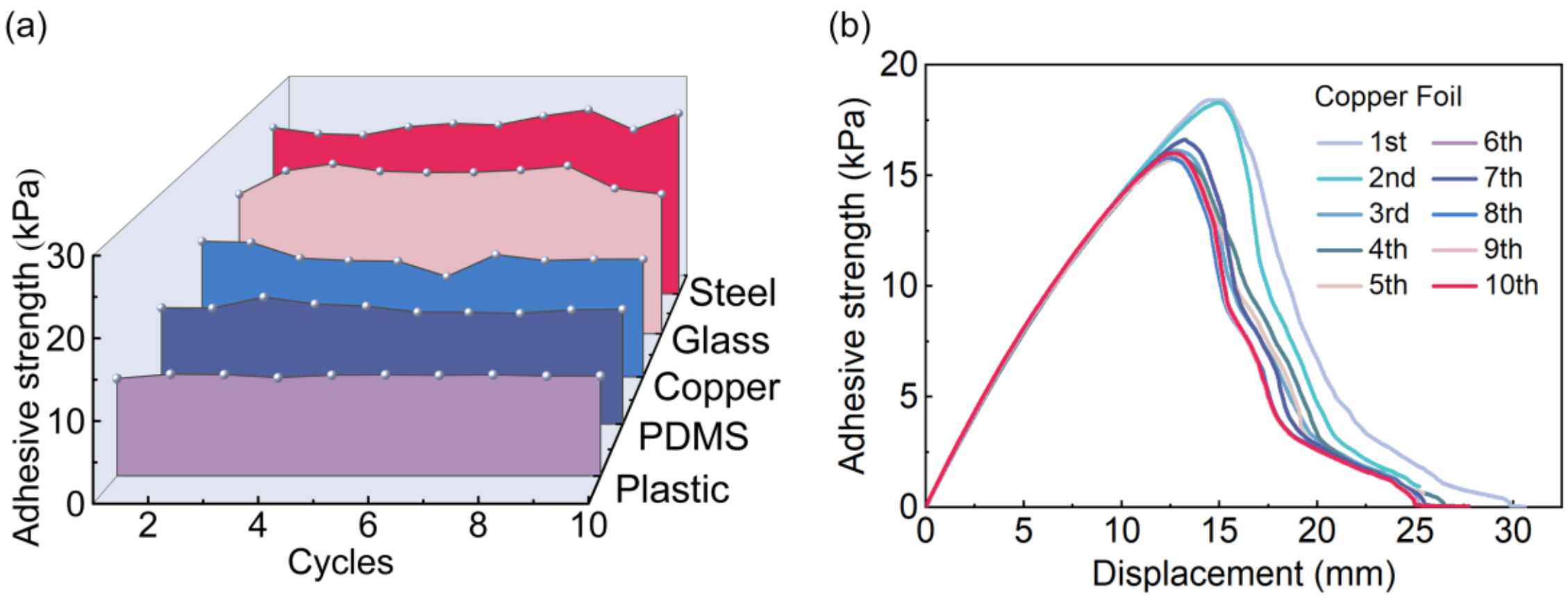

Figure S17. Multi-interface repeated adhesion tests. (a) Adhesion strength of GAPB-Gly hydrogel on various surfaces (plastic, PDMS, copper, glass, steel) after repeated adhesion tests. The same cylindrical samples were used for the measurements. (b) Adhesion strength curves of GAPB-Gly hydrogel on copper foil were measured over 10 repeated cycles. The tests were conducted under controlled conditions at 25 ℃ and 30% RH.

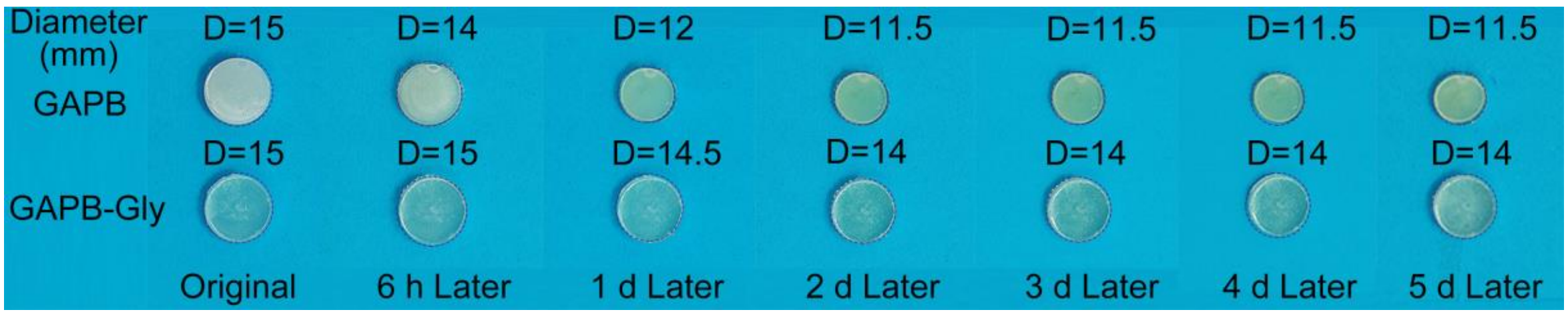

Figure S18. Optical images showcasing the moisturizing properties of the GAPB-Gly hydrogel. Under storage conditions of 25 ℃, 30% RH, the GAPB hydrogel exhibited significant dehydration after 5 days, with its diameter decreasing from 15 mm to 11.5 mm after 5 days. In contrast, the GAPB-Gly hydrogel maintained morphological stability throughout the same period.

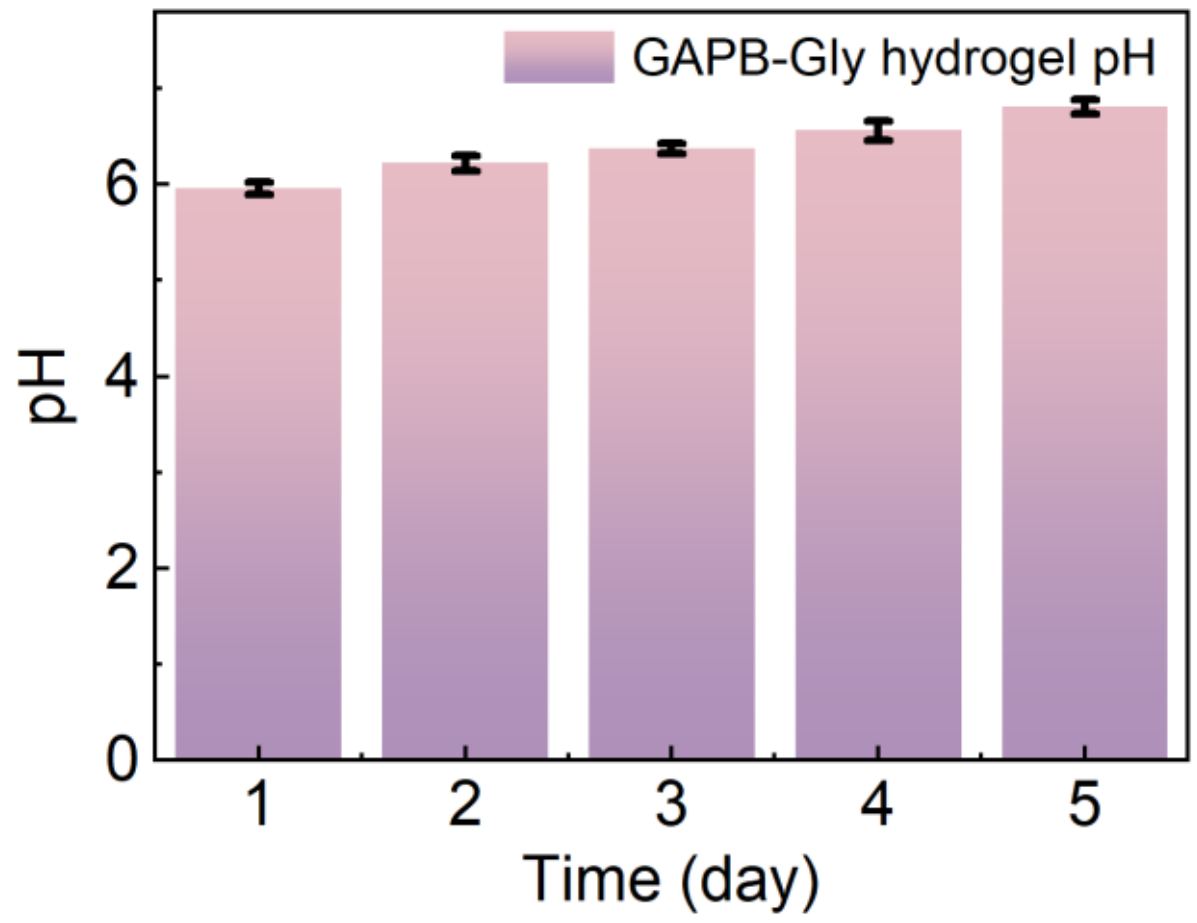

Figure S19. pH variations in the supernatant of GAPB-Gly hydrogel immersion solution. The pH of the supernatant was periodically monitored over a 5-day incubation in DI water at 25 ℃, with the soaking liquid entirely refreshed after each measurement to drive continuous leaching. n=5.

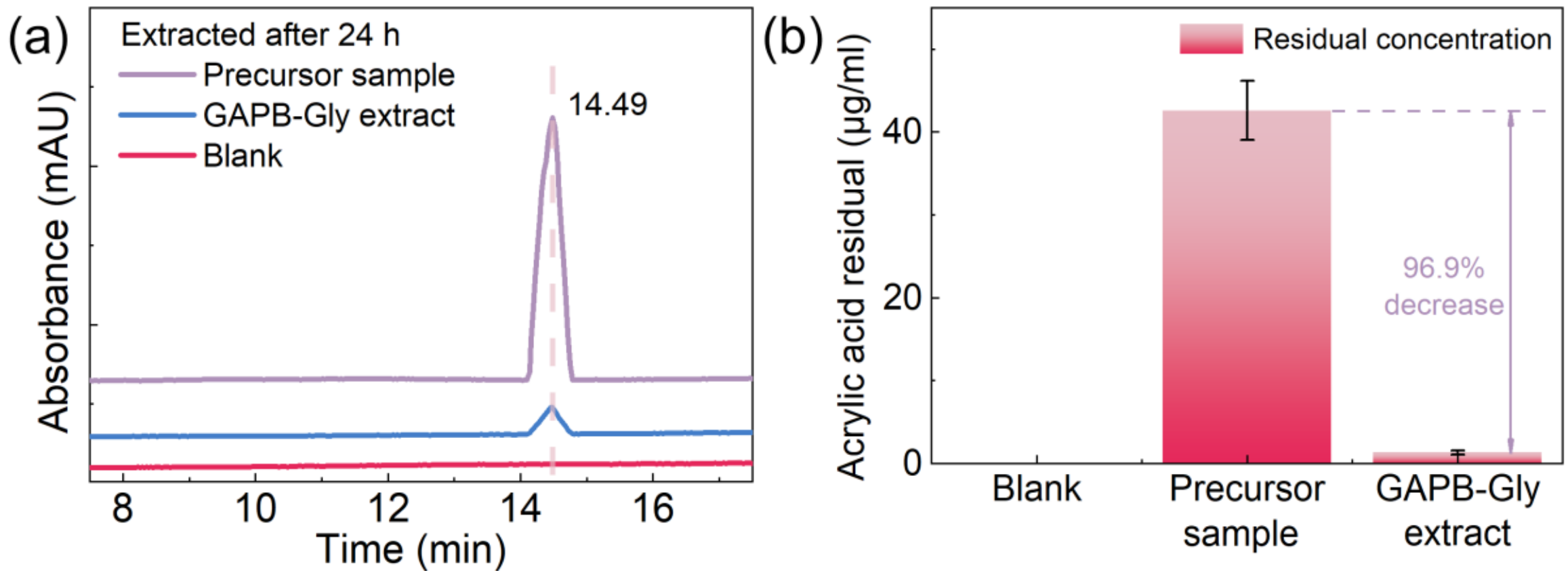

Figure S20. Quantitative analysis of residual AA monomer in the GAPB-Gly hydrogel. (a) HPLC chromatograms of the supernatant. (b) Concentration of the leached AA monomer. n=5.

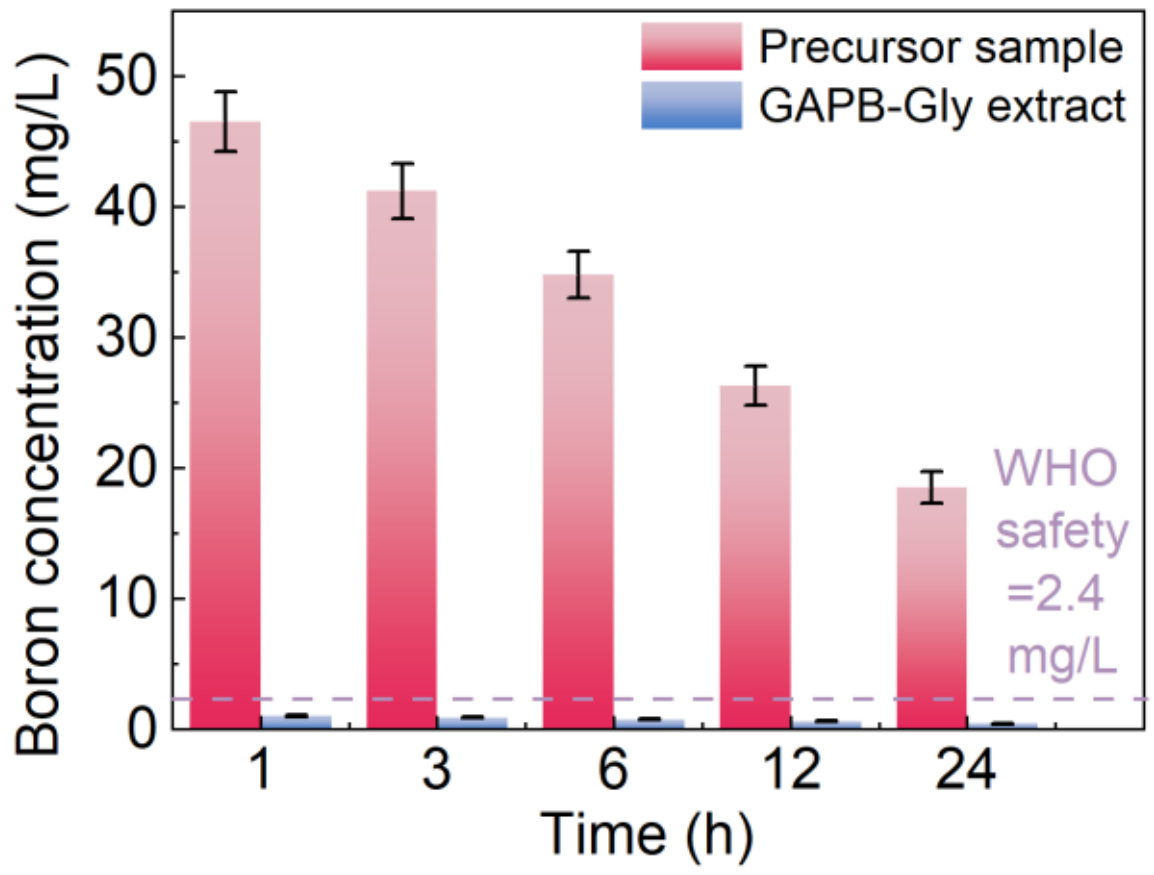

Figure S21. Time-dependent boron release from the GAPB-Gly hydrogel. n=5. The boron concentration for the Blank group remained consistently non-detectable (N.D., below the instrument detection limit) across all time points.

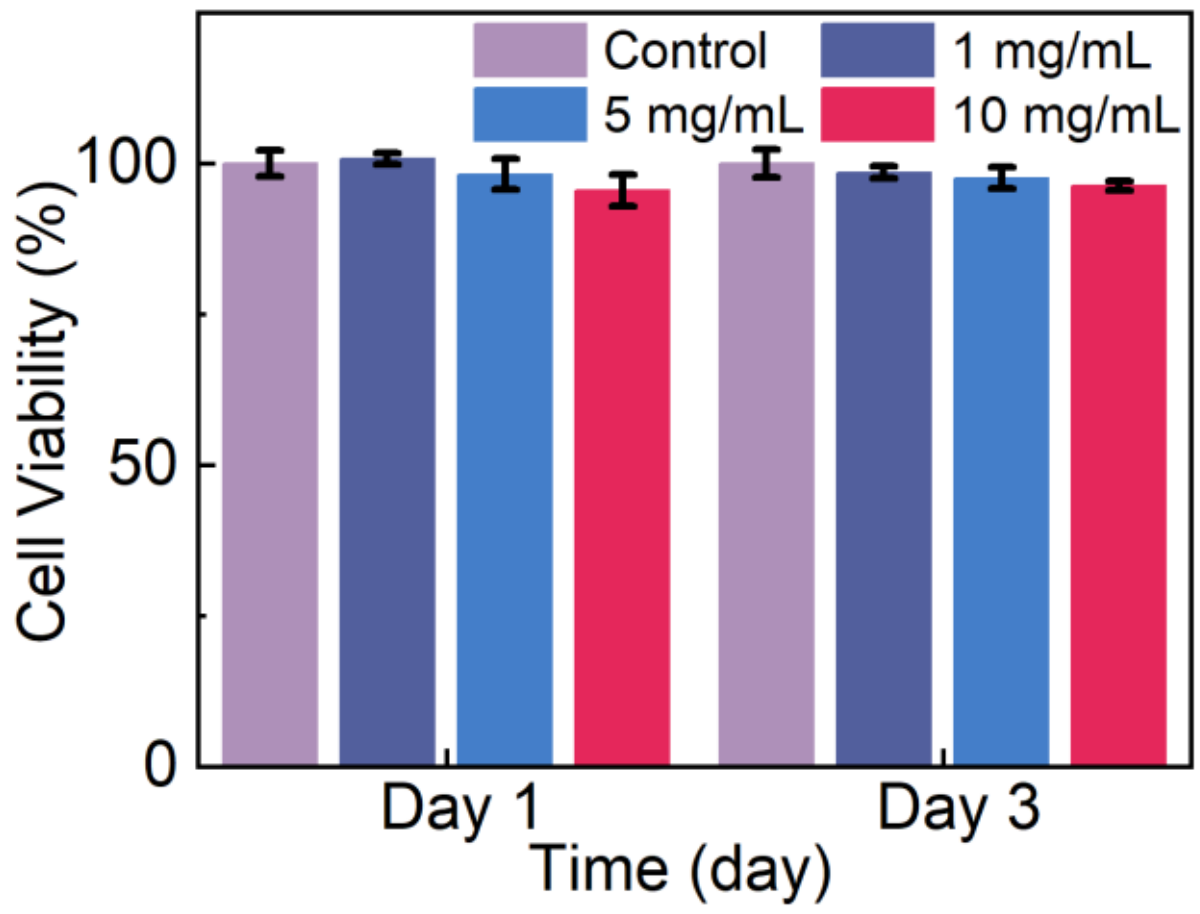

Figure S22. Cell viability of L929 cells after co-incubating with different concentrations (1, 5 and 10 mg/mL) of hydrogel extracts for 1 and 3 days. n=5.

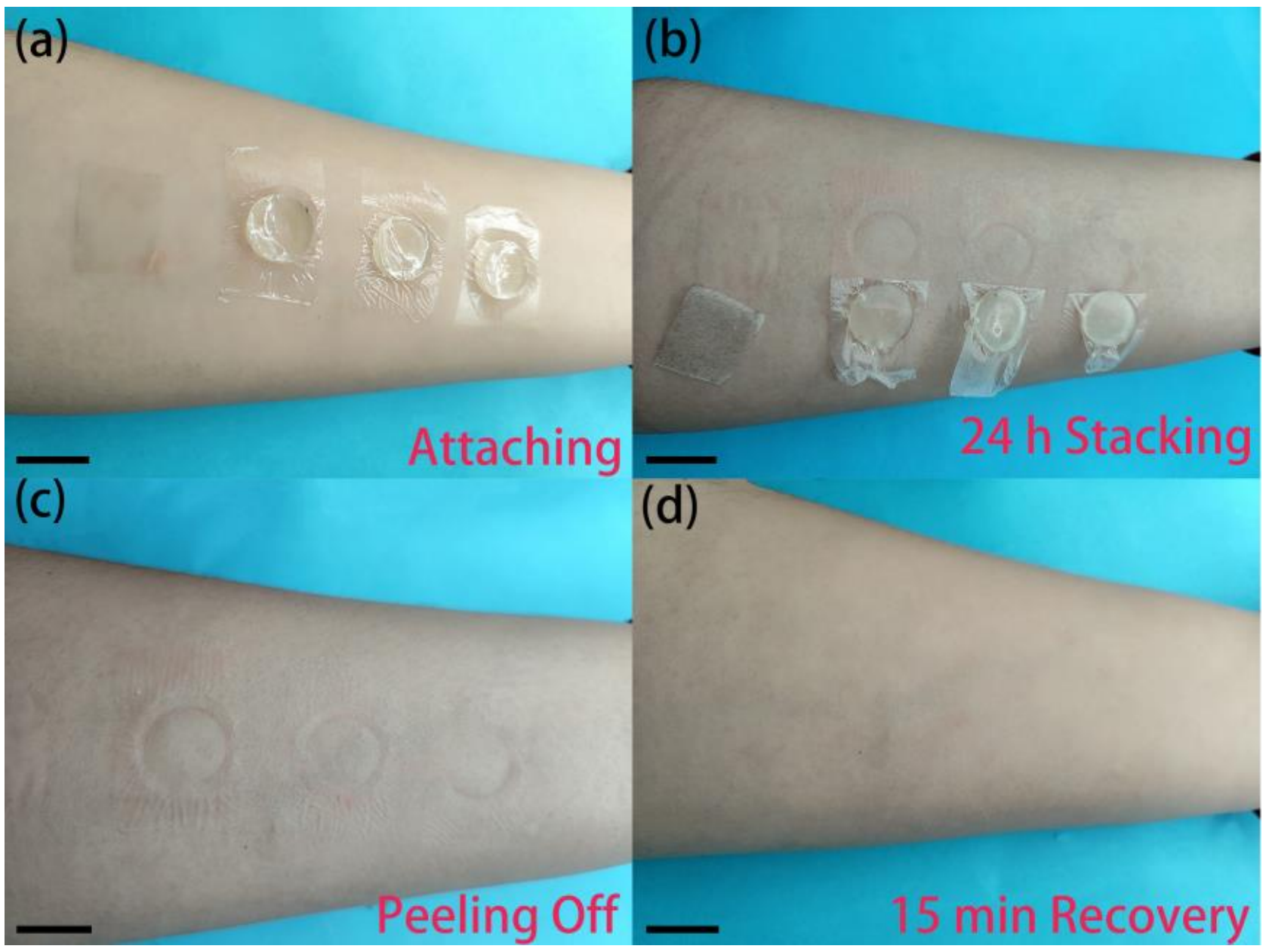

Figure S23. Skin irritation test of the GAPB-Gly hydrogel. (a) Optical photograph of the hydrogel fixed on the forearm of a volunteer using medical PU tape; (b) optical photograph after peeling off the tape and hydrogel following 24 h of attachment; (c) skin surface indentation after removal; (d) photograph after 15 min of recovery, showing no obvious erythema or allergic reaction. The scale bar in the figure represents 15 mm.

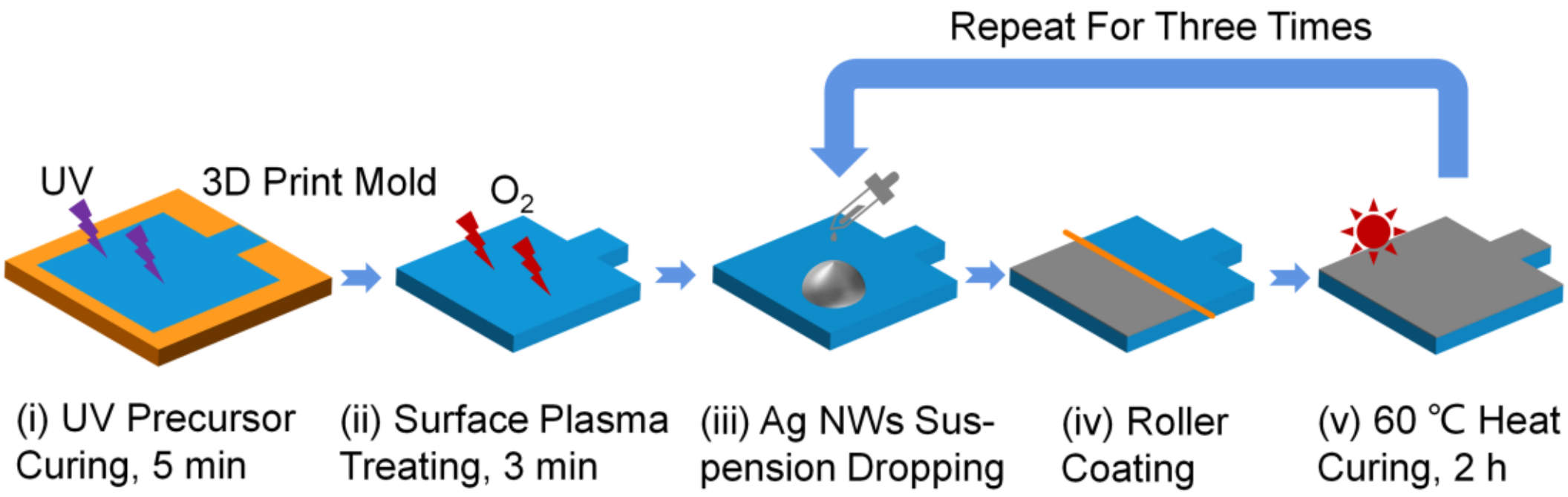

Figure S24. The fabrication process of the Ag NWs/GAPB-Gly composite electrode. Through a 3D-printed mold, the 2×2 cm^2 $GA_{30}P_5B\text{-}Gly_{40}$ substrate was obtained with 5 min of UV cross-linking (Figure i). Subsequently, the surface of the substrate undergoes 3

min of oxygen plasma treatment (Figure ii) to achieve a hydrophilic surface. Following this, 10 mg/mL silver nanowire suspension was dropped onto the surface (Figure iii) and rolled with a glass plate (Figure iv) to form a uniform conductive layer. Finally, the substrate was placed on a 60 ℃ hot plate for 2 h to cure. To achieve a well-conductive surface, the drop-coating and heating steps were repeated three times, resulting in a self-healable of Ag NWs/GAPB-Gly composite electrode.

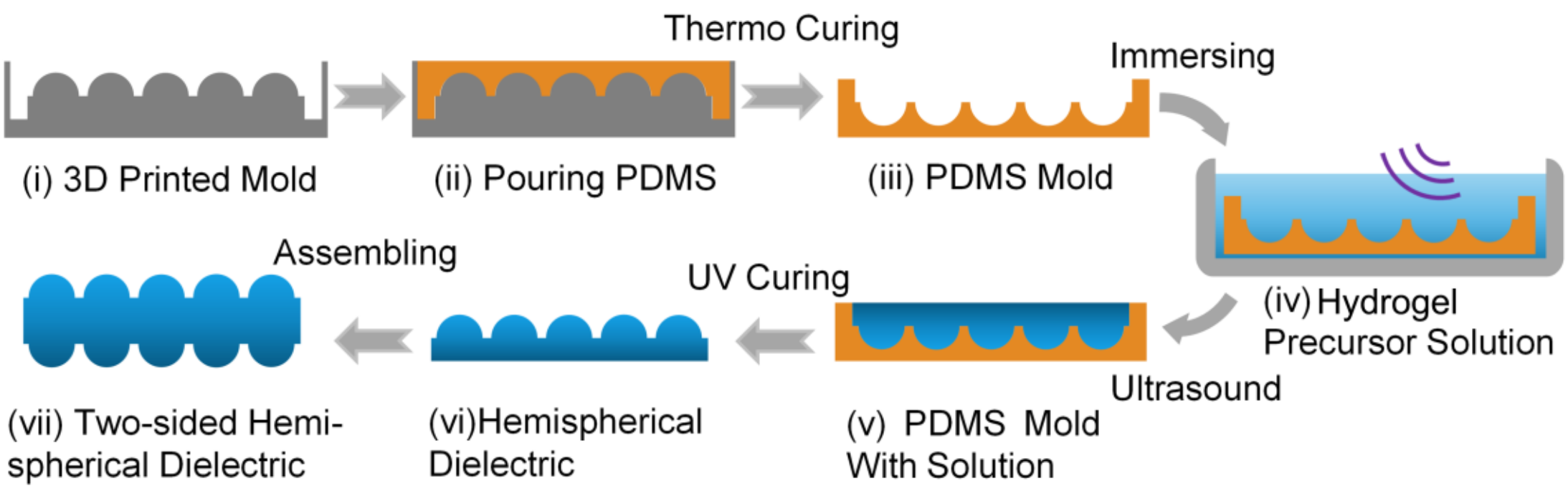

Figure S25. Fabrication process of the two-sided hemispherical dielectric layer. The whole process was prepared using a secondary molding method [S2]. First, a mold with a hemisphere diameter of 600 μm and an inter-hemisphere spacing of 800 μm was created using 3D printing (Figure i). The mold was subsequently treated with a cationic surfactant (hexadecyltrimethylammonium bromide, shanghai macklin biochemical Co., Ltd.) to achieve a self-assembled surface. PDMS was then cast into the mold and cured at 80 ℃ for 1 hour in a vacuum drying oven (DZH-6050, shanghai yiheng scientific instrument Co., Ltd.) (Figure ii), resulting in a PDMS mold with a uniformly distributed hemispherical structure (Figure iii). The mold was subsequently immersed in the GAPB-Gly precursor solution containing 2.5% w/v NaCl and treated with vacuum ultrasonication (Figure iv) to

obtain a complete hemispherical structure (Figure v). The precursor was then cross-linked and molded by UV irradiation at an intensity of 50 mW/cm^2 for 5 minutes, forming a 2×2 cm^2 dielectric layer (Figure vi). Finally, the two dielectric layers were assembled to obtain a two-sided hemispherical dielectric layer due to the material's adhesive properties.

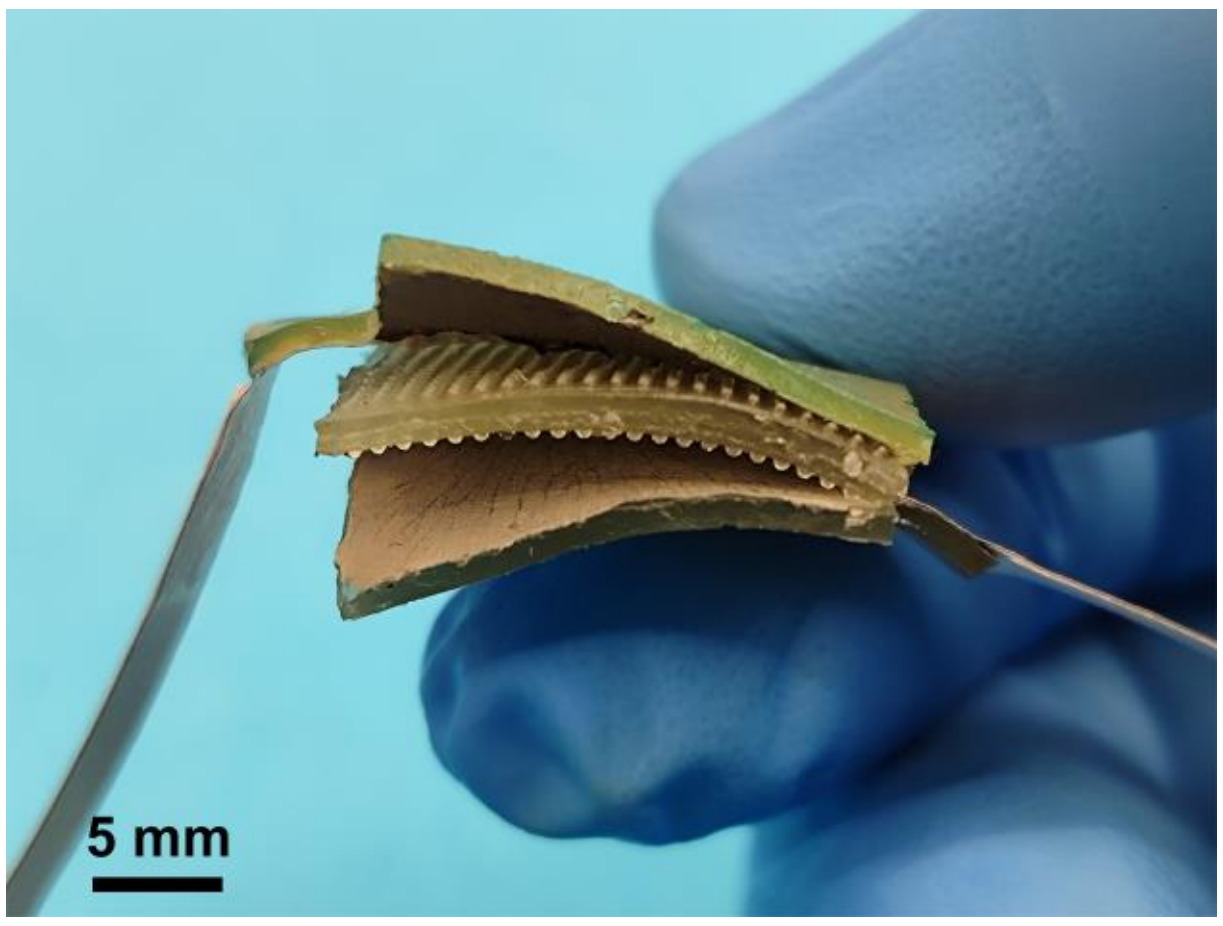

Figure S26. The physical assembly diagram of the OHI pressure sensor.

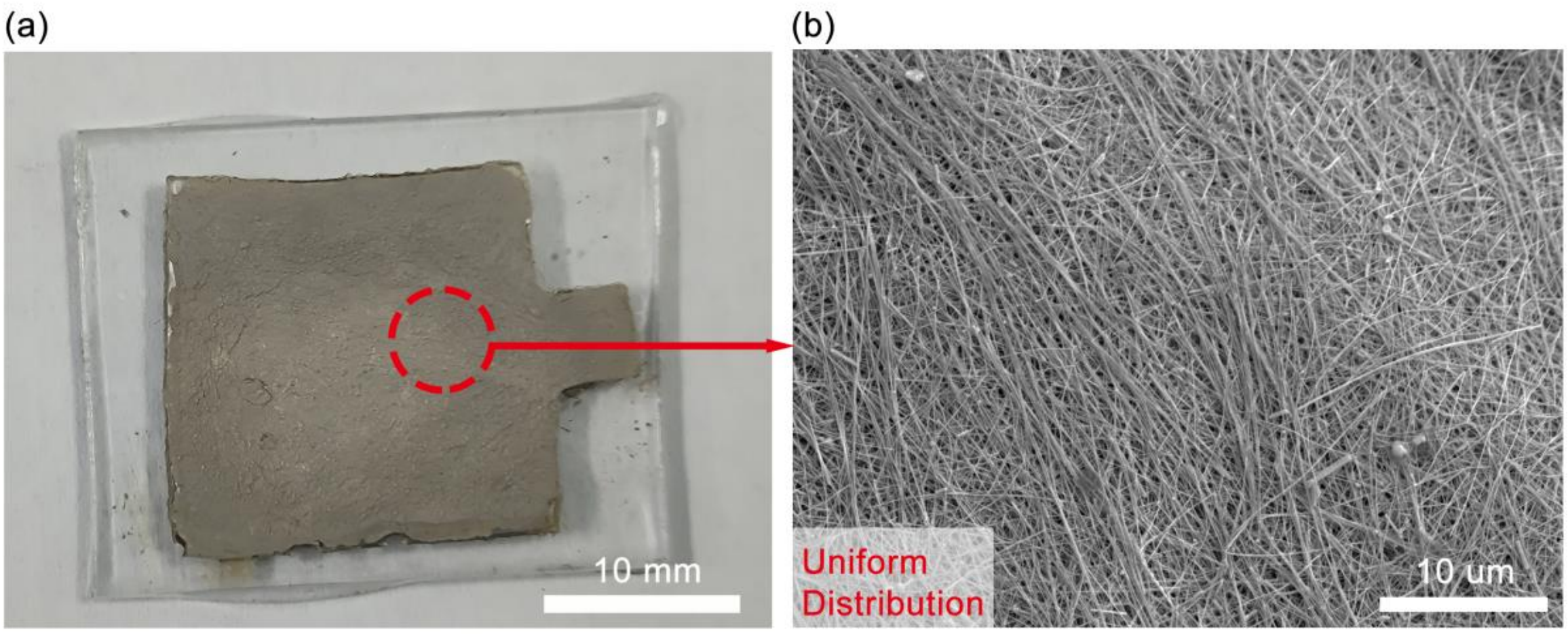

Figure S27. (a) Optical photograph and (b) SEM micrograph of the omni-healable GAPB-Gly hydrogel electrode surface coated with Ag-NWs.

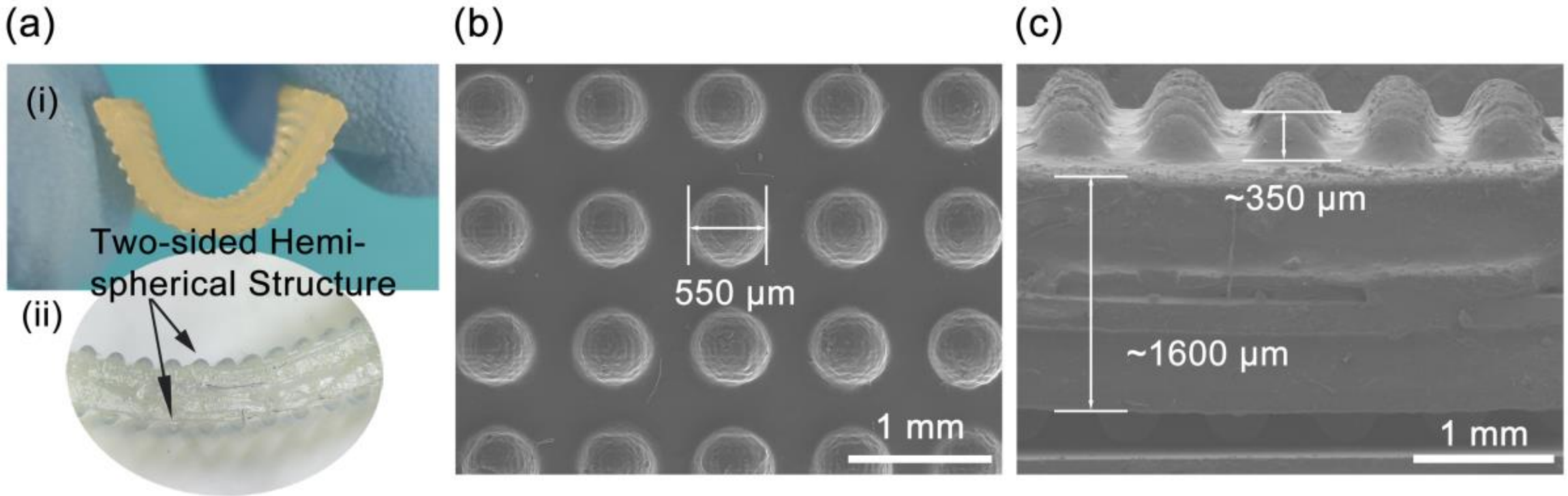

Figure S28. Characterization of the two-sided hemispherical dielectric layer. (**a**) Optical photograph shows the two-sided hemispherical structure of the dielectric layer. (**b**) SEM top-view image of the dielectric layer surface, revealing uniform and complete hemispherical structures with a diameter of 550 μm. (**c**) SEM cross-sectional image of the dielectric layer, demonstrating a total thickness of approximately 2300 μm, with hemispherical structures reaching a height of about 350 μm. The uniformly distributed hemispherical structures played a key role in enhancing the sensor's sensitivity by providing increased contact area variation during compression [S3].

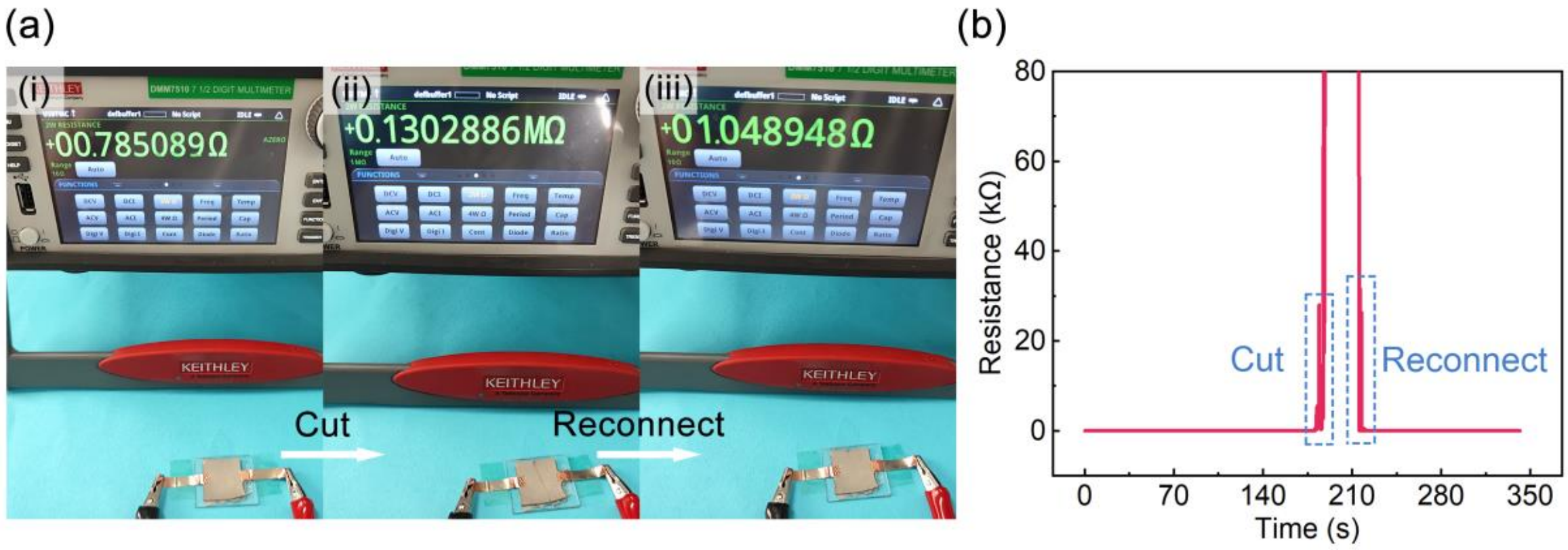

Figure S29. Self-healing properties evaluation of the Ag NWs/GAPB-Gly composite electrodes. (a) Electrical resistance measurements of the initial and healed electrodes. (b) The resistance variation curve during the breaking/healing process, the resistance remained

essentially unchanged after self-healing.

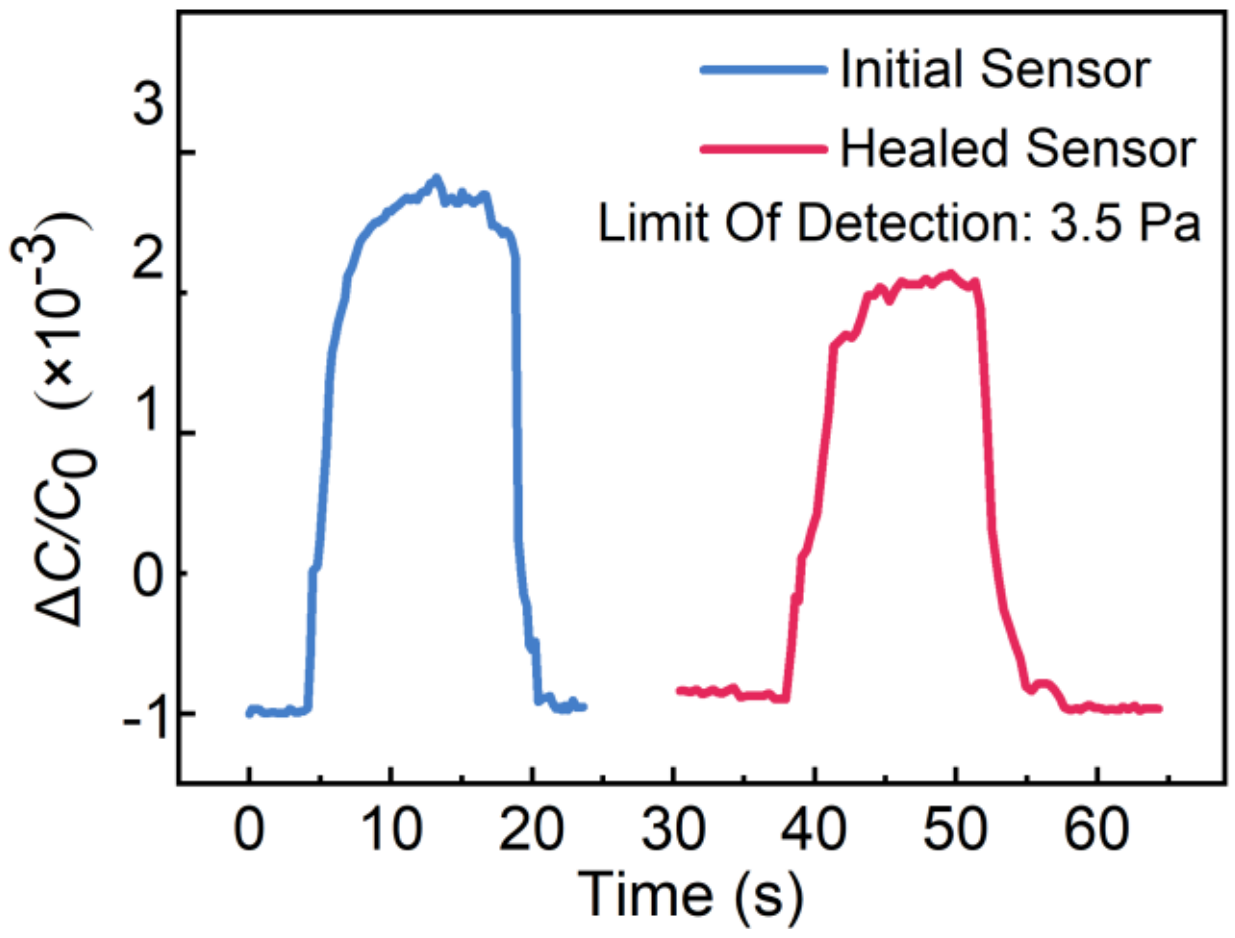

Figure S30. Limit of detection (LOD) testing of the OHI pressure sensor. A glass slide weighing 140 mg and measuring 2×2 cm^2 (equivalent to 3.5 Pa in pressure) was placed on the surface of the sensor [S4].

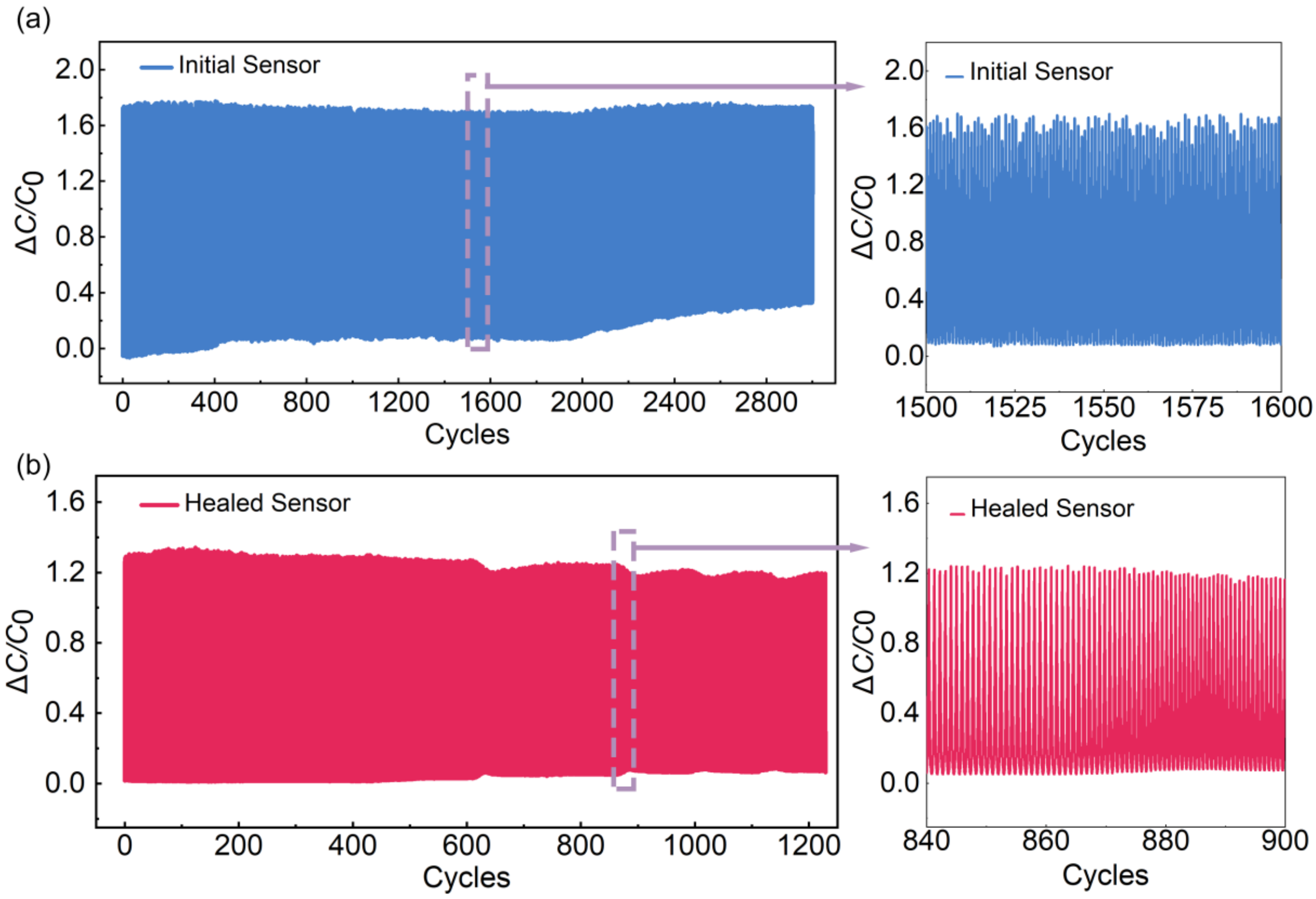

Figure S31. Durability testing of the OHI pressure sensor. The sensor was subjected to a durability test under a pressure of 1 kPa and a compression rate of 2 mm/min. Results indicated that the initial sensor maintained stable signals within 3000 cycles (Figure a). Post-self-healing, the sensor showed no significant deviation in baseline and peak values within 1200 cycles (Figure b). A slight upward shift in the baseline became noticeable after ~2000 cycles, which can be attributed to gradual viscoelastic relaxation of the GAPB-Gly dielectric layer and minor rearrangement of the AgNWs networks under repeated compression.

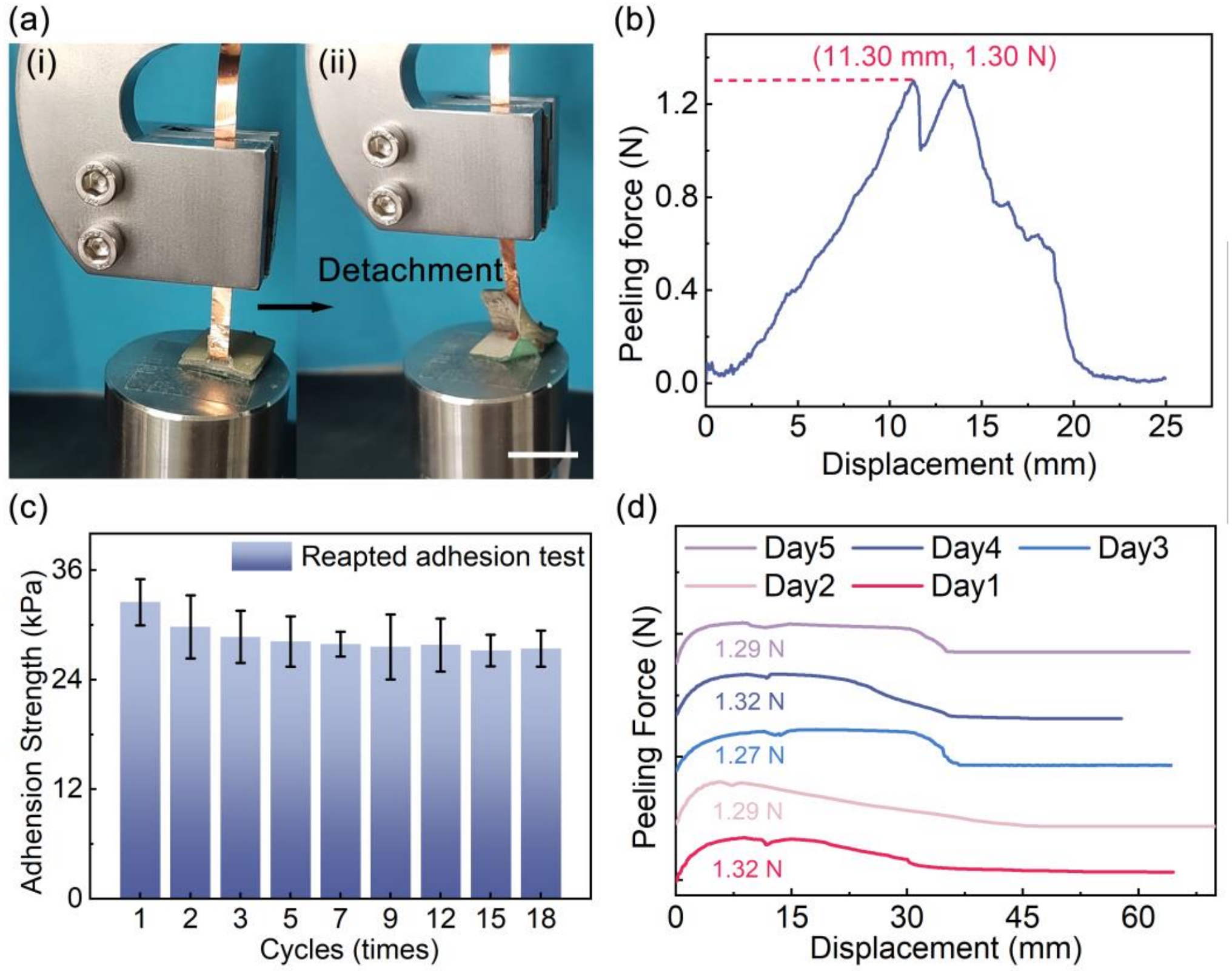

Figure S32. Interlayer bonding strength testing of the OHI pressure sensor. (a) Optical photograph of the peeling test by the universal testing machine (50 mm/min). (b) Interlayer adhesion of the OHI pressure sensor. (c) Interlayer adhesion strength under repeated peeling cycles. (d) Peeling force curve recorded over 5 days.

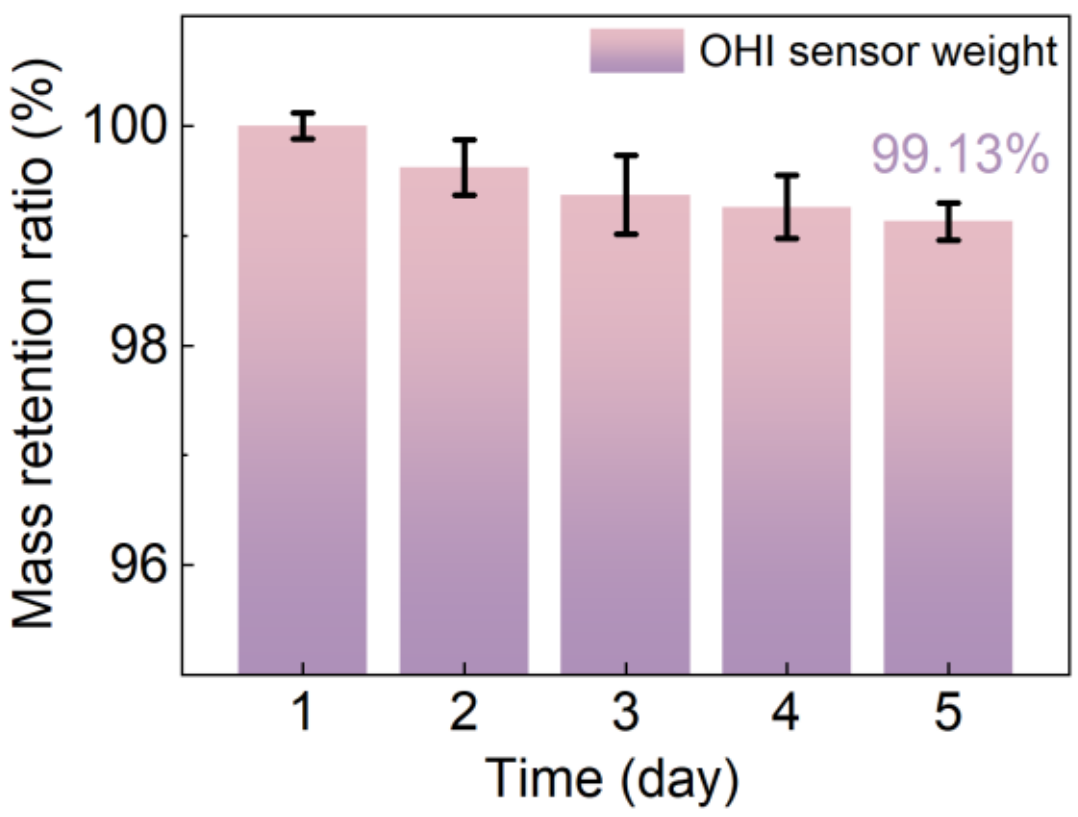

Figure S33. Mass variations of the OHI pressure sensors over a 5-day period. The samples were stored in the constant-temperature incubator at 25 ℃ and 35% relative humidity. n=5.

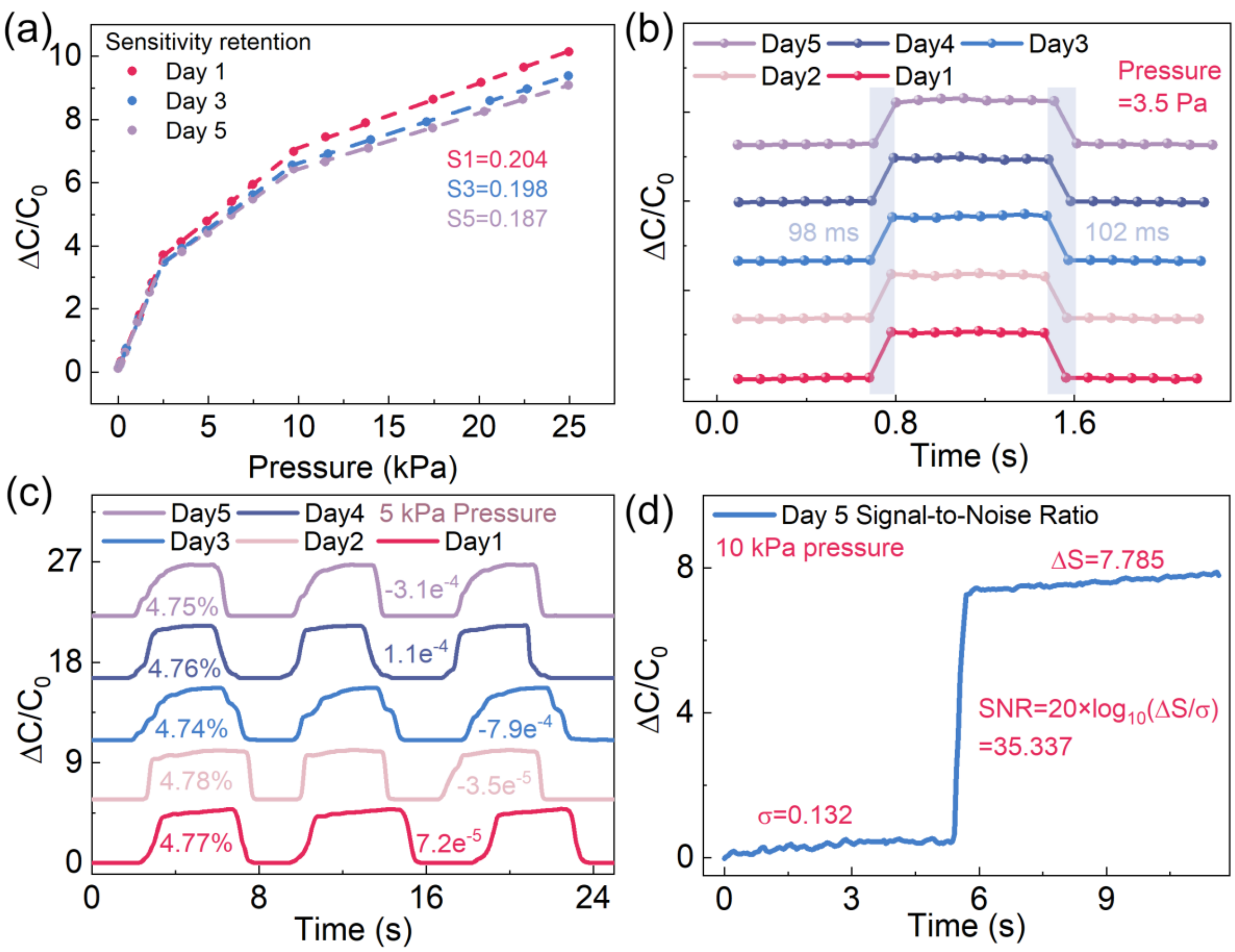

Figure S34. Long-term stability evaluation of the OHI pressure sensors. (a) Evolution of the sensor's sensitivity over a 5-day period. (b) Variations in the response and recovery times over 5 days, characterized by monitoring the dynamic signal upon the loading and unloading of a glass slide (140 mg). (c) Baseline drift monitoring over 5 days. The ultimate capacitance variation upon pressure release consistently fluctuates on the order of 10^{-4}. (d) SNR assessment of the OHI pressure sensor on the 5th day.

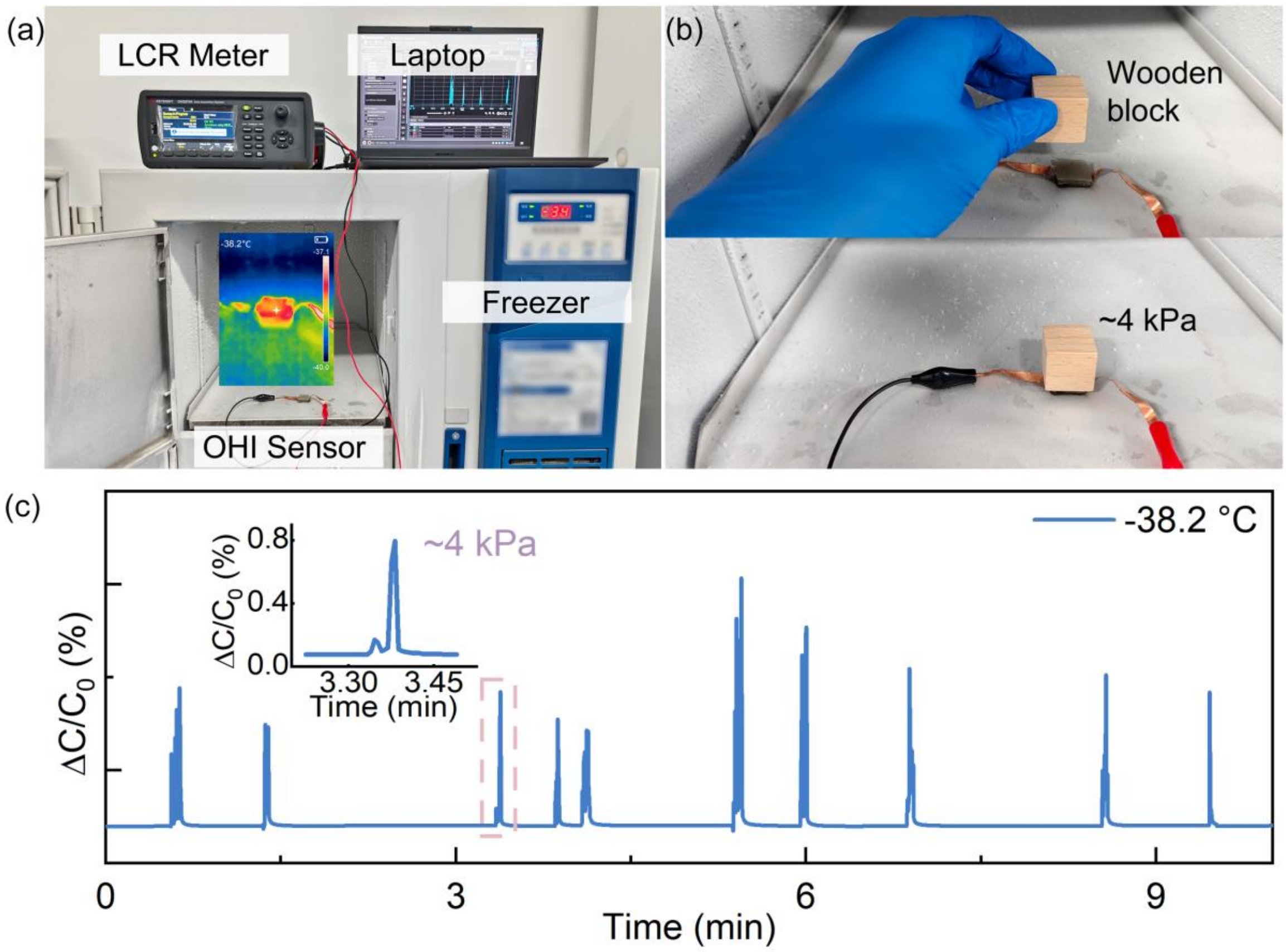

Figure S35. In situ analysis of the OHI pressure sensor's performance under cryogenic conditions. (a) Optical photograph of the experimental setup. The ultra-low-temperature medical freezer was set to -40 ℃, and the sensors were stored at this designated temperature for 24 h prior to measurement. (b) Schematic of the cyclic lift-and-release pressing process. A 10 g wooden block was utilized to continually apply a specific pressure of ~4 kPa. (c) Real-time capacitance variations over a continuous 10-minute testing period.

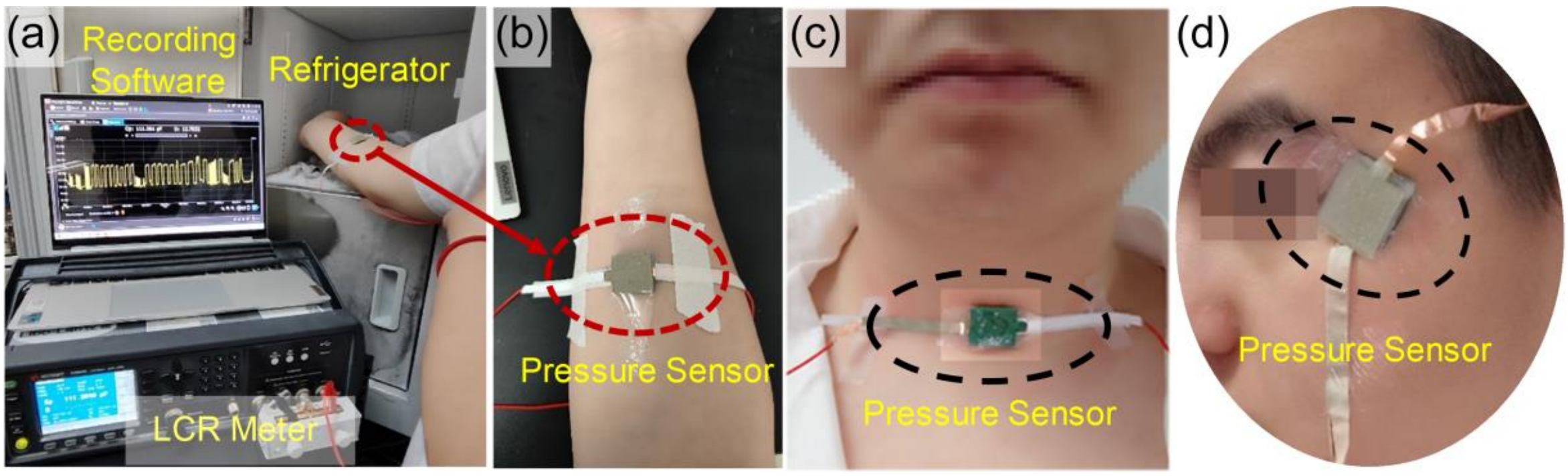

Figure S36. Optical photos of the OHI pressure sensor's signal testing platform and attachment points. (a) Precision LCR meter and recording software to gather pressure signals in human health monitoring applications, while a refrigerator mimicked the low-temperature environments to test the sensor's anti-freezing property. Attachment points of the OHI pressure sensor on human skin: (b) the forearm for assessing muscle movement, (c) the throat for evaluating respiratory and pulse activity, and (d) the eyelid for monitoring ocular fatigue.

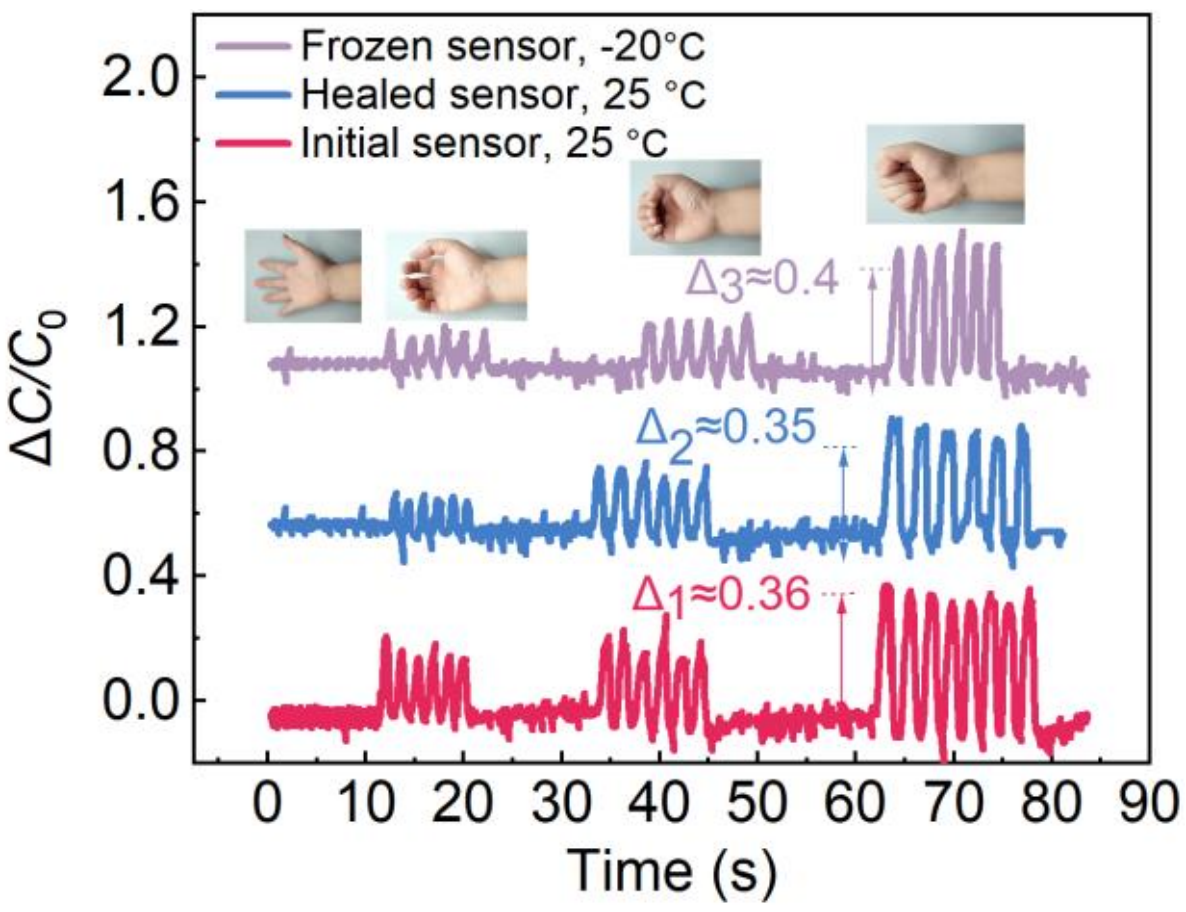

Figure S37. Signals from the initial, healed, and frozen (-20 ℃) OHI pressure sensors when attached to the subject's forearm under different gripping intensities. The capacitance variation amplitudes were proportional to the gripping intensities, which were

0.36, 0.35 and 0.40 for pristine, self-healed, and cryogenically treated OHI pressure sensors, respectively.

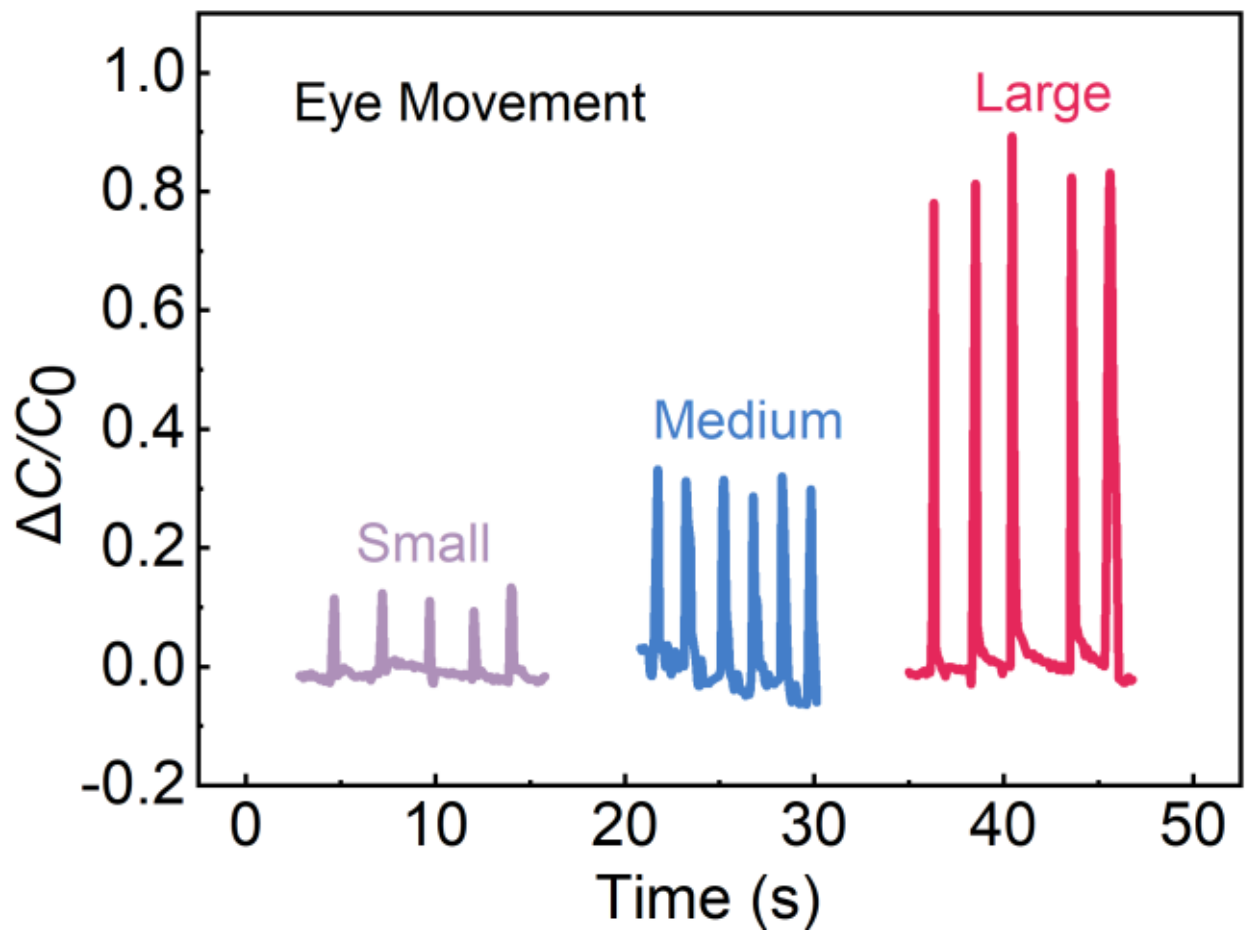

Figure S38. Blinking signals detection when attached the OHI pressure sensor to the eyelid.

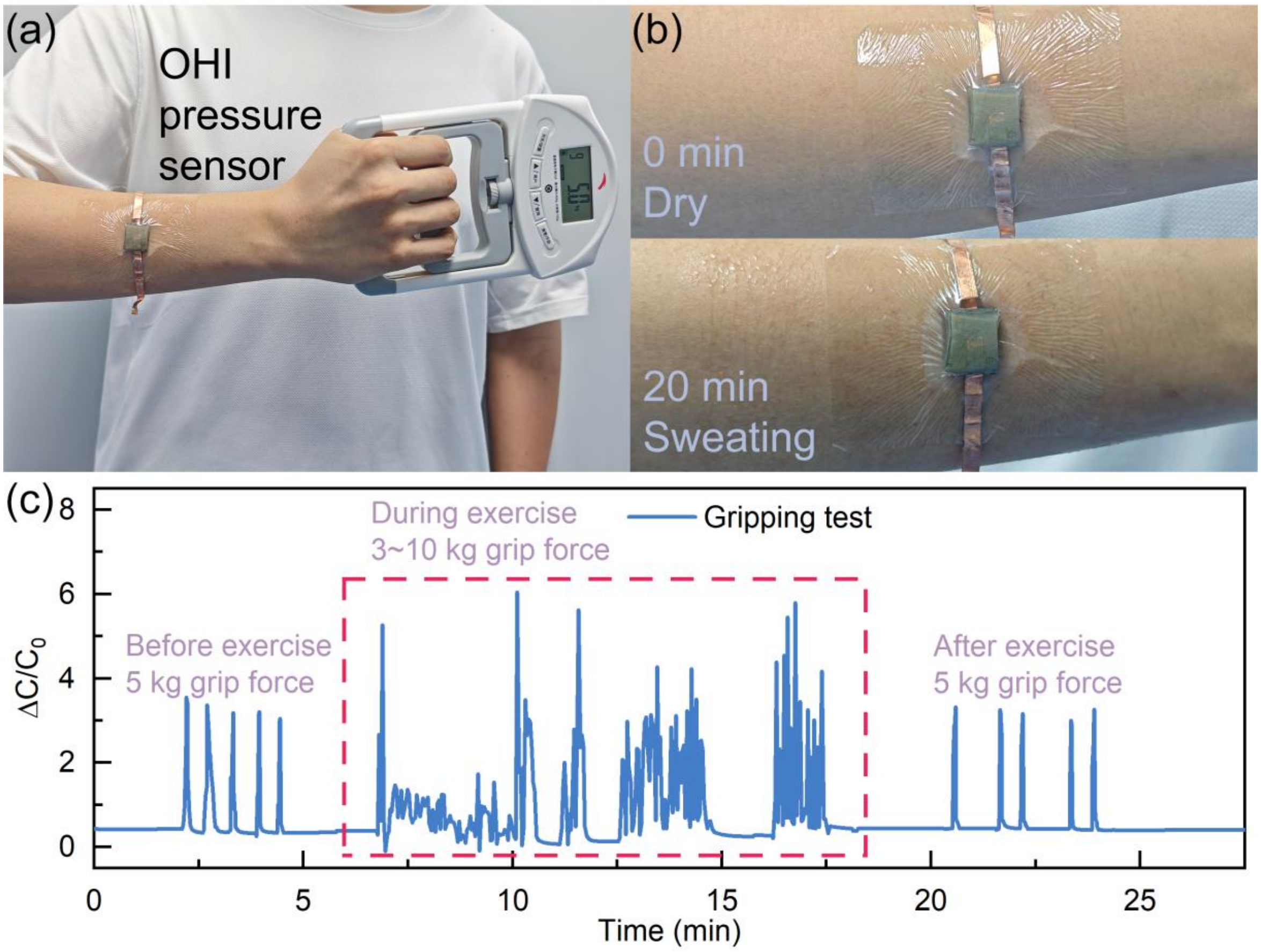

Figure S39. Continuous monitoring of the OHI pressure sensor's performance during long-term skin wear under exercise-induced sweating conditions. (a) Experimental setup for the gripping tests. The sensor was attached to a volunteer's forearm, and a dynamometer (1014351, ANTA Sports Products Limited, China) with a measuring range of 0–20 kg was utilized. (b) Evolution of the skin surface condition over the exercise duration. The skin was initially dry, and showed a sweaty state at 20 min. (c) Capacitance variation curves of the sensor during the prolonged exercise. The volunteer performed repeated gripping with forces ranging from 3-10 kg between 5 and 20 min. The signal variations under a 5 kg gripping force before (3.18%) and after (3.32%) the exercise prove the sensor's stability under long-duration skin wear and sweat interference.

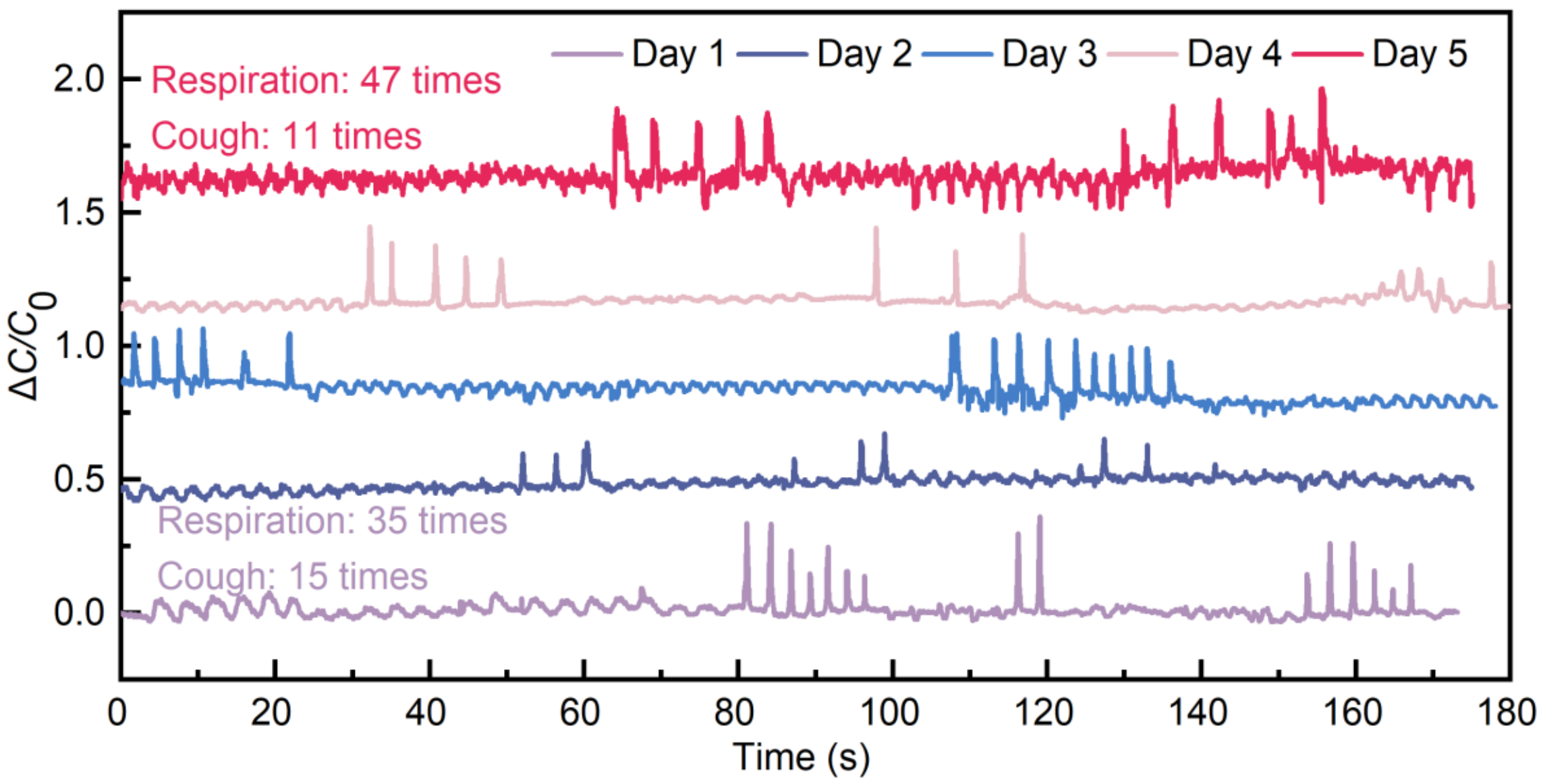

Figure S40. 3 minutes long-term breathing signals monitoring when attached the OHI pressure sensor to the throat.

Supplementary Notes

Note S1 Theoretical definition of the pressure sensitivity of the OHI pressure sensor.

The pressure-sensing mechanism of the double-layer capacitive sensor is governed by a hybrid capacitance model, incorporating both geometric deformation and pressure-modulated electrical double layer (EDL) effects [S5]. The total capacitance under applied pressure *P* could be expressed as equation (1):

$$C = \frac{\varepsilon_r \varepsilon_0 \left(A_0 + \Delta A\right)}{d_0 - \Delta d} + C_{EDL}\left(P\right) \tag{1}$$

where ε_r and ε_0 denoted the relative and vacuum permittivity, respectively; A_0 and d_0 were the initial effective contact area and dielectric layer thickness; ΔA and Δd represented pressure-induced changes in contact area and thickness; $C_{EDL}(P)$ corresponds to the pressure-dependent EDL capacitance, which originates from enhanced ion accumulation and interfacial compression at the electrode/electrolyte interface. Upon pressure application, the soft hydrogel dielectric undergoes notable geometric deformation, including lateral expansion that increases the effective contact area ($A_0+\Delta A$), and vertical contraction that reduces the separation distance ($d_0-\Delta d$). These changes synergistically amplify the geometric capacitance. Simultaneously, the EDL capacitance increases due to the intensified electrostatic interactions and enhanced ionic packing at the interface under compression, further contributing to the overall capacitance response. The normalized sensitivity, accounting for both mechanisms, is quantitatively described as equation (2):

$$S = \frac{1}{C_0} \cdot \frac{dC}{dP} = \frac{1}{C_0}\left(\frac{\partial C_{geom}}{\partial P} + \frac{\partial C_{EDL}}{\partial P} \right) \quad (2)$$

where C_0 is the initial capacitance in the absence of external load. The total sensitivity S thus arises from both the geometric component $\frac{\partial C_{geom}}{\partial P}$, driven by the mechanical deformation of the dielectric layer, and the EDL component $\frac{\partial C_{\mathrm{EDL}}}{\partial P}$, governed by interfacial ionic reconfiguration under pressure. This dual-capacitance modulation ensures an enhanced and tunable pressure response, particularly advantageous in soft ionic systems where both contact mechanics and interfacial electrochemistry play dominant roles [S6].

Supplementary Tables

Table S1. The comparison of multifunctional properties of GAPB-Gly hydrogel with previously reported biocompatible multifunctional hydrogels

Material	Mechanisms	Stretchability (%)	Tensile strength (kPa)	Self-healing rate (%)	Adhesion strength (kPa)	Moisturizing rate (%)	Anti-freezing property (℃)	Biological compatibility	Reference
GelMA/AA/PVA/ Borax/Glycerol	multiple H-bonds, covalent bonds, pH-tuned BEBs, glycerol/H_2O binary solvent	220	183.5	60.8 in 24 h (-40 to 25℃)	≥23.5 to steel after 10 cycles	81 in 10 days (25 ℃, 30% RH)	-55.4	cell viability > 95%	**This work**
PVA/AAm/SBMA/ Borax/LiCl	H-bonds, BEBs, covalent bonds, electrostatics	1350	130	97.0 in 2 h (RT)	20-25 to skin	97 in 7 days (25 ℃, 50% RH)	-45.3	cell viability > 85%	[S7]
TA/CNC/AA/ SBMA/$ZnCl_2$	autocatalysis, robust coordination, H-bonds	~500	103.7	/	10.5 to skin	slight mass increase in 120 days (25 ℃, 50% RH)	-60.0	no skin residue or allergy after 12 h	[S8]
BC/AASP/THMA/ PRP/Glycerol	hard frame-soft penetration, H-bond clusters, glycerol/H_2O binary solvent	~20	7160	/	25.5 to skin	91 in 12 days (open air environment)	-80.0	promotes cell proliferation and migration	[S9]
AAm/Chitosan/NaCl/ 1,2-propanediol	double network, solvent replacement	1400	/	/	10 to metal	70 in 1.5 days	-120.0	/	[S10]
AA/HEMA/MDHS/LiCl/ Glycerol	H-bonds, covalent bonds, electrostatics	11500	225	94.70% for fracture strain	⩽5 to skin	~80 in 30 days (25 ℃, 60% RH)	-35.3	/	[S11]
poly(PDES)/cellulose	H-bonds, covalent bonds, ionic liquids	~1000	~900	83.0 in 24 h (RT)	14.1 to skin	/	-20	/	[S12]
GelMA/PVA/NAGA/ Borax/NaCl	interpenetrating networks, hierarchical H-bonds, covalent bonds	160	~130 kPa	86.4 in 48 h (stress SHE, 25 ℃)	26.5 to Cu tape	/	/	cell viability > 95%	[S13]
AA/PVA/Borax/EG	covalent bond, diverse H-bonds, BEBs, EG/H_2O binary solvent	1410	32.4	73.6 within 10 s (strain SHE, 25 ℃)	27.1 to steel	68.2 in 7 days (70% RH)	-90.0	/	[S14]

GelMA: gelatin methacryloyl, AA: acrylic acid, PVA: poly(vinyl alcohol), AAm: acrylamide, SBMA: 3-[Dimethyl-[2-(2-methylprop-2-enoyloxy) ethyl]azaniumyl]propane-1-sulfonate, TA: tannic acid, CNC: cellulose nanocrystals, BC: bacterial cellulose, AASP: N-acryloyl aspartic acid, THMA: poly-N-(tris[hydroxymethyl]methyl)acrylamide, PRP: platelet-rich plasma, HMA: 2-hydroxyethyl methacrylate, DA: dopamine hydrochloride, BmimCl: 1-butyl-3- methylimidazole chloride, NAGA: N-(2-amino-2-oxoethyl)-2-propenamide, EG: ethylene glycol, RT: room temperature.

Table S2. ICP-OES validation of boron release concentrations from GAPB-Gly hydrogel precursor and synthesized gel at different release times.

Samples	Release time (h)	Sample pretreatment	Boron concentration (mg/L)	Spike recovery (%)	RSD (%)	Method detection limit (mg/L)
Control	1	Direct filtration (0.22 μm)	< 0.01	-	-	0.01
	3		< 0.01			0.01
	6		< 0.01			0.01
	12		< 0.01			0.01
	24		< 0.01			0.01
Precursor sample	1	50-fold dilution (5% HNO_3)	46.5 ± 2.3	96.2	2.1	0.01
	3		41.2 ± 2.1	97.5	1.8	0.01
	6		34.8 ± 1.8	95.8	2.3	0.01
	12		26.3 ± 1.5	96.9	1.9	0.01
	24		18.5 ± 1.2	97.1	2.0	0.01
GAPB-Gly extract	1	Direct filtration (0.22 μm)	0.98 ± 0.06	98.2	1.5	0.01
	3		0.89 ± 0.05	97.8	1.6	0.01
	6		0.73 ± 0.04	99.1	1.4	0.01
	12		0.58 ± 0.04	98.5	1.7	0.01
	24		0.42 ± 0.03	98.9	1.5	0.01

All data are presented as mean ± standard deviation (n = 3). The spike recoveries ranged from 95% to 100% for all time points. Method detection limit (LOD) and limit of quantification (LOQ) were 0.01 mg/L and 0.03 mg/L, respectively, calculated as 3 and 10 times the standard deviation of 11 blank measurements.

Table S3. Performance comparison of OHI sensor with previously reported hydrogel based capacitive pressure sensors.

Material	Structure	Functionality	Sensitivity (kPa^{-1})	Detection range (kPa)	Durability (cycles)	Response time (ms)	Operating life (h)	Ref.
GelMA/AA/PVA /Borax/Glycerol/ NaCl	bi-hemispherical	anti-freezing (-38.2 ℃), self-healing, anti-drying	1.4	0.0035-25	3,000	100	120	**This work**
PVA/PANI	hemisphere	anti-freezing (-18 ℃)	7.7	0-7.4	1,000	/	/	[S15]
ZIF-67/P(AAm-co-HEA)/Glycerol/Choline chloride	film	anti-freezing (-80 ℃), self-healing, anti-drying	0.75	0-56	1000	52.7	24	[S16]
PVA/NaCl	gradient	/	1.09	0.4-800	1000	92	/	[S17]
Polyacrylamide/Alginate	self-buckled	self-healing	3.19	0.025-20	1400	/	/	[S18]
PAA/PEO/PANI	microlattice	/	7.10	0-1.11	/	/	/	[S19]
SF/LNPs	sheet	/	1.32	0-3.8	≥8000	<100	/	[S20]
CNT/PEDOT:PSS-PDMS	micro-dome	self-healing	0.13	0-260	1000	114	/	[S21]

PANI: polyaniline, GO: graphene oxide, PEO: poly(ethylene oxide), SF: silk fibroin, LBPs: lignin nanoparticles, CNT: carbon nanotubes.

Supplementary References

[S1] J. Liu, S. Qu, Z. Suo, W. Yang, *National Science Review* **2021**, *8*, nwaa254.

[S2] D. W. Simmons, D. R. Schuftan, G. Ramahdita, N. Huebsch, *ACS Applied Materials & Interfaces* **2023**, *15*, 25313.

[S3] N. Bai, L. Wang, Y. Xue, Y. Wang, X. Hou, G. Li, Y. Zhang, M. Cai, L. Zhao, F. Guan, X. Wei, C. F. Guo, *ACS Nano* **2022**, *16*, 4338.

[S4] F. He, X. You, W. Wang, T. Bai, G. Xue, M. Ye, *Small Methods* **2021**, *5*, 2001041.

[S5] Y. Chang, L. Wang, R. Li, Z. Zhang, Q. Wang, J. Yang, C. F. Guo, T. Pan, *Advanced Materials* **2021**, 33, 2003464.

[S6] L. Jia, Z. H. Guo, L. Li, C. Pan, P. Zhang, F. Xu, X. Pu, Z. L. Wang, *ACS Nano* **2021**, 15, 19651.

[S7] Y. Wang, P. Chen, X. Zhou, Y. Liu, N. Wang, C. Gao, *ACS Applied Materials & Interfaces* **2022**, 14, 47100.

[S8] Q. Fu, S. Hao, L. Meng, F. Xu, J. Yang, *ACS Nano* **2021**, 15, 18469.

[S9] C. Cai, H. Zhu, Y. Chen, X. Yuan, H. Liu, Z. Yang, *Advanced Healthcare Materials* **2023**, 12, 2301477.

[S10] Y. Liang, Z. Wu, Y. Wei, Q. Ding, M. Zilberman, K. Tao, X. Xie, J. Wu, *Nano-Micro Letters* **2022**, 14, 52.

[S11] X. Guo, F. Yang, X. Sun, Y. Bai, G. Liu, W. Liu, R. Wang, X. He, *Advanced Functional Materials* **2022**, 32, 2201230.

[S12] N. Li, Y. Qiu, J. Ma, L. Qiu, W. Sun, J. Li, Z. Han, W. Chen, X. Ji, *ACS Applied Polymer Materials* **2023**, 5, 9974.

[S13] Z. Li, B. Wang, J. Lu, Y. Xue, J. Wang, B. Jia, G. Han, Y. Zhao, M. A. K. Qureshi, L. Yu, K. Zhao, M. Li, P. Yang, D. Lu, L. Zhao, *Advanced Science* **2025**, 12, e02678.

[S14] X. Su, H. Wang, Z. Tian, X. Duan, Z. Chai, Y. Feng, Y. Wang, Y. Fan, J. Huang, *ACS Applied Materials & Interfaces* **2020**, 12, 29757.

[S15] H. Zhou, M. Wang, X. Jin, H. Liu, J. Lai, H. Du, W. Chen, A. Ma, *ACS Applied Materials & Interfaces* **2021**, 13, 1441.

[S16] MS. Rahman, K. Dixit, MT. Rahman, K. Kim, S. Kim, *Chemical Engineering Journal* **2025**, 510, 161532.

[S17] W. Zhu, J. Wang, W. Sun, S. Zhou, M. He, *Chemical Engineering Journal* **2023**, 451, 138335.
[S18] Y. Wang, M. Tebyetekerwa, Y. Liu, M. Wang, J. Zhu, J. Xu, C. Zhang, T. Liu, *Chemical Engineering Journal* **2021**, 420, 127637.
[S19] M. Yue, Y. Wang, H. Guo, C. Zhang, T. Liu, *Composites Science and Technology* **2022**, 220, 109263.
[S20] L. Wang, Y. Wang, S. Peng, S. Li, S. Wu, *Biomacromolecules* **2025**, 26, 1044.
[S21] J. Guo, L. Zhao, Z. Ouyang, Q. Zuo, Y. Liang, J. Leng, *Advanced Functional Materials* 2026, 36, e25419.
